# PARylation prevents the proteasomal degradation of topoisomerase I DNA-protein crosslinks and induces their deubiquitylation

**DOI:** 10.1038/s41467-021-25252-9

**Published:** 2021-08-18

**Authors:** Yilun Sun, Jiji Chen, Shar-yin N. Huang, Yijun P. Su, Wenjie Wang, Keli Agama, Sourav Saha, Lisa M. Jenkins, John M. Pascal, Yves Pommier

**Affiliations:** 1 Developmental Therapeutics Branch and Laboratory of Molecular Pharmacology, Center for Cancer Research, National Cancer Institute, NIH, Bethesda, MD USA; 2 Advanced Imaging and Microscopy Resource, National Institute of Biomedical Imaging and Bioengineering, NIH, Bethesda, MD USA; 3 Collaborative Protein Technology Resource, Center for Cancer Research, National Cancer Institute, NIH, Bethesda, MD USA; 4 Department of Biochemistry and Molecular Medicine, Université de Montréal, Montreal, QC Canada

**Keywords:** Proteasome, DNA adducts

## Abstract

Poly(ADP)-ribosylation (PARylation) regulates chromatin structure and recruits DNA repair proteins. Using single-molecule fluorescence microscopy to track topoisomerase I (TOP1) in live cells, we found that sustained PARylation blocked the repair of TOP1 DNA-protein crosslinks (TOP1-DPCs) in a similar fashion as inhibition of the ubiquitin-proteasome system (UPS). PARylation of TOP1-DPC was readily revealed by inhibiting poly(ADP-ribose) glycohydrolase (PARG), indicating the otherwise transient and reversible PARylation of the DPCs. As the UPS is a key repair mechanism for TOP1-DPCs, we investigated the impact of TOP1-DPC PARylation on the proteasome and found that the proteasome is unable to associate with and digest PARylated TOP1-DPCs. In addition, PARylation recruits the deubiquitylating enzyme USP7 to reverse the ubiquitylation of PARylated TOP1-DPCs. Our work identifies PARG as repair factor for TOP1-DPCs by enabling the proteasomal digestion of TOP1-DPCs. It also suggests the potential regulatory role of PARylation for the repair of a broad range of DPCs.

## Abstract (web summary)

TOP1 resolves DNA supercoils by forming cleavage complexes (TOP1cc) that are trapped by TOP1 inhibitors. Here the authors provide insights into the mechanistic understanding of TOP1cc PARylation, showing that inhibition of PARG results in stabilization of TOP1cc PARylation that blocks the proteasomal degradation of TOP1cc.

## Introduction

Genomic DNA is constantly challenged by genotoxic agents, leading to various lesions that range from oxidative base modifications, abasic sites, mismatches, chemical adducts, ribonucleotide misincorporation, intra- and inter-strand DNA crosslinks, single-strand breaks (SSBs), double-strand breaks (DSBs), and DNA-protein crosslinks (DPCs)^1^. Among all forms of DNA damage, DPCs are particularly detrimental due to their bulky protein constituents^2^ that obstruct replication and transcription as well as other chromatin-based processes.

Enzymes that act on chromatin and form covalent reaction intermediates with DNA are a primary cause of DPCs^3^. Topoisomerase I cleavage complexes (TOP1cc) are among the most frequent enzymatic DPCs, with the propensity to become abortive in the context of both endogenous DNA lesions and exogenous genotoxic agents^4^.

TOP1 resolves DNA supercoils arising during transcription, replication, and chromatin changes. TOP1 acts by creating SSBs allowing rotation of the cut strand around the intact strand (swivel). DNA cleavage is a transesterification reaction in which TOP1’s active tyrosine attacks the DNA phosphodiester bond. This forms a transient DPC at the 3′-end of a SSB named a TOP1cc, which is reversible upon the rejoining of the broken DNA ends of the TOP1cc. In addition to endogenous and environmental DNA lesions, TOP1 is the target of anticancer drugs that kill cancer cells by trapping TOP1ccs^4^. For example, camptothecin (CPT) and its clinical derivatives directly block the rejoining step of TOP1ccs and lead to the immediate formation of persistent TOP1ccs (which we also refer to as TOP1-DPCs)^5^. Unrepaired TOP1-DPCs have been implicated in neurological disorders, R-loop formation, and selective susceptibility of cancer cells to clinical TOP1 inhibitors^4,6–10^, especially when combined with PARP inhibitors^11,12^.

TOP1-DPC are repaired by redundant pathways^3,13^ regulated by post-translational modifications (PTMs); of which ubiquitylation plays a dominant role by inducing the proteasomal degradation of TOP1-DPCs^3^. TOP1-DPCs are ubiquitylated either in replication/transcription-dependent manners^14,15^ or in response to SUMO modifications^16^. TOP1-DPC polyubiquitylation is required for the 26S proteasome-mediated destruction of the bulky protein component of the DPCs, a pivotal step allowing tyrosyl-DNA phosphodiesterase 1 (TDP1) to access and hydrolyze the otherwise buried TOP1-DPC phosphotyrosyl linkage^17,18^. This choreographed repair pathway also involves PARP1-dependent poly(ADP-ribosyl)ation (PARylation) that modifies TDP1 and recruits it to TOP1-DPC sites^19,20^.

Yet, whether TOP1-DPC is a target of PARP1 and PARylation for its repair remains understudied and direct evidence for PARylation of the DPCs in cells is lacking^20–24^. PARP1 serves as a first responder that senses SSBs and promotes repair pathway choice in part through PARylation^12,25^. It was initially reported that PARP1 PARylates human TOP1 but does not impact its religation activity in vitro^22,24^, whereas later studies suggested that TOP1 PARylation enhanced the religation^24,26^. In addition, PARP1 modulates TOP1 subnuclear translocation in response to CPT^20^, suggesting a broad role of PARylation in the repair of TOP1-DPCs. Here, we investigated whether PARP1 directly PARylates TOP1-DPCs and if so, how this modification is regulated and integrated for the proteasomal degradation and repair of TOP1-DPCs.

## Results

### Single-molecule tracking identifies PARG as a TOP1-DPC repair factor

To observe the subcellular dynamics of TOP1-DPCs, we set up a single-molecule fluorescence microscopy technique to track TOP1 single molecules in live cells. We constructed a plasmid expressing human TOP1 fused with HaloTag^27^, a self-labeling protein tag, at its carboxyl terminus via Golden GATEway cloning^28^ (Supplementary Fig. 1a, b). Following transfection of the construct into human osteosarcoma U2OS cells, we added the Janelia Fluor 549 ligand to the culture medium, which covalently binds the HaloTag reporter^29^. Imaging TOP1-HaloTag single molecules using a custom-built Nikon Ti microscope showed that a large fraction of nuclear TOP1 was highly dynamic (Supplementary Fig. 1c, d and Supplementary Movie 1). The jump distance of TOP1-HaloTag single molecules was well distributed and ranged from 0.1 to 1.2 μm (Supplementary Fig. 1e).

**Fig. 1:**
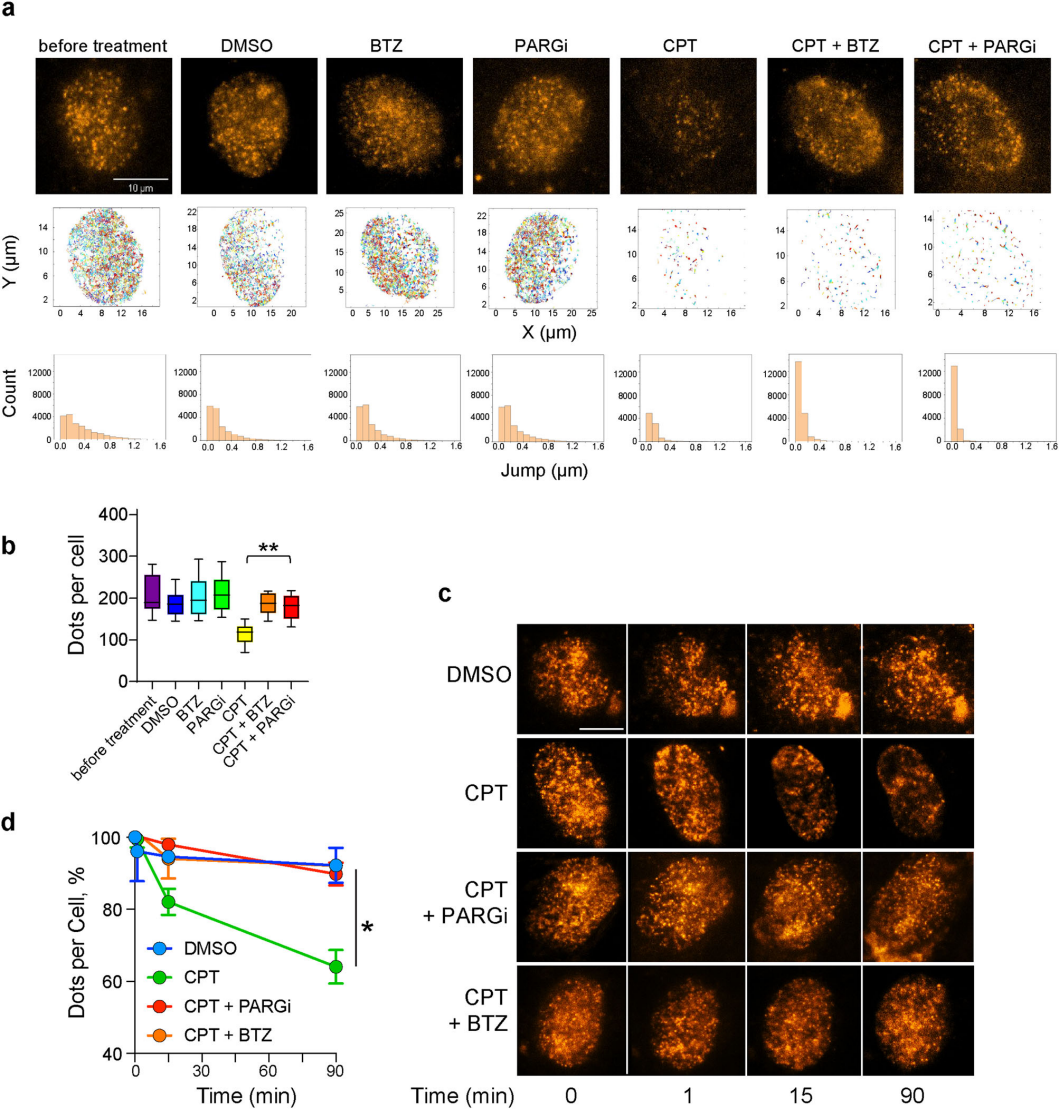
Identification of PARG as repair factor for TOP1-DPC by single-molecule tracking. **a** Top panels: filming of TOP1-HaloTag single molecules in U2OS cells transfected with TOP1-HaloTag expression plasmid. The cells were divided into seven groups: no treatment, DMSO (2h), BTZ (10 μM, 2h), PARGi (10 μM, 2h),  CPT (100 μM, 2h), CPT + BTZ and CPT + PARGi. Middle panels: plots of tracks of TOP1-HaloTag single molecules in the top-panel films. The tracks were reconstructed in two dimensions by MATLAB analysis pipeline. The neighboring tracks are of different colors to distinguish one from the others. Bottom panels: count of jumps of TOP1-HaloTag single molecules derived from the top-panel films. The *X*-axis of the histogram is the jump distance of TOP1 single molecules in the top-panel films and the *Y*-axis is the count of the jumps. The bin size is 0.1 μm. For example, the “before treatment” sample has 4120 jumps whose distances are between 0 and 0.1 μm and 86 jumps whose distances are between 1 and 1.2 μm. The sample has a total of 19120 jumps that are the sum of the count of each bin. **b** Quantitation of TOP1-HaloTag single molecules shown in panel (**a**) using ThunderSTORM, an ImageJ plug-in. Top lines are maximum values, bottom lines are minimum values and middle lines are medians. Box limits indicate the range of the central 50%. Whiskers are upper and lower quartiles. *n* = 10 biologically independent samples. *P* value was calculated by paired Student’s t-test (two-tailed distribution). **: *p* = 0.0017. **c** Time-course of TOP1-HaloTag single molecules in U2OS cells treated with DMSO, CPT (100 μM), CPT (100 μM) + PARGi (10 μM) and CPT (100 μM) + BTZ (10 μM). Images were taken at the indicated time points. The scale bar represents 10 μm. **d** Quantitation of TOP1-HaloTag single molecules shown in panel (**c**). *n* = 2 independent experiments. Data are presented as mean values +/− standard deviation (SD). *P* value comparing TOP1 single molecules of CPT and CPT + PARGi was calculated by paired Student’s t-test (two-tailed distribution). *: *p* = 0.038.

To determine the effects of TOP1 trapping (TOP1-DPC induction), we exposed the TOP1-HaloTag-expressing U2OS cells to CPT. Exposure to CPT for 2 h immobilized the vast majority of TOP1 single molecules (Fig. 1a; Supplementary Movie 6), consistent with the trapping of TOP1 on DNA. In addition, CPT produced a 45% reduction in the levels of TOP1 single molecules (Fig. 1b) in comparison with cells before treatment (Fig. 1a; Supplementary Movie 2). By contrast, DMSO treatment (solvent for CPT) did not affect the dynamics of TOP1 or the overall number of TOP1 proteins (Fig. 1a, b; Supplementary Movie 3).

As the ubiquitin-proteasome system is a pivotal step in the degradation and repair of TOP1-DPCs^3,16,21^, we pre-treated the cells with the proteasome inhibitor bortezomib (BTZ) and found, as expected, that BTZ blocked the overall downregulation of TOP1 single molecules in response to CPT but BTZ alone did not impact either the levels or the dynamics of TOP1 single molecules (Fig. 1a, b; Supplementary Movies 4 and 7).

To examine the impact of PARylation on TOP1, we pre-treated the cells with a selective and potent poly(ADP-ribose) glycohydrolase inhibitor (PARGi, PDD00017273) to block potential dePARylation thereby stabilize PARylation^30^. While the PARGi alone did not change TOP1 protein levels and dynamics, like BTZ, it blocked CPT-induced TOP1 downregulation without affecting the reduced mobility of TOP1 induced by CPT (Fig. 1a, b; Supplementary Movies 5 and 8). In addition, the time-course study of TOP1 single molecules tracking further demonstrates that PARGi prevents CPT-induced cellular TOP1 downregulation (Fig. 1c, d; Supplementary Movies 9−12). These data suggested that dePARylation was likely required for TOP1 proteasomal degradation in response to TOP1-DPC induction and provided evidence of the role of PARG for the repair of TOP1-DPCs.

### PARylation of TOP1-DPCs by PARP1 is transient and prevents the removal of TOP1-DPCs

**Fig. 2:**
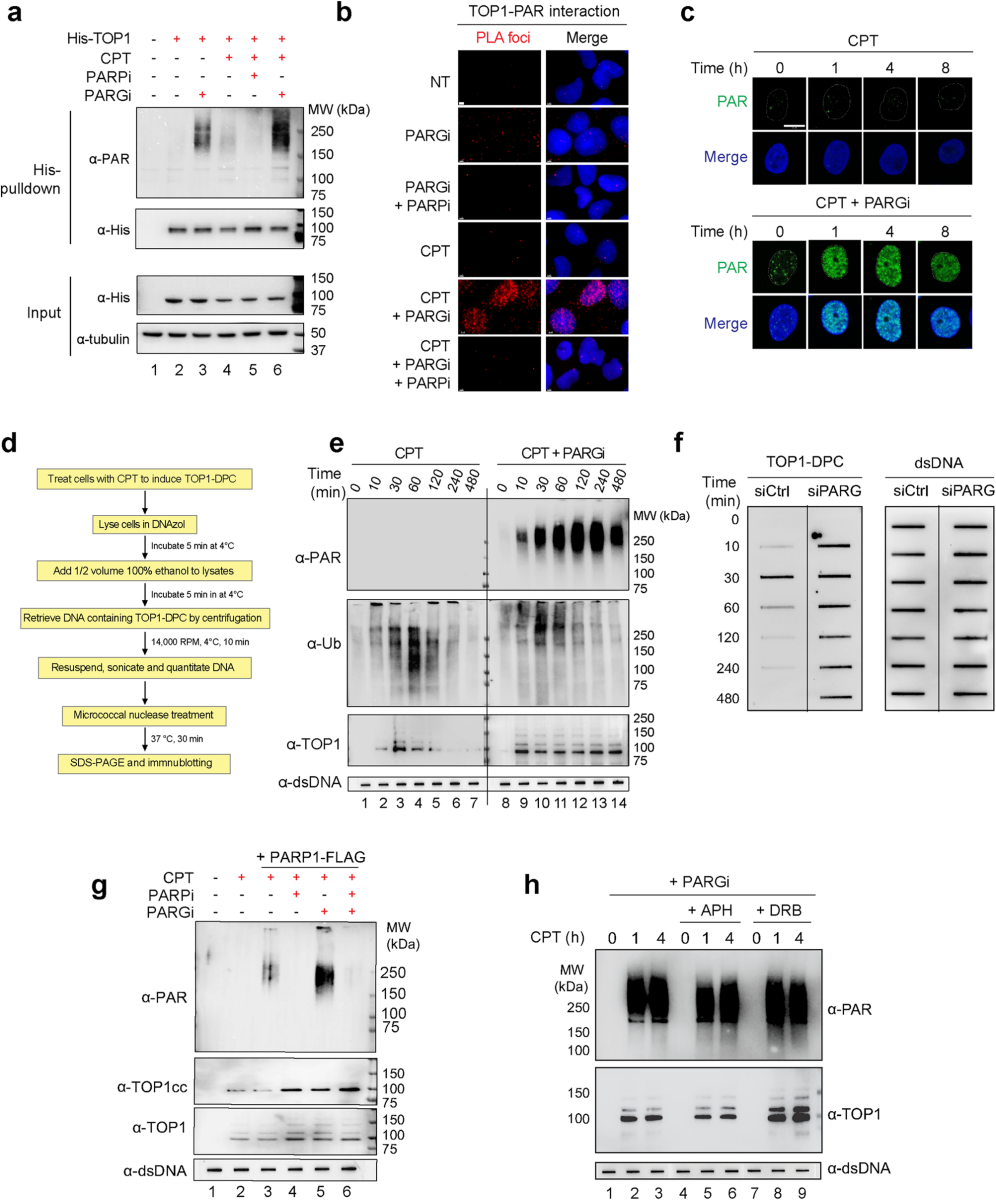
Inhibiting PARG reveals the otherwise transient PARylation of TOP1-DPC and stabilizes TOP1-DPCs. **a** His-tag pulldown assay showing reversible PARylation of transfected His-TOP1. Following transfection of 6×His-tagged TOP1 expression construct, HEK293 cells were treated with the indicated drugs for His-tag pulldown using Ni-NTA agarose under denaturing conditions. The pulldown samples and input samples were subjected to IB using α-TOP1 and α-PAR antibodies. CPT: 20 µM, 30 min; PARPi: 10 µM, 1 h pre-treatment; PARGi: 10 µM, 1 h pre-treatment. **b** Proximity ligation assay (PLA) showing TOP1-PAR interaction in cells treated with CPT and PARGi. Following transfection of 6×His-tagged TOP1 expression construct, U2OS cells were treated with the indicated drugs for PLA using rabbit α-His-tag antibody and mouse α-PAR antibody (10H). The scale bar represents 3 μm. **c** PARGi revealed CPT-induced PAR polymers on chromatin. After pre-extraction, U2OS cells treated as indicated were subjected to IF using α-PAR antibody (10H). CPT: 20 µM; PARGi: 10 µM, 1 h pre-treatment. The scale bar represents 10 μm. **d** Scheme of immunodetection of TOP1-DPC PARylation in vivo by modification of RADAR assay. TOP1-DPC PARylation was detected with α-PAR antibody (10H). **e** PARGi induced the hyper-PARylation and stabilization of TOP1-DPCs. HEK293 cells were treated with CPT (20 µM) in the absence or presence of PARGi (10 µM, 1 h pre-treatment). Cells were collected at the indicated time points for the modified RADAR for detection of TOP1-DPCs and their PARylation and ubiquitylation using α-TOP1, α-PAR, and α-Ub antibodies. 2 µg of digested DNA from each sample was subjected for slot-blotting using α-dsDNA antibody as a loading control. **f** ICE assay confirming that PARG deficiency blocked the removal of TOP1-DPCs. HEK293 cells transfected with control siRNA (siCtrl) or siRNA targeting PARG were treated with CPT (2 µM). Cells were collected at the indicated time points for ICE assay. 2 µg of digested DNA from each ICE assay sample was subjected for slot-blotting using α-TOP1 antibody or α-dsDNA antibody. **g** The modified RADAR assay showing that in vivo TOP1-DPC PARylation by PARP1 was counteracted by PARG. After transfection of the PARP1-FLAG expression plasmid, HEK293 cells were pre-treated with PARPi (10 µM, 1 h), PARGi (10 µM, 1 h), or PARPi + PARGi, followed by CPT treatment (20 µM, 1 h). The cells were then subjected to the modified RADAR assay for detection of TOP1-DPCs and their PARylation using α-TOP1 and α-PAR antibodies. **h** Inhibiting replication or transcription did not affect TOP1-DPC PARylation. HEK263 cells were pre-treated PARGi (10 µM, 1 h) in the absence or presence of the replication inhibitor aphidicolin (APH, 10 µM, 1 h) or the transcription inhibitor DRB (100 µM, 1 h). Cells were co-treated with 20 µM CPT for 0, 1, or 4 h, followed by detection of TOP1-DPCs and their PARylation by the modified RADAR assay using α-TOP1 and α-PAR antibodies.

To interrogate the role of PARG in the repair of TOP1-DPCs, we performed His-tag pulldown in human embryonic kidney cells HEK293 transfected with His6-TOP1 expression plasmid followed by immunoblotting (IB) with anti-PAR antibody. While PARylation of His6-TOP1 was nearly undetectable under unperturbed conditions (Fig. 2a, lane 2), it became readily detectable upon exposure to PARGi (Fig. 2a, lane 3). Short treatments with CPT (30 min) induced low-level TOP1 PARylation (Fig. 2a, lane 4), which was suppressed by the PARP inhibitor talazoparib (Fig. 2a, lane 5) and markedly enhanced by the PARGi (Fig. 2a, lane 6). To determine whether free TOP1 is a target for PARylation, we performed His-tag pulldown in HEK293 cells transfected with His6-TOP1 WT and catalytic mutant (Y723F). Immunoblotting with anti-PAR antibody showed that TOP1 WT but not the Y723F mutant was PARylated when the cells were treated with PARGi (Supplementary Fig. 2a), indicating that TOP1 PARylation requires TOP1 cleavage activity.

To study the potential impact of TOP1 PARylation on the formation of TOP1-DPCs in biochemical assays, we employed a 3′-^32^P-labeled DNA oligonucleotide substrate (117 bp) encompassing previously characterized TOP1 cleavage sites and recombinant TOP1^31^. PARylation of TOP1 by recombinant PARP1 prior to incubation with the substrate did not affect the levels of TOP1-cleaved DNA products both in the absence and presence of CPT (Supplementary Fig. 2b, lanes 1−4). Similarly, PARylation of TOP1 20 min after the incubation with the substrate and CPT did not change the levels of TOP1-cleaved DNA products (Supplementary Fig. 2b, lanes 5 and 6). These experiments rule out the possibility that PARylation of TOP1 enhances its cleavage activity^24^ and therefore suggest that in vivo TOP1 PARylation observed in the absence of CPT is a likely response to low levels of endogenous and spontaneous TOP1-DPCs^4,6^.

Proximity-ligation assays (PLA) in His6-TOP1-expressing U2OS cells using anti-His tag antibody and anti-PAR antibody (10H) confirmed the interaction between TOP1 and PAR polymers induced by co-treatment with CPT and PARGi (Fig. 2b and Supplementary Fig. 2c, d). Immunofluorescence (IF) microscopy of pre-extracted U2OS cells with anti-PAR antibody (10H) showed that CPT-induced PARylation could not be detected in the absence of PARGi (Fig. 2c), indicating quickly reversible PARylation of chromatin-bound proteins in response to TOP1-DPCs.

Because it was unknown whether cellular TOP1-DPCs are directly modified by PAR polymers, we detected PAR-TOP1-DPCs in vivo by adapting the RADAR (rapid approach to DNA adduct recovery) assay^32^ (Fig. 2d), as we had done previously for the detection of ubiquitylated or SUMOylated TOP-DPCs^16^. In brief, we treated HEK293 with CPT to induce TOP1-DPCs and lysed the cells with the chaotropic agent DNAzol to disrupt non-covalent protein−protein and protein−DNA interactions. Genomic TOP1-DPCs were purified by ethanol precipitation and digested with micrococcal nuclease to release the DPCs. The digested samples were subjected to Western blotting (WB) for detection PTMs of the DPCs using antibodies targeting the PTMs. Consistent with our recent findings^16^, CPT rapidly induced TOP1-DPCs with a peak level at 30 min and disappearance within 4 h (Fig. 2e, 3rd panel from the top, lanes 1−7). By probing the modified RADAR samples with anti-Ub antibody, we confirmed the rapid and transient appearance of poly-ubiquitylated species indicative poly-Ub-TOP1-DPCs upon CPT treatment (Fig. 2e, 2rd panel from the top, lanes 1−7) and their repair by the ubiquitin-proteasome system^16^.

Probing the modified RADAR assay samples from cells only treated with CPT with anti-PAR antibody did not detect any signal (Fig. 2e, top panel, lanes 1−7), consistent with the possibility that TOP1-DPCs undergo transient PARylation, which cannot be detected without blocking PARG. To confirm this possibility, we pre-treated the cells with PARGi before performing the modified RADAR assay. PARGi treatment led to readily detectable PARylated TOP1-DPC as soon as 10 min after CPT treatment (Fig. 2e, upper right panel, lane 9). Those PARylated TOP-DPCs gradually increased and peaked at 2 h (Fig. 2e, lane 13). Probing the PARGi-pretreated modified RADAR samples with anti-Ub antibody showed that PARGi did not prevent TOP1-DPC ubiquitylation (Fig. 2e, 2nd panel from the top, lanes 8−14). However, PARG inhibition facilitated TOP1-DPC ubiquitylation (it peaked at 30 min) and decreased the ubiquitylation 1 h after CPT treatment. Also, the ubiquitylated TOP1-DPC species from PARGi-pretreated samples showed slower electrophoretic migration than those without PARGi pre-treatment, suggesting that the ubiquitylated TOP1-DPC species in PARGi-pretreated samples were PARylated, resulting in increased molecular weights and decreased electrophoretic mobility.

To assess the impact of PARG inhibition on TOP1-DPCs, we probed the samples with an anti-TOP1 antibody. Of note, PARGi completely prevented the disappearance of CPT-induced TOP1-DPCs (Fig. 2e, 3rd panel from the top, compare lanes 9−14 with lanes 2−7), suggesting that PARG is required for the repair of TOP1-DPCs. Given the crucial role of the 26S proteasome system for TOP1-DPC repair^3,16^, we hypothesized that persistent PARylation likely prevents the proteasome-dependent removal of TOP1-DPCs.

To confirm the PARylated species detected by the modified RADAR assay are PAR-TOP1-DPCs, we knocked down TOP1 using siRNA in HEK293 cells and found that neither TOP1-DPCs nor the PARylated species were detected by the modified RADAR assay (Supplementary Fig. 2e), indicating that the PARylation is specific to TOP1-DPCs.

Furthermore, we consolidated the role of PARG for the resolution of TOP1-DPCs by performing in vivo complex assay, the original bioassay for topoisomerase-DPC immunodetection^32^, in HEK293 cells transfected with control siRNA or with siRNA against PARG (Supplementary Fig. 2f). As expected, the levels of CPT-induced TOP1-DPC reached a peak at 30 min after CPT exposure and gradually diminished with time in control cells whereas CPT-induced TOP1-DPC remained unresolved even after 480 min after CPT treatment in PARG deficient cells (Fig. 2f; Supplementary Fig. 2g).

It has also been suggested that PARylation of TOP1 may play a role for the recruitment of TOP1 to the active sites of rDNA synthesis^33^. To determine whether the stimulation of CPT-induced TOP1-DPCs by PARGi is in part due to dysregulation in TOP1 subnuclear distribution or elevation in TOP1 chromatin localization, we conducted a subcellular fractionation assay in HEK293 cells and found that PARGi did not alter the levels of chromatin-bound TOP1 before and after acute CPT treatment (Supplementary Fig. 2h). This observation further confirms that persistent PARylation blocks the removal of TOP1-DPCs rather than enhancing TOP1-DPC formation.

To exclude the possibility that the accumulation of PARylated TOP1-DPC resulted from an increase in total TOP1-DPCs in cells treated with PARGi, we performed the modified RADAR assay in cells pre-treated with PARGi, PARPi, or BTZ that blocks TOP1-DPC degradation. The elevation of CPT-induced TOP1-DPCs by pre-treatment with BTZ or PARP1 did not result in detectable TOP1-DPC PARylation (Supplementary Fig. 2i), demonstrating that the accumulation of TOP1-DPCs upon pre-treatment with PARGi is due to the inhibition of dePARylation.

To confirm that PARP1 catalyzes the PARylation of TOP1-DPC in vivo, we performed the modified RADAR assay in HEK293 cells transfected with FLAG-tagged PARP1 (Supplementary Fig. 2j). In the absence of PARGi, PARP1 upregulation resulted in weak TOP1-DPC PARylation (Fig. 2g, lane 3), which was suppressed by PARPi (Fig. 2g, lane 4). TOP1-DPC PARylation was largely enhanced by PARGi and abolished by co-treatment with PARPi (Fig. 2g, lanes 5 and 6). These results taken together show that PARP1 PARylates CPT-induced TOP1-DPC, and that PARylation is normally transient and readily reversed by PARG.

To examine whether TOP1-DPC PARylation, like their ubiquitylation, requires ongoing DNA transactions for activation^14,15^, we pre-treated HEK293 cells with the replication inhibitor aphidicolin (APH) and the transcription inhibitor DRB (5,6-dichloro-1-beta-D-ribofuranosylbenzimidazole). Neither affected TOP1-DPC PARylation (Fig. 2h), indicating that the induction of TOP1-DPC PARylation is not contingent on encounters between the DPCs and ongoing DNA metabolic processes.

High-performance liquid chromatography-mass spectrum (HPLC-MS) analyses in His-TOP1 expressing human colorectal carcinoma HCT116 cells also showed that PARP1 is enriched in His-TOP1 pulldown sample even in the absence of CPT (Supplementary Data 1), consistent with the fact that PARP1 and TOP1 are associated under unperturbed conditions^22,34^, and that PARP1 promptly PARylates TOP1-DPCs without the requirement for replication or transcription.

### TOP1-DPC dePARylation and degradation are required for the activity of cellular TDP1

**Fig. 3:**
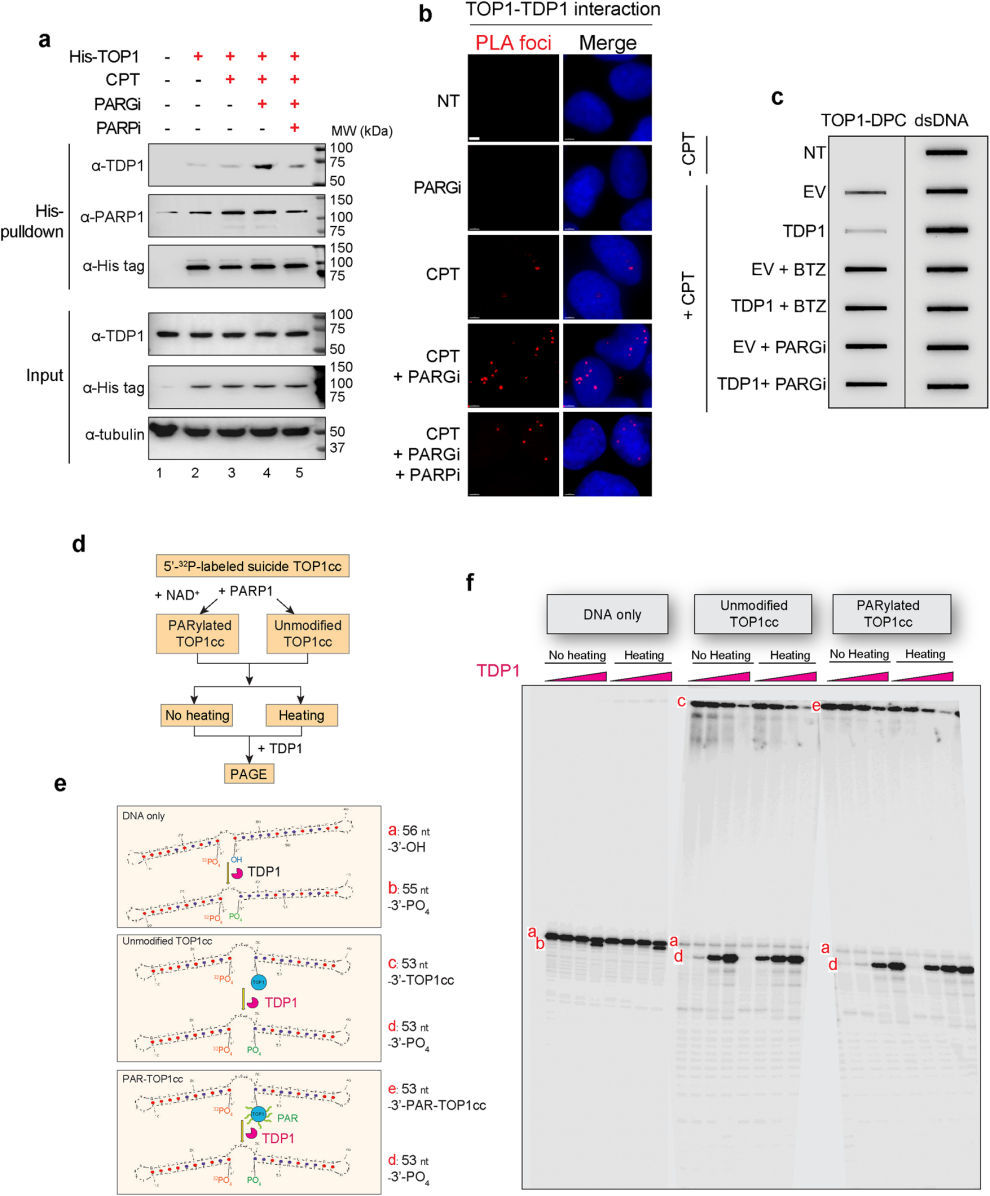
TOP1-PARylation enhances TDP1 recruitment without enhancing TOP1cc hydrolysis. **a** TOP1-TDP1 interaction was enhanced by PARGi and inhibited by PARPi. Following transfection of 6×His-tagged TOP1 expression construct, HEK293 cells were treated with the indicated drugs before His-tag pulldown using Ni-NTA agarose under native conditions. The pulldown and input samples were Western blotted with the indicated antibodies. **b** Proximity Ligase Assay (PLA) confirming that inhibiting PARG enhanced TOP1-TDP1 interactions. U2OS cells were treated with CPT (20 µM, 1 h), PARGi (10 µM, 2 h), or CPT + PARGi (pre-treatment with PARGi for 1 h then co-treatment with CPT and PARGi for 1 h), followed by PLA assay. The scale bar represents 3 μm. **c** ICE assay showing that TDP1 failed to resolve hyper-PARylated TOP1-DPCs in vivo. HEK293 cells transfected with empty vector (EV) or TDP1-FLAG overexpression plasmid were treated with CPT (2 µM) for 1 h in the absence or presence of PARGi or BTZ. Cells were collected for ICE assay. 2 µg of digested DNA from each ICE assay sample was subjected for slot-blotting using α-TOP1 antibody or α-dsDNA antibody. **d** Scheme for [^32^P]-labeled TOP1cc preparation, PARylation, and TDP1 digestion assays. **e** Schematic representation of the reaction products generated by TDP1 with the DNA suicidal substrate alone, unmodified TOP1ccs, and PARylated TOP1ccs. **f** TDP1 acted on unmodified and PARylated TOP1-DPCs at high concentrations in vitro. Representative TDP1 assay as described in panel (**d**) showing the 3′-nucleosidase activity of TDP1 (left set of eight samples) and TDP1’s phosphodiesterase activity toward unmodified TOP1-DPC (middle 8 samples), which is facilitated by heat denaturation of TOP1cc but does not exhibit significantly altered  activity toward PARylated-TOP1cc (right eight samples). TDP1 concentrations: 0, 1.25, 25 and 500 nM.

Tyrosyl-DNA phosphodiester 1 (TDP1) repairs TOP1-DPCs by hydrolyzing their 3′-phosphotyrosyl bond^13,18^. We previously reported that PARP1 recruits TDP1 to TOP1-induced damage sites and stabilizes TDP1 protein levels^19^. To examine whether PARG affects TDP1-mediated resolution of TOP1-DPCs, we transfected cells with His-tagged TOP1 and performed His-pulldown to detect TOP1-TDP1 interaction. A weak interaction was detected both in the absence and presence of CPT (Fig. 3a, lanes 2 and 3). This interaction was largely potentiated by inhibiting PARG before CPT treatment (Fig. 3a, lane 4), and suppressed by the PARPi (Fig. 3a, lane 5), suggesting that persistent PARylation potentiates the recruitment of TDP1 to TOP1 upon exposure to CPT. Consistently, PLA assay in cells co-transfected with His-TOP1 and TDP1-FLAG showed that TDP1 interacts with TOP1 upon CPT treatment in a PARylation-dependent manner (Fig. 3b; Supplementary Fig. 3a).

### TOP1-DPC PARylation blocks the cellular activity of TDP1 but does not significantly affect the phosphodiesterase activity of TDP1 in vitro

We next asked whether the enhanced PAR-mediated interaction of TOP1 and TDP1^19,20^ impacts the activity of TDP1 toward TOP1-DPCs. To do so, we transfected HEK293 cells with TDP1-FLAG overexpression plasmid and detected TOP1-DPCs. TDP1 upregulation led to a decrease in the levels of CPT-induced TOP1-DPCs in comparison with cells transfected with empty vector (EV) (Fig. 3c; Supplementary Fig. 3b, c), consistent with the role of TDP1 in resolving TOP1-DPCs. However, in the presence of BTZ or PARGi, TDP1 upregulation failed to remove TOP1-DPCs. This experiment suggests that although PARylation recruits TDP1 to the DPC sites, dePARylation and degradation of TOP1-DPCs are required for TDP1 to gain access to the phosphodiester bond of the DPCs for their hydrolysis.

To test the activity of TDP1 against PARylated TOP1-DPC in biochemical assays, we generated a TOP1cc using recombinant human TOP1 and a 56 nt DNA suicidal substrate labeled with ^32^P at its 5′-end (Fig. 3d). After PARylation by recombinant human PARP1 with or without NAD^+^, the PARylated and unmodified TOP1ccs were incubated with recombinant human TDP1. We found that, when incubating the DNA substrate (a) with 500 nM TDP1 (the highest concentration used for the assay), TDP1 exhibited 3′-nucleosidase activity toward the substrate by cleaving its 3′-guanosine, resulting in a 55 nt product (b) (Fig. 3e, f). By incubating TDP1 and the unmodified TOP1cc (c), we found that TDP1 was able to hydrolyze the 3′-phosphotyrosyl linkage at high concentrations and released the 53nt product (d) (Fig. 3e, f) that was otherwise covalently attached to TOP1 and retained in the well of the PAGE. In agreement with previous studies^35^, denaturing the TOP1cc by heat facilitated the activity of TDP1 toward the DPC presumably by exposing the otherwise occluded tyrosine-DNA bond^35,36^. PARylation of the TOP1cc (e), had no significant effect on TDP1 activity, although it exhibited slight resistance to TDP1 at physiological (the lowest) concentration of TDP1, suggesting that TDP1 is recruited to TOP1-DPCs by PARylation but does not act efficiently on the DPCs until they are unfolded or debulked^3^.

### PARG is required for the proteasomal degradation of CPT-induced TOP1-DPC

Based on our findings that PARylation enhanced the recruitment of TDP1 to PARylated TOP1-DPCs^19,20^ but blocked the activity of TDP1 against cellular TOP1-DPCs, and prevented the repair of TOP1-DPCs, we hypothesized that PARylation of TOP1-DPCs, if not reversed promptly, may block the activity of the 26S proteasome toward the TOP-DPCs thereby thwarting their degradation and repair.

**Fig. 4:**
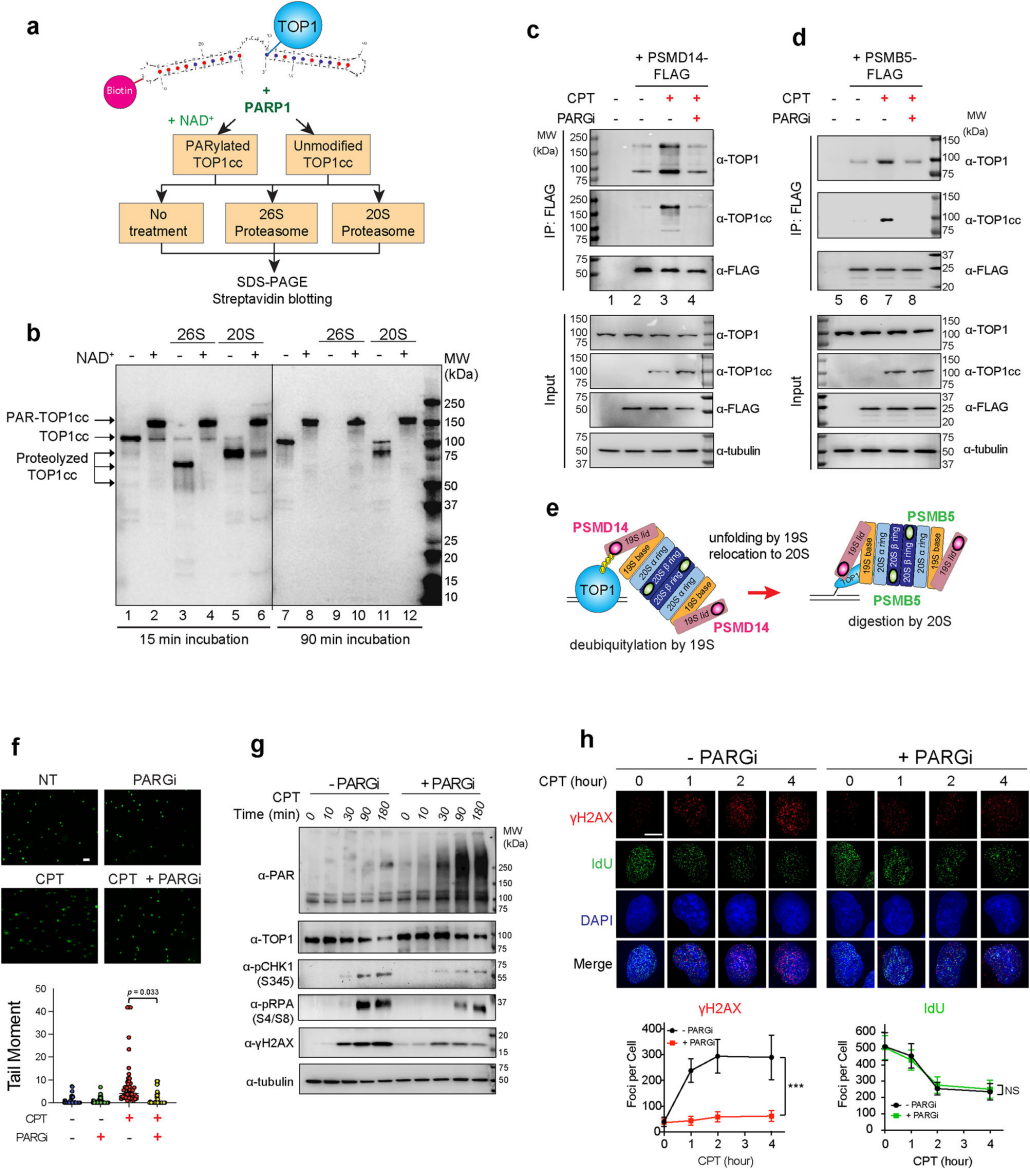
DePARylation of TOP1-DPCs is required for their proteasomal degradation. **a**. Scheme for biotinylated TOP1cc preparation, PARylation, and proteasome digestion assays. **b** TOP1cc proteasomal digestion assay showing that unmodified TOP1ccs (without NAD^+^) are fully degraded by the 26S proteasome and partially degraded by the 20S proteasome after 45 min incubation whereas PARylated TOP1ccs (+ NAD^+^) are refractory to proteasomal degradation. **c** Inhibiting PARG blocked the interactions between TOP1-DPC and the proteasome subunit PSMD14. Following transfection of PSMD14-FLAG expression plasmid, HEK293 cells were treated with the indicated drugs (1 h, 10 µM PARGi pre-treatment followed by 30 min co-treatment with 20 µM CPT) and subjected to IP using an α-FLAG antibody. The immunoprecipitates and input samples were Western blotted with the indicated antibodies. **d** Inhibiting PARG blocked the interactions between TOP1-DPC and the proteasome subunit PSMB5. Following transfection of PSMB5-FLAG expression plasmid, HEK293 cells were treated with the indicated drugs (1 h, 10 µM PARGi pre-treatment followed by 30 min co-treatment with 20 µM CPT) and subjected to IP using an α-FLAG antibody. The immunoprecipitates and input samples were Western blotted with the indicated antibodies. **e**. Model for 26 S proteasome-dependent degradation of TOP1-DPCs. **f** Inhibiting PARG blocked the liberation of TOP1-induced DNA breaks. Upper panels: representative images of alkaline comet assay in HEK293 cells treated with DMSO, CPT (10 µM, 2 h), PARGi (10 µM, 3 h), and CPT + PARGi (pre-treatment with PARGi for 1 h then co-treatment with CPT and PARGi for 2 h). Cells were subjected to alkaline comet assay for detection of DNA breaks. Lower panel: quantitation of tail moments using OpenComet. *n* = 50 biologically independent cells. *P* value was calculated by paired Student’s t-test (two-tailed distribution). The scale bar represents 100 μm. **g** Inhibiting PARG attenuated TOP1-DPC-induced DNA damage response (DDR). HEK293 cells were treated with CPT in absence of the presence of PARGi (pre-treatment for 1 h). Cells were collected at the indicated time points and subjected to non-denaturing lysis and benzonase treatment, followed by Western blotting with the indicated antibodies. **h** Inhibiting PARG reduced CPT-induced γH2AX foci. Upper panels: representative images of IF of γH2AX and IdU foci by iSIM. U2OS cells were synchronized in the S phase by double thymidine block, followed by IdU incorporation and PARGi treatment for 1 h. Cells were then treated with CPT (1 µM) and collected at indicated time points for IF using anti-γH2AX and anti-BrdU antibodies. Lower panels: quantitation of γH2AX foci (left) and quantitation of IdU foci (right). *n* = 10 biologically independent samples. Data are presented as mean values +/− standard deviation (SD). *P* value was calculated by paired Student’s t-test (two-tailed distribution). ***: *p* = 0.000015. NS not significant. The scale bar represents 10 μm.

To test whether dePARylation is required for the proteasomal degradation of TOP1-DPC, we employed the TOP1 suicidal substrate used for the TDP1 activity assay and performed experiments with this substrate labeled with biotin (Fig. 4a). We first PARylated the TOP1cc in vitro with recombinant PARP1 and NAD^+^, followed by incubation with the 26S proteasome holoenzyme or the 20S proteasome only containing the proteolytic core particle for 15 and 90 min (Fig. 4a). After those incubations, samples were subjected to Western blotting, and the TOP1cc and TOP1-polypeptide-DNA crosslinks (TOP1-DpC)^3^ were detected with streptavidin by virtue of the covalent attachment of the DNA to TOP1 catalytic tyrosyl residue (Fig. 4a). The TOP1cc was PARylated by PARP1 and appeared as a 150−160 kDa band (Fig. 4b, lane 2: PAR-TOP1cc). After incubation with the 26S proteasome for 15 min, the unmodified TOP1cc was partially proteolyzed (Fig. 4b, lane 3) whereas the PARylated TOP1cc was refractory to the 26S proteasome (Fig. 4b, lane 4). Incubation with the 20S proteasome for 15 min digested the unmodified TOP1cc to an ~80 kDa product (Fig. 4b, lane 5) but did not process the PARylated TOP1cc (Fig. 4b, lane 6). After 90 min incubation with the 26S proteasome, the unmodified TOP1cc was fully digested (Fig. 4b, lane 9) whereas the PARylated TOP1cc remained intact Fig. 4b, (lane 10). After 90 min incubation, the 20S proteasome was unable to fully digest the unmodified TOP1cc, and an ~80 kDa partially degraded TOP1cc was produced by 20S proteasomal degradation (Fig. 4b, lane 11; Supplementary Fig. 4a). This is presumably because the 20S proteasome core particle cannot unfold proteins due to the lack of the AAA^+^ ATPases that are located in the 19S regulatory particle of the 26S proteasome complex for unfolding substrates^37^, a necessary step for translocating substrates to the tunnel of the 20S core particle (Fig. 4e). Like the 26S proteasome, the 20S proteasome was unable to digest the PARylated TOP1cc even after 90 min incubation (Fig. 4b, lane 12). These experiments show that PARylation of TOP1-DPC prevents the proteasomal degradation of TOP1-DPCs.

Of note, the observation that the 20S proteasome degraded TOP1cc to an 80 kDa product suggests that TOP1-DPCs are degraded starting from the amino-terminus of TOP1. Indeed, because the covalent attachment of the human TOP1 tyrosyl residue to DNA is at amino acid residue 723 (42 amino acid residues from the carboxyl terminus of TOP1), if TOP1-DPCs entered the proteasome core particle from their carboxyl-terminus and were digested from that end, the 80 kDa product would not contain the carboxyl-terminal catalytic tyrosine residue covalent linked to the biotinylated substrate used to detect the TOP1-DpC. This interpretation is consistent with the fact that the amino-terminus of TOP1 bears an unstructured domain^38^, which has been proposed to enhance the susceptibility to the proteasome and to determine the direction of entry of TOP1-DPCs into the proteasome^39^.

To further interrogate whether TOP1 PARylation abolishes the proteasomal degradation of TOP1-DPCs in vivo, we overexpressed PSMD14, an essential component of the 19S regulatory particle subunit that deubiquitylates substrate proteins^37^ (Fig. 4e) in HEK29 cells. FLAG-IP pull-down of PSMD14-FLAG with subsequent probing with anti-TOP1 antibody showed that 30 min CPT (20 μM) treatment enhanced the interaction between TOP1 and PSMD14 (Fig. 4c, lane 3, top panel). Furthermore, the PSMD14-TOP1-DPC interaction induced by CPT was detected by probing the samples using an anti-TOP1cc antibody (Fig. 4c; 2nd panel from the top). Using these two antibodies, we not only observed the band corresponding to unmodified TOP1 and TOP1-DPCs but also multiple bands above the size of unmodified TOP1 and TOP1-DPCs, consistent with the fact that ubiquitylated TOP1 species are bound to PSMD14^16^. PARG inhibition mitigated the interactions of PSMD14 with TOP1 and TOP1-DPCs (Fig. 4c, lane 4). This result is in agreement with the biochemical experiments showing that persistent PARylation prevents the 26S proteasome from targeting TOP1-DPC.

To examine the impact of TOP1-DPC PARylation on the proteasome core particle activity, we transfected a plasmid expressing PSMB5-FLAG, a 20S proteasome core particle subunit that proteolyzes substrate proteins through its chymotrypsin-like activity^37^. FLAG-IP showed that CPT enhanced the interaction between TOP1 and PSMB5 (Fig. 4d; top panel, lane 3) and that PSMB5 targeted TOP1-DPCs upon their formation (Fig. 4d; 2nd top panel, lane 3). Yet, we did not observe bands above the size of unmodified TOP1 and TOP1-DPCs, suggesting that TOP1-DPCs are deubiquitylated by PSMD14 before their translocation to the 20S core particle where they are proteolyzed (Fig. 4e). Consistent with the results of PSMD14 FLAG-IP, PARG inhibition diminished the interaction of PSMB5 with TOP1 and TOP1-DPCs (Fig. 4d, lane 4). Together the results of the biochemical and cellular experiments demonstrate that modification of the TOP1-DPCs with the bulky PAR polymers blocks their recognition and processing by the proteasome.

### PARG is required for activation of DNA damage responses (DDR) against TOP1-DPCs

Prior studies have reported that inhibiting the proteasome blocks DDR activation presumably because the proteasome is required for liberation/exposure of the concealed breaks within TOP1-DPCs^14–16,21^.

Because our experiments showed that PARylation blocked the proteasomal debulking of TOP1-DPCs, we tested whether inhibiting PARG also prevented DDR activation in response to TOP1-DPCs. First, we performed alkaline and neutral comet assays and observed fewer DNA breaks in HEK293 cells co-treated with CPT and PARGi than in cells only treated with CPT (Fig. 4f, Supplementary Fig. 4b). In accordance with this finding, Western blotting in HEK293 cells showed that PARGi alleviated CPT-induced downregulation of TOP1 and attenuated the activation of CHK1, phosphorylation of RPA, and the induction of γH2AX in response to CPT (Fig. 4g). Furthermore, by performing instant structured illumination microscopy (iSIM) for IF of γH2AX in U2OS cells synchronized in the S phase, we observed a significant reduction in CPT-induced γH2AX, which is indicative of TOP1-induced single-end DSBs (seDSBs) upon treatment with PARGi (Fig. 4h). These results demonstrate that PARG, by dePARylating TOP1-DPCS, enables the proteasomal degradation and unmasking of the TOP1-DPCs hence the activation of DDR.

As inhibiting PARP1 also led to an elevation in CPT-induced TOP1-DPCs (see Supplementary Fig. 2i), we next sought to explore if PARP1-dependent PARylation limits the removal of TOP1-DPCs. We performed modified RADAR assays in HEK293 cells treated with or without PARPi and observed that, after 1 h CPT treatment, PARPi-treated cells exhibited higher levels of TOP1-DPCs than cells without PARPi treatment (Supplementary Fig. 4c, upper panels). Yet, after 2 h, the PARPi-treated cells removed the TOP1-DPCs as efficiently as cells without PARPi, demonstrating that inhibiting PARPi delays but does not block TOP1-DPC removal. This finding suggests that the PARPi-induced delay in TOP1-DPC removal is likely due to defective TDP1 recruitment and that the delayed TOP1-DPC removal, which is not affected by PARPi, likely results from alternative endonuclease pathways^3,13^. Consistent with this possibility, the levels of γH2AX, pRPA32, and pCHK1 in PARPi-treated cells were lower than in control cells 1 h after CPT treatment (Supplementary Fig. 4c, lower panels), suggesting that PARPi-induced defective processing of TOP1-DPCs suppressed the liberation of TOP1-linked breaks, therefore, the activation of DDR at early times. Yet, after 4 h, TOP1-DPCs in both PARPi-treated and control cells were equally removed, leading to higher levels of DDR markers in PARPi-treated cells likely due to the defects in SSB and DSB repair when PARP1 is inhibited^40^.

### PARylation of TOP1-DPCs trigger their deubiquitylation by USP7

**Fig. 5:**
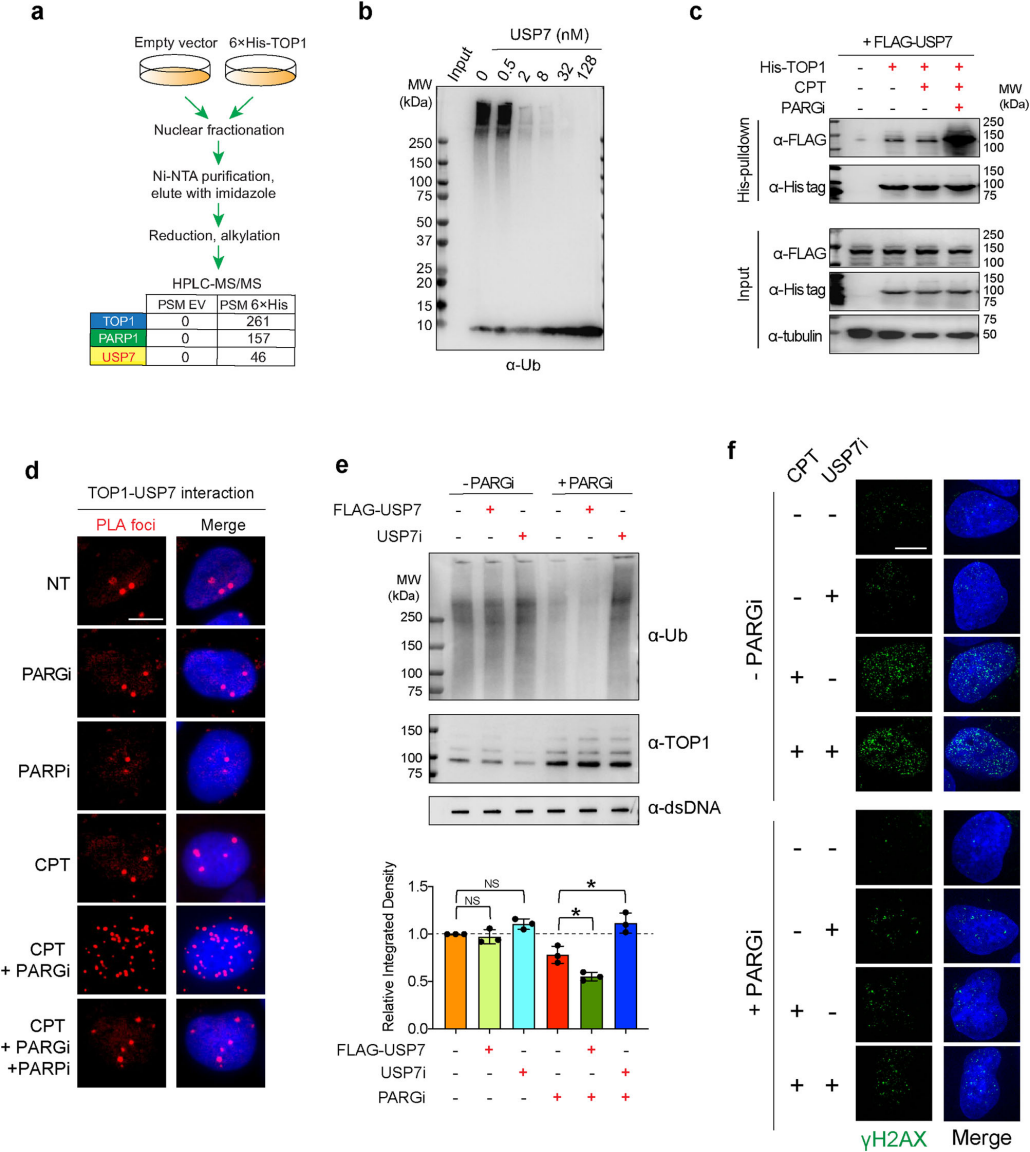
Inhibiting PARG triggers TOP1-DPC deubiquitylation by USP7. **a** His-tag pulldown-HPLC-MS/MS showing that His-TOP1 interacted USP7 under unperturbed condition. After transfection of 6×His-tagged TOP1 expression plasmid or empty vector control (pTrex), HCT116 cells were subjected to His-tag pulldown using Ni-NTA agarose, followed by HPLC-MS. **b** In vitro assay showing that USP7 reversed TOP1 ubiquitylation. Recombinant TOP1 was subjected to ubiquitylation with ubiquitin, Ube1 (E1), Ubc5Hα (E2), and RNF4 (E3) for 30 min, followed by termination with EDTA and incubation with increasing concentrations of recombinant USP7 for another 30 min. Samples were Western blotted with α-Ub antibody. **c** His-tag pulldown assay showing that PARGi enhanced TOP1-USP7 interaction. Following transfection of 6×His-tagged TOP1 expression plasmid and FLAG-USP7 expression plasmid, HEK293 cells were treated as indicated. His-tag pulldown was performed with Ni-NTA agarose under native conditions. Western blotting was performed with the indicated antibodies. **d** PLA assay showing TOP1-USP7 interaction in PARGi-treated cells. Following transfection of 6×His-tagged TOP1 expression plasmid and FLAG-USP7 expression plasmid, HEK293 cells were treated as indicated. PLA assays were performed using rabbit α-His-tag antibody and mouse α-FLAG tag antibody. The scale bar represents 10 μm. **e** Inhibiting USP7 restored TOP1-DPC ubiquitylation in the presence but not in the absence of PARGi. Upper panel**:** HEK293 cells were treated as indicated: CPT (20 µM, 1 h), CPT + FLAG-USP7 transfection, CPT + USP7i (10 µM, 1 h pre-treatment), CPT + PARGi (10 µM, 1 h pre-treatment), CPT + PARGi + FLAG-USP7 transfection, CPT + PARGi + USP7i. Following treatments, cells were subjected to the modified RADAR assay for detection of TOP1-DPCs and their ubiquitylation using α-TOP1 and α-Ub antibodies. Lower panel**:** densitometric quantitation of ubiquitylated TOP1-DPC signals generated from triplicate experiments including representative blots shown in (**c**) using ImageJ. *n* = 3 independent experiments. Data are presented as mean values +/− standard deviation (SD). *P* value was calculated by paired Student’s t-test (two-tailed distribution). *: *p* = . NS not significant. **f** Inhibiting USP7 did not impact the induction of γH2AX upon exposure to CPT. U2OS cells were synchronized in the S phase by double thymidine and treated with CPT (1 µM) in the absence of presence of USP7i (10 µM, 1 h pre-treatment) and collected for IF by iSIM using an anti-γH2AX antibody. The scale bar represents 10 μm.

Our observation that PARylation not only led to an accumulation of TOP1-DPCs but also reduced their ubiquitylation 2 h after CPT treatment (see Fig. 2e) suggested that PAR-stabilized TOP1-DPCs may undergo deubiquitylation. Indeed, His-pulldown-HPLC-MS analysis identified several deubiquitylating enzymes (DUBs) enriched in the His-TOP1 expressing cells but not in control cells transfected with empty vector (EV) (Fig. 5a and Supplementary Data 1). Among the identified DUBs, the ubiquitin-specific protease USP7 showed the highest peptide spectrum match score (PSM).

To determine whether USP7 acts as a DUB for TOP1, we carried out biochemical deubiquitylation assays with recombinant human USP7. We first ubiquitylated recombinant TOP1 with ubiquitin E1, E2, and RNF4, a recently established ubiquitin E3 ligase for TOP1-DPCs^16^. We next stopped the reaction by adding excess EDTA and incubated the ubiquitylated TOP1 with increasing concentrations of His-tagged USP7. USP7 reduced the levels of poly-ubiquitylated TOP1 species in a dose-dependent manner (Fig. 5b), confirming the deubiquitylating activity of USP7 against TOP1. To confirm that TOP1 and USP7 interacted in vivo, we performed His-tag pulldown assays in HEK293 cells co-transfected with FLAG-USP7 and His-TOP1. A strong interaction was observed between the two proteins in the presence of CPT and PARGi (Fig. 5c). By conducting PLA assays, we substantiated the binding of USP7 to PARylated TOP1-DPCs and found that the binding was abrogated by PARPi (Fig. 5d; Supplementary Fig. 5a). Together, these experiments indicate that USP7 targets TOP1-DPCs in a PAR-dependent manner.

We next tested the role of USP7 for TOP1-DPCs by treating cells with the USP7 inhibitor P5091^41^. Using the modified RADAR assay, we found that USP7 inhibition increased TOP1-DPC ubiquitylation after CPT treatment in the presence of PARGi (Fig. 5e; Supplementary Fig. 5b), suggesting that hyper-PARylation of TOP1-DPC triggers USP7-mediated deubiquitylation of the DPCs. To extend these findings, we downregulated USP7 by siRNA in HEK293 cells and found that USP7 knockdown also enhanced the ubiquitylation of TOP1-DPCs induced by co-treatment with CPT and PARGi (Supplementary Fig. 5c, d). Neither inhibiting nor downregulating USP7 affected the levels of total TOP1-DPCs in cells treated with PARG inhibitor (Fig. 5e and Supplementary Fig. 5d). In addition, treatment with USP7i did not alter the levels of TOP1-DPC-induced γH2AX foci (Fig. 5f and Supplementary Fig. 5e). These experiments indicate that USP7 is not directly involved in the repair of TOP1-DPCs. Together, these results suggest that PARylation of TOP1-DPCs exerts a dominant effect over their ubiquitylation by blocking their proteasomal processing and by inducing their deubiquitylation by USP7.

## Discussion

**Fig. 6:**
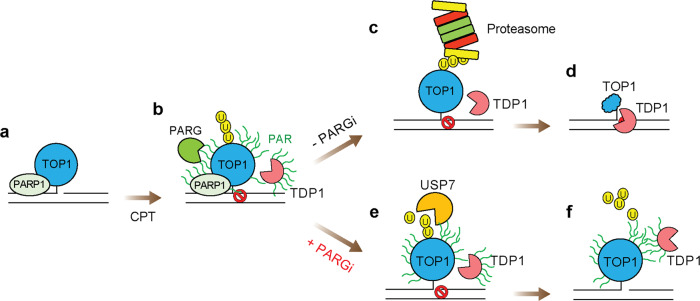
Model for the role of PARG in the repair of TOP1-DPCs. **a** PARP1 and TOP1 form cellular protein complexes (present study and refs. ^22,34^). **b** TOP1-DPC trapped by CPT is rapidly modified with PAR by PARP1 and with ubiquitin (by RNF4 and potentially other E3 ligases, not shown). The PARylation recruits TDP1, PARG, and USP7 to the TOP1-DPC. **c** TOP1-DPC PARylation is readily and rapidly reversed by PARG, enabling the 26S proteasome to target the ubiquitylated TOP1-DPC for degradation. **d** TDP1 hydrolyzes the TOP1 peptide to expose the DNA ends for repair. **e** In the presence of PARGi, TOP1-DPC dePARylation is blocked and the persistent PAR polymers on TOP1-DPC obstruct the proteasome hence stabilize TOP1-DPC. **f** The stabilization of TOP1-DPC triggers USP7 to deubiquitylate the DPC to recycle the ubiquitin molecules.

In this report, we show that PARylation blocks the proteasome-dependent proteolysis of TOP1-DPCs and activates their deubiquitylation. Our finding that TOP1-DPC PARylation is undetectable in the absence of PARG inhibitor, suggests that PARylation needs to be transient for the normal repair of TOP1-DPCs. Prompt dePARylation appears to be required for the proteasomal degradation of TOP1-DPCs, as evidence by our observation that PARylated TOP1-DPCs are refractory to degradation by 26S and 20S proteasomes, and that cells treated with PARG inhibitor accumulat and fail to remove TOP1-DPCs. Although PARP1-mediated PARylation recruits TDP1 to TOP1-DPCs^19,33^, it prevents the activity of TDP1 toward TOP1-DPCs in vivo likely because the bulky PAR polymers block the proteolysis hence the exposure of the phosphotyrosyl bond. We, therefore, hypothesize that PARylation of TOP1-DPCs is rapidly reversed by PARG following the co-localization of TDP1 with TOP1-DPCs to enable the proteasomal degradation of the bulky TOP1 adducts. TDP1, which remains actively associated with the repair complex would then act to hydrolyze the TOP1-DpC phosphotyrosyl linkage (Fig. 6).

The PARylation-dependent TDP1 pathway and the ubiquitylation-dependent proteasome pathway appear to be epistatic for TOP1-DPC repair^3,35^. Unlike SUMO-2/3 modification, which reportedly alters the conformation of TOP2-DPCs to allow TDP2 to resolve the DPCs without proteolysis in vitro^42^, we find that PARylation does not impact either TOP1 cleavage or the activity of TDP1 toward TOP1-DPCs in vitro. This suggests that PARylation of TOP1-DPCs may solely act as a signaling mechanism for recruiting TDP1, and that TDP1 resolves the DPCs once they are debulked by the proteasome.

In contrast to ubiquitylation that modifies TOP1-DPCs in part in replication/transcription-associated manners^14,15^, we found that TOP1-DPC PARylation occurs independently of replication or transcription. These observations suggest that the ubiquitin-mediated proteasomal repair of TOP1-DPCs is not required for their PARylation hence TDP1 recruitment, and that ubiquitylation and PARylation of TOP1-DPCs are triggered by different mechanisms to elicit the proteasome and TDP1, respectively, to orchestrate the resolution of TOP1-DPCs.

Implicit in this model is that PARylation may play two different roles for the repair of TOP1-DPCs: on one hand, PARylation of the DPC serves as a signal for the recruitment of TDP1 (Fig. 6b); on the other hand, PARylation “sugarcoats” the TOP1-DPC thereby shields it against the proteasome until TDP1 is fully recruited. Indeed, by HPLC-MS analyses, we identified several proteasomal subunits (but not TDP1) interacting with TOP1 even in the absence of CPT (Supplementary Data 1), suggesting that the proteasome system acts as a “guardian” that readily protects the genome from TOP1-DPCs. It can be imagined that if TDP1 is signaled through peptide modification (e.g., ubiquitylation) instead of sugar modification, it may result in premature degradation by the proteasome, leading to loss of the signal and failure to recruit TDP1.

It was initially reported that PARylation of TDP1 by PARP1 translocates the phosphodierase to TOP1-DPCs for excision^19^. Our findings imply a model wherein upon sensing the stabilization (trapping) of TOP1ccs, PARP1 catalyzes PARylation of both the DPCs and TDP1: the former evokes TDP1 as a signal and the latter helps TDP1 localize to the DPCs as a shuttle (Fig. 6b). Interestingly, PARylation was also found to stabilize TDP1^19^, raising the possibility that TDP1 PARylation blocks its proteasomal degradation. Yet, since TDP1 is activated by deubiquitylation upon TOP1-DPC induction^43^, it is unlikely that TDP1, once it is recruited and dePARylated, becomes a target of the proteasome, which would lead to failure to resolve the DPCs.

Akin to PARylation, SUMOylation has been shown to modify both TOP1-DPC and TDP1^44^. TOP1-DPC SUMOylation has recently been found to trigger RNF4-mediated ubiquitylation for proteasomal degradation^16^. TDP1 SUMOylation is required for its accumulation at the sites of TOP1-mediated SSB^44^. The potential interplay between these two modifications for TOP1-DPC repair, therefore, warrants further investigations.

In the present study, we also identified a previously unknown deubiquitylation pathway for TOP1-DPCs by USP7, which was uncovered upon blockade of the dePARylation of TOP1-DPCs. This raises the possibility that USP7 induces ubiquitin recycling of the long-lived PARylated TOP1-DPCs (Fig. 6e). USP7 has also been identified as a DUB that reverses RNF4-mediated polyubiquitylation^45,46^. Given the role of RNF4 for TOP1-DPC ubiquitylation and degradation^16^, it can be conjectured that USP7 is recruited by persistent PARylation to reverse RNF4-induced ubiquitylation of TOP1-DPCs. As USP7 appears not to be important for the repair of TOP1-DPCs, we believe that USP7 is recruited as a mechanism to recycle ubiquitin molecules attached to the DPCs when the proteasomal DUB subunit is blocked by the PARylation.

It is well-established that genotoxic agents induce poly(ADP-ribosyl)ation and mono(ADP-ribosyl)ation of a broad range of chromatin-binding proteins^47,48^, many of which can be covalently trapped on DNA by crosslinking agents. It can therefore be speculated that the fine-tuned PARylation/dePARylation mechanism investigated here for TOP1-DPCs may be shared by a broader range of DPCs, and that prompt dePARylation is also a prerequisite for the repair of other DPCs by the proteasome as well as other DPC-targeting proteases.

## Methods

### Human cell culture

Human embryonic kidney HEK293 cells, human bone osteosarcoma U2OS cells and human colorectal carcinoma HCT116 cells were cultured in DMEM medium (Life Technologies) supplemented with 10% (v/v) fetal bovine serum, 100 units/ml penicillin, 100 μg streptomycin /ml streptomycin, and 1x GlutaMax in tissue culture dishes at 37 °C in a humidified CO2—regulated (5%) incubator.

### Chemicals

PDD 00017273 (PARGi, Tocris); camptothecin (CPT, Sigma Aldrich); the replication inhibitor aphidicolin (APD, Sigma Aldrich); the transcription inhibitor 5,6-dichloro-1-beta-ribo-furanosyl benzimidazole (DRB, Sigma Aldrich); talazoparib (PARPi, Selleck); P5091 (USP7i, Selleck).

### Antibodies

The following antibodies were used: anti-PAR, mouse monoclonal, Trevigen, 4335-MC-100; anti-PAR (10H), mouse monoclonal, Enzo Life Sciences, ALX-804-220; anti-ubiquitin, mouse monoclonal, Santa Cruz, sc-8017; anti-His tag, mouse monoclonal, Abcam, ab18184; anti-His tag, rabbit monoclonal, Cell signaling, 12698; anti-TOP1, mouse monoclonal, BD Biosciences, 556597; anti-dsDNA, mouse monoclonal, Abcam, ab27156; anti-FLAG, mouse monoclonal, Sigma Aldrich, F1804; anti-FLAG, rabbit polyclonal, Sigma Aldrich, F7425; anti-TDP1, rabbit polyclonal, Bethyl Laboratories, A301-618A; anti-PARP1 (F2), mouse monoclonal, Santa Cruz, sc-8007; anti-PARG, rabbit monoclonal, Cell Signaling, 66564; anti-γH2AX, rabbit polyclonal, Cell Signaling, 66564; anti-BrdU, mouse monoclonal, Abcam, ab8152; anti-USP7, rabbit polyclonal, Bethyl Lab, A300-033A; Rabbit TrueBlot: anti-rabbit IgG HRP, Rockland, 18-8816-31; Mouse TrueBlot ULTRA: anti-mouse Ig HRP, Rockland, 18-8817-31; anti-Rabbit IgG, Alexa Fluor 568, Thermo Fisher, A-11011; anti-Mouse IgG, Alexa Fluor 488, Thermo Fisher, A-28175.

### Recombinant proteins

Human recombinant Top1 baculovirus-infected insect cells in media were centrifuged at 2,000 × *g* and the pellet was resuspended in ice-cold lysis buffer (25 mM Tris pH 7.5, 100 mM NaCl, 5 mM EDTA, 0.5% NP40, 4 mM β-mercaptoethanol, and 10 mM PMSF) and then incubated on ice for 10 min. The sample was then centrifuged at 6,000 × *g* for 20 min and the supernatant was then dialyzed against 25 mM Tris pH 7.5, and 4 mM β-mercaptoethanol to remove the EDTA. TOP1 was purified on a HiTrap Q HP column (Amersham Biosciences, Uppsala, Sweden) in buffer A (25 mM Tris pH 7.5, 5 mM β-mercaptoethanol, 5 mM EDTA) and eluted with buffer B (buffer A with 1 M KCl). The TOP1 peak fraction was dialyzed against buffer A, made 50% glycerol, and stored at −20 °C^49^. Human recombinant PARP1 was purified from *E. coli* as described^50^. Human recombinant USP7 was purchased from R&D Systems (cat # E-519). Human recombinant ubiquitin was purchased from Boston Biochem (cat # U-100H). Human recombinant Ubiquitin Activating Enzyme (UBE1) was purchased from Boston Biochem (cat # E-305). Human recombinant UbcH5a/UBE2D1 was purchased from Boston Biochem (cat # E2-616). Human recombinant RNF4 was purchased from R&D Systems (cat # E3-210-050).

### Expression plasmids

TOP1-HaloTag expression plasmid was constructed via Golden GATEway cloning^28^. A 6×His-TOP1 fragment with 5′ attB1 site and 3′ BbsI digestion site was generated by PCR using pTrex-6×His-TOP1 as a template. A TEV site/HaloTag fragment with 5′ BbsI digestion site and 3′ attB2 site was generated using pHTC HaloTag^®^ CMV-neo vector (Promega) as a template. The two fragments were ligated by BbsI digestion and T4 ligase to construct a scarless [6xHis-TOP1]:[TEV site/HaloTag] fragment. The BP reaction was performed using the [6xHis-TOP1]:[TEV site/HaloTag] fragment and a donor vector pDown-BsaI-ccdB-Cm-BsaI (VectorBuilder) to generate entry clone pDown-[6xHis-TOP1]:[TEV site/HaloTag]. The LR reaction was conducted for the combination of pUP-CMV (entry clone #1, VectorBuilder) and pDown-[6xHis-TOP1]:[TEV site/HaloTag] (entry clone #2) with the pUC19-CMV>Neo backbone (destination vector) to construct the expression plasmid. Following *E. coli* transformation, positive clones were obtained through Ampicillin screening and verified by PCR, sequencing, and Western blotting. A full list of primers used for generation and sequencing of pUC19-CMV>Neo His6-TOP1-HaloTag is available in Supplementary Table 1.

In addition, the following expression plasmids were used: pT-REx His6-TOP1;^16^ pCMV PARP1-3xFlag, Addgene, 111575; pCMV TDP1-FLAG, OriGene, RC214927; pCI-Neo Flag-USP7, Addgene, 16655. Forty-eight hours of transfection was performed using lipofectamine™ 3000 transfection reagent (Invitrogen) following the manufacturer’s instructions.

### Small-interfering RNA (siRNA)

The following siRNAs were used: control siRNA, Dharmacon, D-001206-13-05; TOP1 siRNA, Dharmacon, M-005278-00-0005; PARG siRNA, Dharmacon, L-011488-00-0005; USP7 siRNA, Dharmacon, L-006097-00-0005. Seventy-two hours of transfection was performed using lipofectamine™ RNAiMAX transfection reagent (Invitrogen) following the manufacturer’s instructions.

### Single-molecule fluorescence microscopy

Single-molecule imaging experiments were conducted on a custom-built Nikon Ti microscope. The microscope is equipped with a 100 × oil-immersion objective lens (N.A. = 1.49), a multi-band dichroic (405/488/561/633 BrightLine quad-band bandpass filter, Semrock, USA) and a piezo Z-stage (ASI, USA), a filter wheel (Sutter Instrument, USA) and a stage top incubator (Tokai Hit, Japan). The lasers were focused on the back pupil plane of the objective to generate wide-field illumination. Nikon N-STORM module was used to steer the incidence angle of the laser for generating inclined illumination. The emission was collected by the same objective passing through an emission filter (617/73, Semrock) in front of the sCMOS camera (Prime 95B, Teledyne Photometrics). The microscope, lasers, and the camera were controlled through NIS-Elements (Nikon, USA).

### Single-molecule tracking and analysis

Single-molecule tracking was performed with custom-written MATLAB software (http://site.physics.georgetown.edu/matlab/) based on available tracking algorithms^51^. The MATLAB scripts adapted from IDL Particle Tracking were used to localize and track single molecules. The positions of the diffraction-limited spots in the trajectories were determined with a 2D Gaussian fit.

For jump distance analysis, the probability that a particle located at position *r* at time *t* in two dimension, will be found at position *r*′ at time *t* + tau is given by^52^

1 $$\varphi (r,t)=\left(\frac{1}{4\pi Dt}\right)\exp \left(-{r}^{2}/4Dt\right)$$

Where *D* is the diffusion constant.

In the case of 2D diffusion, the displacement probability is obtained through integrating the above equation over the circular shell of width (d*r*)

2 $$p(r,t){{{{{\mathrm{d}}}}}}r={{{{{\mathrm{d}}}}}}r{\int }_{0}^{2\pi }r\varphi (r,t){{{{{\mathrm{d}}}}}}\theta =\frac{2\pi r{{{{{\mathrm{d}}}}}}r}{4\pi Dt}\exp \left(-\frac{{r}^{2}}{4Dt}\right)$$

Experimentally, this probability distribution can be approximated by counting the jump distances within respective intervals (*r*, *r* + d*r*) traveled by a single molecule during a given time.

Mean square displacements (MSDs) were calculated from *xy* positions as previously described^53^. The tracks were computed and plotted with @msdanalyzer script^54^. Quantitation of TOP1-HaloTag single molecules was performed using ThunderSTORM, an ImageJ plug-in.

### Western blotting

Cellular proteins were detected by lysing cells with RIPA buffer (150 mM NaCl, 1% NP-40, 0.5% sodium deoxycholate, 0.1% SDS, 50 mM Tris pH 7.5, 1 mM DTT and protease inhibitor cocktail), followed by sonication and centrifugation. The supernatant was collected and boiled for 10 mins, analyzed by SDS-PAGE, and immunoblotted with various antibodies as indicated. TOP1 downregulation was monitored using the alkaline lysis method. Following treatment, cells were washed with Dulbecco’s modified Eagle medium and incubated at 37 °C in a CO2 incubator for 30 min then lysed with 100 μl of an alkaline lysis buffer (200 mM NaOH and 2 mM EDTA). Alkaline lysates were neutralized by the addition of 100 μl of 1 M HEPES buffer (pH 7.3), followed by mixing with 10 μl 100 mM CaCl_2_, 1 μl, 2 M dithiothreitol (DTT), and 2 μl 100× protease inhibitor cocktail (Thermo Fisher Scientific) and 200 units of micrococcal nuclease (Thermo Fisher Scientific; 100 units/μl). The resulting mixtures were incubated on ice for 1 h after which 70 μl of 4× Laemmli buffer was added to each sample. The lysates were boiled for 10 min, analyzed by SDS-polyacrylamide gel electrophoresis (SDS-PAGE), and immunoblotted with anti-TOP1 antibody as indicated.

### His-pulldown assay

1 million Human cells were washed with 1 × PBS and incubated with 220 μl IP lysis buffer (5 mM Tris-HCl pH 7.4, 150 mM NaCl, 1 mM EDTA, 1% NP-40, 0.2% Triton X-100, 5% glycerol, 1 mM DTT, 20 mM N-ethylmaleimide (Sigma Aldrich) and protease inhibitor cocktail) on a shaker for 15 min at 4 °C, followed by sonication and centrifugation. The supernatant was collected and treated with 1 μl benzonase (250 units/μl, EMD Millipore) for 1 h. An aliquot (20 μl) of the lysate of each treatment group was saved as input. The rest of the lysates was divided into two groups: native pull-down and denaturing pull-down. For native pull-down, lysates were resuspended in 900 μl IP lysis buffer containing 10 mM imidazole and 100 μl equilibrated Ni-NTA-agarose and rotated overnight at 4 °C. The resin was spun down and washed with TI buffer two times (25 mM Tris HCL, 20 mM imidazole, pH 6.8), followed by resuspension in 2 × Laemmli buffer for SDS-PAGE and immunoblotting with various antibodies as indicated. For denaturing pull-down, lysates were resuspended in 900 μl Buffer A (6 M guanidine-HCL, 0.1 M Na_2_HPO_4_/NaH_2_PO_4_ pH 6.5, 10 mM imidazole pH 8.0) containing 100 μl equilibrated Ni-NTA-agarose and rotated overnight at 4 °C. The samples were washed, resuspended in 2 × Laemmli buffer for SDS-PAGE and immunoblotted.

### Proximity ligation assay (PLA)

Duolink PLA fluorescence assay (Sigma Aldrich, Cat# DUO92101) was performed following the manufacturer’s instructions. In brief, U2OS cells were seeded on coverslips and treated with CPT for 30 min. After treatment, cells were washed with 1XPBS and fixed for 15 min at 4 °C in 4% paraformaldehyde in PBS and permeabilized with 0.25% Triton X-100 in PBS for 15 min at 4 °C. The coverslips were blocked with Duolink blocking solution and incubated with indicated antibodies in the Duolink antibody diluent overnight, followed by incubation with PLUS and MINUS PLA probes, ligation, and amplification. Coverslips were then washed and mounted with using a mounting medium with DAPI. Images were captured on wide-field microscope, processed using ImageJ, and analyzed using Imaris.

### Modification of the RADAR assay for detection of PARylated and ubiquitylated TOP1-DPCs

After CPT treatment, 1 × 10^6^ human cells in 35 mm dish per sample were washed with 1 × PBS and lysed with 600 μl DNAzol (Invitrogen), followed by precipitation with 300 μl 200 proof ethanol. The nucleic acids were collected, washed with 75% ethanol, resuspended in 200 μl TE buffer then heated at 65 °C for 15 min, followed by shearing with sonication (40% output for 10 s pulse and 10 s rest for four times). The samples were centrifuged at 15,000 rpm for 5 min at 4 °C and the supernatant was collected and treated with RNase A (100 μg/ml) for 1 h at 4 °C, followed by the addition of 1/10 volume of 3 M sodium acetate and 2.5 volume of 200 proof ethanol. After 20 min full speed centrifugation, the DNA pellet was recovered and resuspended in 100 μl TE buffer. One microliter of the sample was removed for spectrophotometric measurement of absorbance at 260 nm to quantitate DNA content (NanoDrop). Ten micrograms of DNA from each sample was digested with 50 units of micrococcal nuclease (100 units/μl, Thermo Fisher Scientific) in presence of 5 mM CaCl_2_, followed by gel electrophoresis on 4–15% precast polyacrylamide gel (Bio-Rad) for immunodetection of total TOP1-DPCs, ubiquitylated TOP1-DPCs as well as PARylated TOP1-DPCs using specific antibodies. Due to the extremely low-abundance of PARylated and ubiquitylated TOP1-DPCs, samples were run in parallel gels to detect total, PARylated and ubiquitylated TOP-DPCs separately instead of stripping and reprobing the same membrane for their detection. In addition, 2 μg of each sample was subjected to slot-blot for immunoblotting with anti-dsDNA antibody as a loading control to verify that amounts of DNA were digested with micrococcal nuclease.

### In vivo complex of enzyme (ICE) assay

TOP1-DPCs were isolated and detected by ICE assay^32^. Briefly, HEK293 cells were lysed in sarkosyl solution (1% w/v) then sheared with a 25-gauge 5/8 needle. The lysates were loaded onto CsCl solution (150% w/v) for ultracentrifugation in NVT 65.2 rotor (Beckman coulter) at 15,4893 × *g* for 20 h at 4 °C. The resulting DNA pellets were retrieved and suspended in TE buffer. The samples were quantitated and 2 μg DNA per sample was subjected to slot-blot using indicated antibodies.

### Subcellular fractionation

Subcellular fraction of HEK293 cells was performed using Thermo Scientific^TM^ subcellular protein fractionation kit for cultured cells (cat# 78840) following the manufacturer’s instructions. The cyto/nucleoplasmic fractions and the chromatin fraction were subjected to Western blotting using indicated antibodies.

### Immunofluorescence of PAR on chromatin

After co-treatment with CPT and PARGi, U2OS cells in chamber slides were permeabilized in 0.1% triton X-100 in PBS (PBST) for 1 min on ice, followed by 15 min incubation with 4% paraformaldehyde (PFA) at room temperature Samples were blocked with 1% BSA in PBST for 30 min then incubated with mouse-anti PAR polymer antibody (10H) overnight 4 °C overnight. The next day, samples were incubated with Alexa Fluro 488-conjugated anti-mouse 2nd antibody for 1 h at room temperature and mounted with DAPI containing mounting medium (Vectashield). Images were acquired on Zeiss LSM 880/Airyscan confocal microscope.

### Generation of biotinylated TOP1cc

56-nt DNA oligo (TOP1 suicide substrate) with sequence GTCTGTCCGCT-T(biotin)-TAGCGGACAGACATCATATCTTCAACGTTTACGTTGAAGATATG was purchased from IDT and annealed in 10 mM Tris-HCl, pH7.5, 50 mM NaCl and 1 mM EDTA. The DNA substrate is combined with human TOP1 at an equal ratio in 10 mM Tris-HCl, pH 7.5, 50 mM KCl, 5 mM MgCl_2_ 0.1 mM EDTA, and 15 μg/ml BSA at 4 °C overnight.

### In vitro TOP1cc PARylation

Biotinylated TOP1cc (200 nM) was incubated with 200 nM recombinant PARP1 enzyme in 1X PARylation buffer (50 mM Tris-HCl pH 8.0, 50 mM NaCl, 10 mM MgCl_2_, 2% glycerol, 1 mM DTT). One millimolar NAD^+^ was added to the reaction as indicated. The reactions were incubated at room temperature for 20 min and inactivated by adding talazoparib (1 μM) or by adding SDS sample buffer for Western blotting analysis.

### Proteasome degradation assay

Biotinylated TOP1cc (unmodified and PARylated, 100 nM) was incubated with 20 nM 20S proteasome (activated by 0.035% SDS) in 1× degradation buffer (50 mM Tris-Cl pH 8.0, 5 mM MgCl_2_, 2% glycerol, 1 mM DTT, and 0.2 mg/mL BSA) or with 500 nM 26S proteasome holoenzyme in 1× degradation buffer containing 1 × ATP Regeneration mix (5 mM ATP, 0.03 mg/ml creatine kinase and 16 mM creatine phosphate) at 30 °C. The reaction was quenched with 2× SDS sample buffer and subjected to SDS-PAGE electrophoresis (4−20% Tris-Glycine-SDS gradient gel, Bio-Rad). Samples were probed with streptavidin and imaged by ChemiDoc Imaging System (Bio-Rad) using DyLight 680 application.

### FLAG immunoprecipitation (IP)

Cells were washed with 1 × PBS and lysed in 200 μl IP lysis buffer (5 mM Tris-HCl pH 7.4, 150 mM NaCl, 1 mM EDTA, 1% NP-40, 0.2% Triton X-100, 5% glycerol, 1 mM DTT, 20 mM N-ethylmaleimide (Sigma Aldrich) and protease inhibitor cocktail) on a shaker for 15 min at 4 °C, followed by sonication and centrifugation. The supernatant was collected and treated with 1 μl benzonase (250 units/μl, EMD Millipore) for 1 h. An aliquot (20 μl) of the lysate of each treatment group was saved as input. Lysates were resuspended in 800 μl IP lysis buffer containing 2.5 μl anti-FLAG M2 antibody (Sigma Aldrich) and rotated overnight at 4 °C. Fifty microliters of Protein A/G PLUS-agarose (Santa Cruz Biotechnology) slurry was added and incubated with the lysates for another 4 h. After centrifugation, immunoprecipitates were washed with RIPA buffer two times then resuspended in 2 × Laemmli buffer for SDS-PAGE and immunoblotting with various antibodies as indicated.

### Alkaline and neutral comet assays

The comet assays were performed according to the Trevigen CometAssay™ kit protocol with slight modifications. Cells were pre-treated with PARGi for 1 h, followed by co-treatment with 20 μM CPT for 2 h. Treated cells were trypsinized at 37 °C for 5 min. An equal amount of drug-free medium was then added to quench the trypsin activity. The cells were spun down and resuspended in fresh PBS. The final cell density was approximately 100,000 cells/ml. Fifty microliters of the cell suspension was then mixed with 500 μl of 0.5% low melting point agarose (Invitrogen) (in PBS) at 37 °C. Fifty microliters of the cell/agarose mixture was transferred onto glass slides. The slides were then immersed in prechilled lysis buffer (2.5 M NaCl, 100 mM EDTA, 10 mM Tris, pH 10.0, 1% Triton X-100, and 10% Me_2_SO) for 1 h. For alkaline comet assay, the slides were immersed in alkaline unwinding solution (200 mM NaOH, 1 mM EDTA) for 30 min at room temperature, followed by electrophoresis in 4 °C alkaline electrophoresis solution (300 mM NaOH, 1 mM EDTA) at 1 volt/cm for 30 min. For neutral comet assay, the slides were immersed in 1 × TBE buffer for electrophoresis at 1 volt/cm for 30 min at room temperature. For both alkaline and neutral comet assays, the slides were immersed in 70 % EtOH for 5 min after electrophoresis then incubated with SYBR^®^ Gold for 30 min. The images were visualized under BioTek Cytation 5 cell imaging reader. Statistical analysis was performed by OpenComet, an ImageJ plugin.

### TDP1 activity assay

Oligonucleotide substrate (GTCTGTCCGCTTTAGCGGACAGACATCATATCTTCAACGTTTACGTTGAAGATATG) was labeled on the 5′-end with [γ-32P] ATP and T4 Polynucleotide Kinase for 1 h at 37 °C, the reaction was stopped with EDTA at 70 °C for 10 min, before passing through mini Quick Spin Oligo Columns (Roche). The oligo was annealed after heating at 95 °C for 5 min. The DNA substrate is combined with human TOP1 at an equal ratio in 10 mM Tris-HCl, pH 7.5, 50 mM KCl, 5 mM MgCl2 0.1 mM EDTA, and 15 μg/ml BSA at 4 °C overnight. TOP1cc were then subjected to in vitro PARylation using human PARP1 described above. A serial diluted recombinant human TDP1 enzyme (0, 0.00125, 0.025, and 0.5 µM) was incubated with 1 nM suicide DNA oligo (DNA only, TOP1cc, or PARylated TOP1cc) in a final volume of 10 µL in 1 × LMP 1 reaction buffer (50 mM Tris-HCl, pH 7.5, 80 mM KCl, 2 mM EDTA, 1 mM DTT, 40 µg/mL BSA, 0.01% Tween 20). The reactions were processed at room temperature for 60 min and terminated by adding 10 µL of 2 × stop buffer (99.5% formamide, 10 mM EDTA, 0.01% methylene blue, and 0.01% bromophenol blue) followed by heat inactivation at 95 °C for 10 min. A 20% DNA sequencing gel was used to load the samples and exposed to a PhosphorImager screen for further analysis by Typhoon FLA 9500 (GE Healthcare).

### Mass spectrum analysis

Samples were either separated by SDS-PAGE for in-gel trypsin digestion or in-solution digested with trypsin following the filter-aided sample preparation (FASP) protocol^55^. Dried peptides were solubilized in 2% acetonitrile, 0.5% acetic acid, 97.5% water for mass spectrometry analysis. They were trapped on a trapping column and separated on a 75 µm × 15 cm, 2 µm Acclaim PepMap reverse phase column (Thermo Scientific) using an UltiMate 3000 RSLCnano HPLC (Thermo Scientific). Peptides were separated at a flow rate of 300 nL/min followed by online analysis by tandem mass spectrometry using a Thermo Orbitrap Fusion mass spectrometer. Peptides were eluted into the mass spectrometer using a linear gradient from 96% mobile phase A (0.1% formic acid in water) to 55% mobile phase B (0.1% formic acid in acetonitrile). Parent full-scan mass spectra were collected in the Orbitrap mass analyzer set to acquire data at 120,000 FWHM resolution; ions were then isolated in the quadrupole mass filter, fragmented within the HCD cell (HCD normalized energy 32%, stepped ± 3%), and the product ions analyzed in the ion trap. Proteome Discoverer 2.2 (Thermo) was used to search the data against human proteins from the UniProt database using SequestHT. The search was limited to tryptic peptides, with maximally two missed cleavages allowed. Cysteine carbamidomethylation was set as a fixed modification, and methionine oxidation was set as a variable modification. Diglycine modification to lysine was set as a variable modification for experiments to identify sites of enzymatic PTMs. The precursor mass tolerance was 10 ppm, and the fragment mass tolerance was 0.6 Da. The Percolator node was used to score and rank peptide matches using a 1% false discovery rate.

### TOP1-mediated DNA cleavage reactions

DNA cleavage reactions were prepared as previously reported with the exception of the DNA substrate^31^. Briefly, a 117 bp DNA oligonucleotide (Integrated DNA Technologies) encompassing the previously identified Top1 cleavage sites in the 161 bp fragment from pBluescript SK(-) phagemid DNA was employed. This 117 bp oligonucleotide contains a single 5′-cytosine overhang, which was 3′-end-labeled by fill-in reaction with [^32^P]dGTP in React 2 buffer (50 mM Tris-HCl, pH 8.0, 100 mM MgCl_2_, and 50 mM NaCl) with 0.5 units of DNA polymerase I (Klenow fragment, New England BioLabs). Unincorporated [^32^P] dGTP was removed using mini Quick Spin DNA columns (Roche, Indianapolis, IN), and the eluate containing the 3′ end-labeled DNA substrate was collected. Approximately 2 nM of radiolabeled DNA substrate was incubated with recombinant Top1 in 20 μL of reaction buffer [10 mM Tris-HCl (pH 7.5), 50 mM KCl, 5 mM MgCl2, 0.1 mM EDTA, and 15 μg/mL BSA] at 25 °C for 20 min in the presence of various concentrations of compounds. The reactions were terminated by adding SDS (0.5% final concentration) followed by the addition of two volumes of loading dye (80% formamide, 10 mM sodium hydroxide, 1 mM sodium EDTA, 0.1% xylene cyanol, and 0.1% bromphenol blue). Aliquots of each reaction mixture were subjected to 20% denaturing PAGE. Gels were dried and visualized by using a phosphoimager and ImageQuant software (Molecular Dynamics). For simplicity, cleavage sites were numbered in the 161 bp fragment^56^.

### Immunofluorescence of γH2AX

Two millimolar of thymidine was added to U2OS cells in chamber slides at 37 °C for 18 h. Thymidine was removed and fresh DMEM medium was added to the slides for incubation for 9 h. Two millimolar of thymidine was added to cells for another 18 h incubation. Cells are released from G1/S boundary by washing with PBS and incubating in a fresh medium. PARGi (10 μM) and IdU (100 μM) were added to cells 1 h before CPT (1 μM) treatment. Cells were collected at indicated time points upon exposure to CPT and washed with PBS, followed by fixation with 4% PFA for 15 min at room temperature. Cells were then incubated with 1.5 M HCl for 30 min at room temperature for DNA denaturation, followed by permeabilization with 0.25% Triton X-100 in PBS (PBST). Cells were blocked with 1% BSA in 0.1% PBST for 30 min, followed by incubation with rabbit anti-γH2AX antibody and mouse anti-BrdU antibody overnight at 4 °C. The next day, Alexa Fluor 568-conjugated anti-rabbit 2nd antibody and Alexa Fluor 488-conjugated anti-mouse antibody were added to the chamber slide for 1 h at room temperature. The slides were incubated with DAPI and mounted using ProLong™ antifade mountant. Images were visualized under a customized instant structured illumination microscope (iSIM). Statistical analysis was performed by ThunderStorm, an ImageJ plugin.

### In vitro deubiquitylation assay

The 10 μl in vitro ubiquitylation assay reactions were set up in 1 × ubiquitin conjugation reaction buffer (R&D systems Cat. # B-70) containing 10 mM Mg^2+^-ATP solution pH 7.0 (R&D systems Cat. # B-20), 100 nM TOP1, 10 μM ubiquitin, 50 nM ubiquitin E1, 0.1 μM UbcH5a, and 0.5 μM RNF4. Reactions were incubated at 37 °C for 30 min and stopped by addition of 20 mM EDTA, followed by aliquoting and incubation with recombinant USP7 of increasing concentrations at 37 °C for another 30 min. The samples were subjected to SDS-PAGE and immunoblotting with an anti-ubiquitin antibody.

### Statistical analyses

Error bars on bar graphs represent standard deviation (SD) and *p*-value was calculated using paired Student’s t-test (two-tailed) for independent samples.

### Reporting summary

Further information on research design is available in the Nature Research Reporting Summary linked to this article.

## Supplementary information

Supplementary Information

Peer Review File

Description of Additional Supplementary Files

Supplementary Data 1

Supplementary Movie 1

Supplementary Movie 2

Supplementary Movie 3

Supplementary Movie 4

Supplementary Movie 5

Supplementary Movie 6

Supplementary Movie 7

Supplementary Movie 8

Supplementary Movie 9

Supplementary Movie 10

Supplementary Movie 11

Supplementary Movie 12

Reporting Summary

## Data Availability

All the datasets for quantitative analyses and uncropped figures including Figs. 2a, 3a, 4c, d, g, 5c, Supplementary Figs. 1b, 2a, f, h−j, 3b, 4a, c, 5b−d are provided as Source Data files. All data within the paper are available from the authors upon request. Source data are provided with this paper.

## Source data

Source Data

## References

[1] Jackson SP, Bartek J (2009). The DNA-damage response in human biology and disease. Nature.

[2] Stingele J, Bellelli R, Boulton SJ (2017). Mechanisms of DNA-protein crosslink repair. Nat. Rev. Mol. Cell Biol..

[3] Sun Y, Saha LK, Saha S, Jo U, Pommier Y (2020). Debulking of topoisomerase DNA-protein crosslinks (TOP-DPC) by the proteasome, non-proteasomal, and non-proteolytic pathways. DNA Repair.

[4] Pommier Y, Sun Y, Huang SN, Nitiss JL (2016). Roles of eukaryotic topoisomerases in transcription, replication and genomic stability. Nat. Rev. Mol. Cell Biol..

[5] Pommier Y (2006). Topoisomerase I inhibitors: camptothecins and beyond. Nat. Rev. Cancer.

[6] Katyal S (2007). TDP1 facilitates chromosomal single-strand break repair in neurons and is neuroprotective in vivo. EMBO J..

[7] McKinnon PJ (2016). Topoisomerases and the regulation of neural function. Nat. Rev. Neurosci..

[8] El-Khamisy SF (2005). Defective DNA single-strand break repair in spinocerebellar ataxia with axonal neuropathy-1. Nature.

[9] Zagnoli-Vieira G, Caldecott KW (2020). Untangling trapped topoisomerases with tyrosyl-DNA phosphodiesterases. DNA Repair.

[10] Sordet O (2009). Ataxia telangiectasia mutated activation by transcription- and topoisomerase I-induced DNA double-strand breaks. EMBO Rep..

[11] Murai J (2014). Rationale for PARP inhibitors in combination therapy with camptothecins or temozolomide based on PARP trapping versus catalytic inhibition. J. Pharmacol. Exp. Ther..

[12] Pommier Y, O’Connor MJ, de Bono J (2016). Laying a trap to kill cancer cells: PARP inhibitors and their mechanisms of action. Sci. Transl. Med..

[13] Sun Y (2020). Excision repair of topoisomerase DNA-protein crosslinks (TOP-DPC). DNA Repair.

[14] Desai SD (2003). Transcription-dependent degradation of topoisomerase I-DNA covalent complexes. Mol. Cell Biol..

[15] Lin CP, Ban Y, Lyu YL, Liu LF (2009). Proteasome-dependent processing of topoisomerase I-DNA adducts into DNA double strand breaks at arrested replication forks. J. Biol. Chem..

[16] Sun, Y. et al. A conserved SUMO pathway repairs topoisomerase DNA-protein cross-links by engaging ubiquitin-mediated proteasomal degradation. *Sci. Adv.* **6**, 46 (2020). 10.1126/sciadv.aba6290 PMC7673754 33188014

[17] Pouliot JJ, Yao KC, Robertson CA, Nash HA (1999). Yeast gene for a Tyr-DNA phosphodiesterase that repairs topoisomerase I complexes. Science.

[18] Pommier Y (2014). Tyrosyl-DNA-phosphodiesterases (TDP1 and TDP2). DNA Repair.

[19] Das BB (2014). PARP1-TDP1 coupling for the repair of topoisomerase I-induced DNA damage. Nucleic Acids Res..

[20] Das SK (2016). Poly(ADP-ribose) polymers regulate DNA topoisomerase I (Top1) nuclear dynamics and camptothecin sensitivity in living cells. Nucleic Acids Res..

[21] Lin CP, Ban Y, Lyu YL, Desai SD, Liu LF (2008). A ubiquitin-proteasome pathway for the repair of topoisomerase I-DNA covalent complexes. J. Biol. Chem..

[22] Yung TM, Sato S, Satoh MS (2004). Poly(ADP-ribosyl)ation as a DNA damage-induced post-translational modification regulating poly(ADP-ribose) polymerase-1-topoisomerase I interaction. J. Biol. Chem..

[23] Bauer PI, Kun E (2000). Binding of Topo I to PARPI-antibody immunocomplex. Int J. Mol. Med..

[24] Malanga M, Althaus FR (2004). Poly(ADP-ribose) reactivates stalled DNA topoisomerase I and Induces DNA strand break resealing. J. Biol. Chem..

[25] Ray Chaudhuri A, Nussenzweig A (2017). The multifaceted roles of PARP1 in DNA repair and chromatin remodelling. Nat. Rev. Mol. Cell Biol..

[26] Park SY, Cheng YC (2005). Poly(ADP-ribose) polymerase-1 could facilitate the religation of topoisomerase I-linked DNA inhibited by camptothecin. Cancer Res..

[27] England CG, Luo H, Cai W (2015). HaloTag technology: a versatile platform for biomedical applications. Bioconjug Chem..

[28] Kirchmaier S, Lust K, Wittbrodt J (2013). Golden GATEway cloning—a combinatorial approach to generate fusion and recombination constructs. PLoS ONE.

[29] Grimm JB (2015). A general method to improve fluorophores for live-cell and single-molecule microscopy. Nat. Methods.

[30] Gravells P, Grant E, Smith KM, James DI, Bryant HE (2017). Specific killing of DNA damage-response deficient cells with inhibitors of poly(ADP-ribose) glycohydrolase. DNA Repair.

[31] Dexheimer TS, Pommier Y (2008). DNA cleavage assay for the identification of topoisomerase I inhibitors. Nat. Protoc..

[32] Anand J, Sun Y, Zhao Y, Nitiss KC, Nitiss JL (2018). Detection of topoisomerase covalent complexes in eukaryotic cells. Methods Mol. Biol..

[33] Chowdhuri, S.P. & Das, B.B. Top1-PARP1 association and beyond: from DNA topology to break repair. *NAR Cancer* **3**, 1 (2021). 10.1093/narcan/zcab003 PMC8095074 33981998

[34] Jongstra-Bilen J, Ittel ME, Niedergang C, Vosberg HP, Mandel P (1983). DNA topoisomerase I from calf thymus is inhibited in vitro by poly(ADP-ribosylation). Eur. J. Biochem..

[35] Debethune L, Kohlhagen G, Grandas A, Pommier Y (2002). Processing of nucleopeptides mimicking the topoisomerase I-DNA covalent complex by tyrosyl-DNA phosphodiesterase. Nucleic Acids Res..

[36] Interthal H, Chen HJ, Champoux JJ (2005). Human Tdp1 cleaves a broad spectrum of substrates, including phosphoamide linkages. J. Biol. Chem..

[37] Tomko RJ, Hochstrasser M (2013). Molecular architecture and assembly of the eukaryotic proteasome. Annu Rev. Biochem..

[38] Redinbo MR, Stewart L, Kuhn P, Champoux JJ, Hol WG (1998). Crystal structures of human topoisomerase I in covalent and noncovalent complexes with DNA. Science.

[39] Berko D (2012). The direction of protein entry into the proteasome determines the variety of products and depends on the force needed to unfold its two termini. Mol. Cell.

[40] Zhang YW (2011). Poly(ADP-ribose) polymerase and XPF-ERCC1 participate in distinct pathways for the repair of topoisomerase I-induced DNA damage in mammalian cells. Nucleic Acids Res..

[41] Chauhan D (2012). A small molecule inhibitor of ubiquitin-specific protease-7 induces apoptosis in multiple myeloma cells and overcomes bortezomib resistance. Cancer Cell.

[42] Schellenberg MJ (2017). ZATT (ZNF451)-mediated resolution of topoisomerase 2 DNA-protein cross-links. Science.

[43] Liao C (2018). UCHL3 regulates topoisomerase-induced chromosomal break repair by controlling TDP1 proteostasis. Cell Rep..

[44] Hudson JJ, Chiang SC, Wells OS, Rookyard C, El-Khamisy SF (2012). SUMO modification of the neuroprotective protein TDP1 facilitates chromosomal single-strand break repair. Nat. Commun..

[45] Lecona E (2016). USP7 is a SUMO deubiquitinase essential for DNA replication. Nat. Struct. Mol. Biol..

[46] Valles GJ, Bezsonova I, Woodgate R, Ashton NW (2020). USP7 is a master regulator of genome stability. Front Cell Dev. Biol..

[47] Jungmichel S (2013). Proteome-wide identification of poly(ADP-Ribosyl)ation targets in different genotoxic stress responses. Mol. Cell.

[48] Wei H, Yu X (2016). Functions of PARylation in DNA damage repair pathways. Genomics Proteom. Bioinform..

[49] Laco GS, Pommier Y (2008). Role of a tryptophan anchor in human topoisomerase I structure, function and inhibition. Biochem J..

[50] Langelier MF, Steffen JD, Riccio AA, McCauley M, Pascal JM (2017). Purification of DNA damage-dependent PARPs from E. coli for structural and biochemical analysis. Methods Mol. Biol..

[51] Crocker JC, Grier DG (1996). Methods of digital video microscopy for colloidal studies. J. Colloid Inter. Sci..

[52] Mazza D, Abernathy A, Golob N, Morisaki T, McNally JG (2012). A benchmark for chromatin binding measurements in live cells. Nucleic Acids Res..

[53] Saxton MJ, Jacobson K (1997). Single-particle tracking: applications to membrane dynamics. Annu Rev. Biophys. Biomol. Struct..

[54] Tarantino N (2014). TNF and IL-1 exhibit distinct ubiquitin requirements for inducing NEMO-IKK supramolecular structures. J. Cell Biol..

[55] Wisniewski JR, Zougman A, Nagaraj N, Mann M (2009). Universal sample preparation method for proteome analysis. Nat. Methods.

[56] Capranico G, Kohn KW, Pommier Y (1990). Local sequence requirements for DNA cleavage by mammalian topoisomerase II in the presence of doxorubicin. Nucleic Acids Res..

