# Supplementary material for: PARylation prevents the proteasomal degradation of topoisomerase I DNA-protein crosslinks and induces their deubiquitylation
Source: Nat Commun. 2021 Aug 18;12:5010. doi: 10.1038/s41467-021-25252-9 (PMC8373905; doi:10.1038/s41467-021-25252-9)
Supplement: Supplementary file 1 — Supplementary Information [file 41467_2021_25252_MOESM1_ESM.pdf]

Supplementary Figure 1

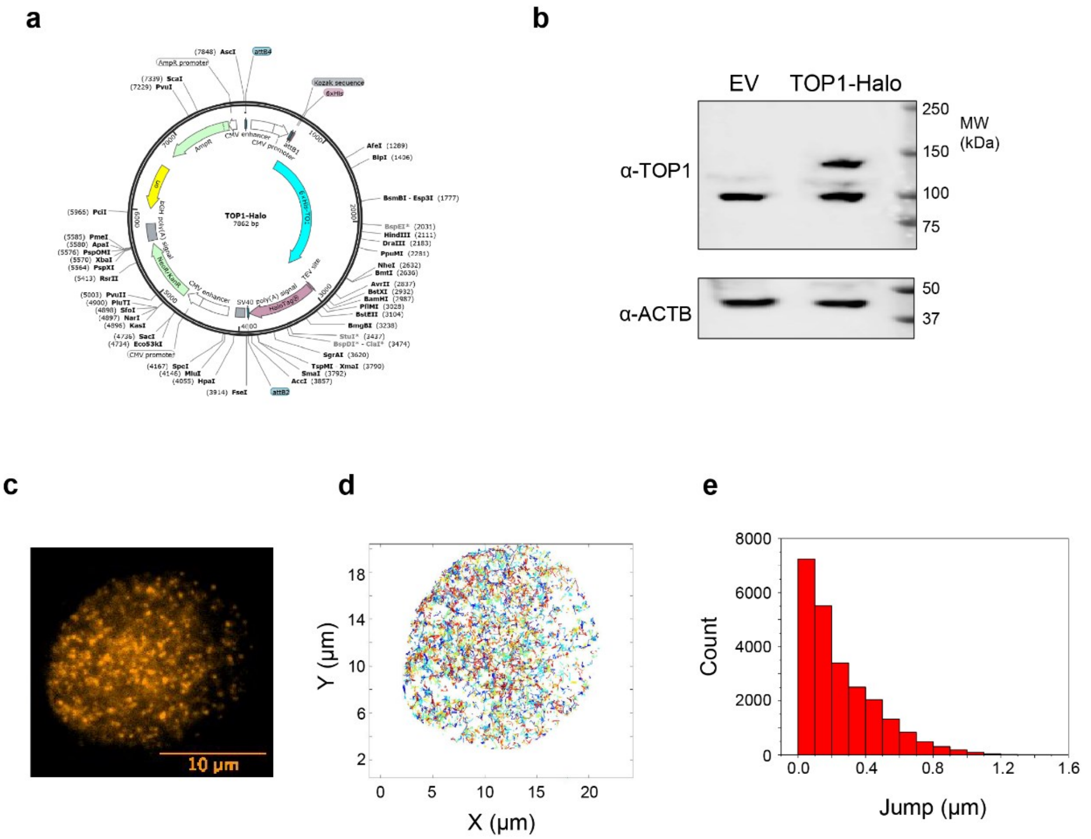

Supplementary Figure 1. TOP1 single molecule tracking under unperturbed condition

a. Map of the TOP1-HaloTag expressing plasmid (pUC19-CMV>Neo His6-TOP1-HaloTag) created by SnapGene (GSL Biotech).

b. Western blotting demonstrating the expression of TOP1-HaloTag proteins in U2OS cells.

c. Filming of TOP1-HaloTag single molecules in live U2OS cells for 20 seconds using single molecule fluorescence microscope.

d. Plot of tracks of TOP1-HaloTag single-molecules in the film in panel c. The tracks were reconstructed in two dimensions by MATLAB analysis pipeline. The neighboring tracks are of different colors to distinguish one from the others.

e. Count of jumps of TOP1-HaloTag single-molecules derived from panel c. The X axis of the histogram is jump distance of TOP1 single-molecules in the top-panel films and the Y axis is count of the jumps. The bin size is 0.1 micron.

Supplementary Figure 2

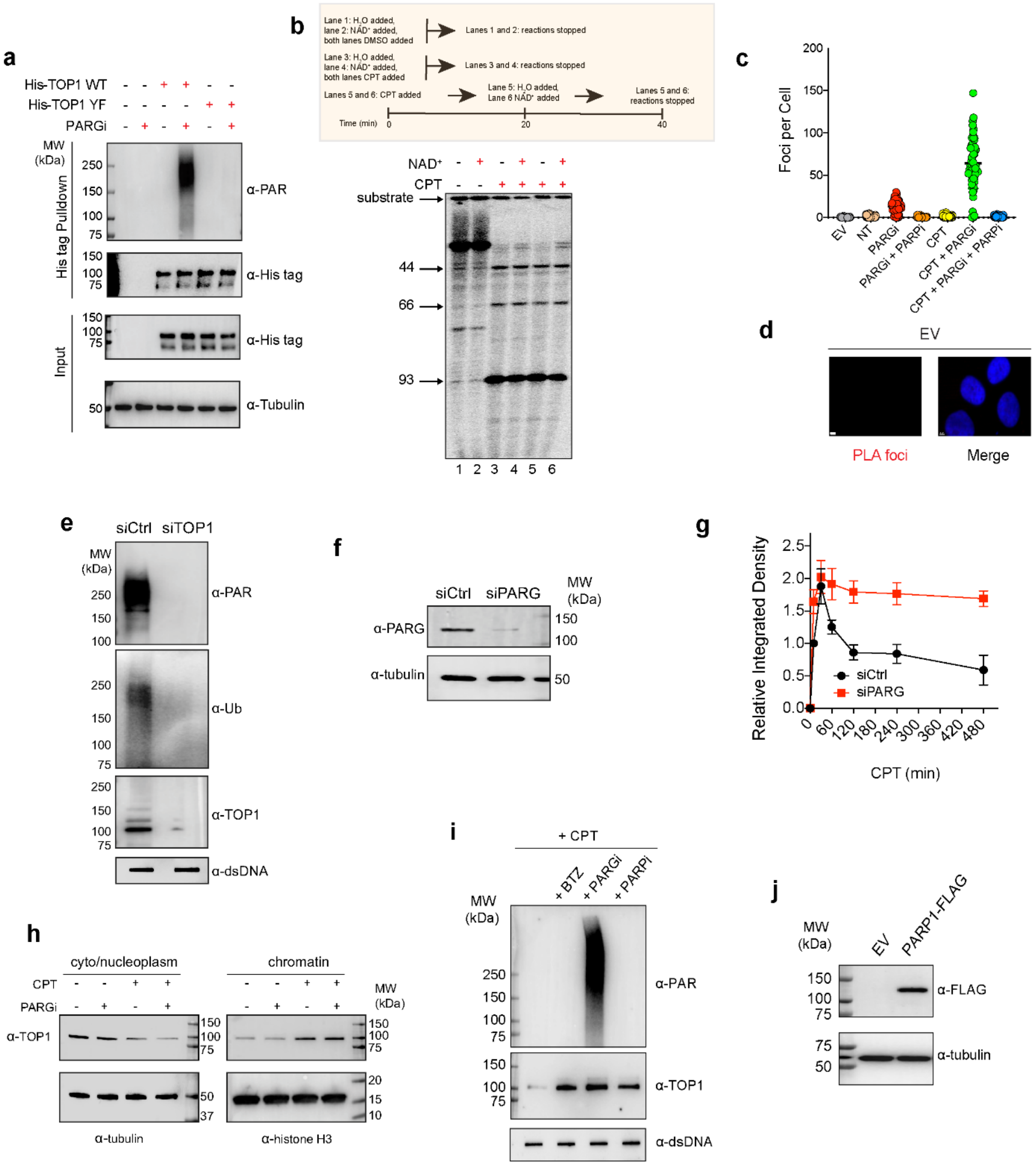

Supplementary Figure 2. Detection of TOP1-DPC PARylation *in vivo*

a. **His-pulldown assay showing that TOP1 WT but not catalytic mutant was a substrate of PARylation.** Following transfection of 6×His-tagged TOP1 WT or catalytic mutant (Y723F, abbreviated as YF) expression construct, HEK293 cells were treated with or without PARGi for His-tag pulldown using Ni-NTA agarose in denaturing condition. The pulldown samples and input samples were subjected to IB using α-TOP1 and α-PAR antibodies. PARGi: 10 μM, 2 h treatment.

b. **PARylation did not affect TOP1 cleavage activity.** Top panel represents the experimental protocol and bottom panel shows a representative gel. Lanes 1 and 2: Recombinant TOP1 was incubated with PARP1 in the absence (lane 1) or presence (lane 2) of NAD^+, immediately followed by incubation with a 117-bp 3'[^{32}P]-labeled oligonucleotide substrate encompassing previously identified TOP1 cleavage sites (see "Methods and Materials" for details) in the absence of CPT for 20 min. Lane 3 and 4: Recombinant TOP1 was incubated with PARP1 in the absence (lane 3) or presence (lane 4) of NAD^+, immediately followed by incubation with the oligonucleotide substrate in the presence of CPT (1 μM) for 20 min. Lane 5 and 6: Recombinant TOP1 was incubated with the oligonucleotide substrate in the presence of CPT (1 μM) for 20 min, then co-incubated with PARP1 in the absence (lane 5) or presence (lane 6) of NAD^+ for another 20 min. Each reaction mixture was subjected to denaturing PAGE and visualized by a phosphoImager and ImageQuant software.

c. Quantitation of PLA foci indicating TOP1-PAR interactions. Data were obtained from experiments as shown in Fig. 2b. n = 57 biologically independent cells.

d. No PLA foci were detected in U2OS cells transfected with empty vector (EV, pTrex).

e. **The modified RADAR assay confirming that PARylation detected by anti-PAR antibody was specific to TOP1-DPC.** Following transfection of control siRNA or siRNA targeting TOP1, HEK293 cells were treated CPT (20 μM, 1h) and PARGi for the modified RADAR assay to detect PARylation using anti-PAR antibody ubiquitylation using anti-Ub antibody and TOP1-DPCs using anti-TOP1 antibody.

f. WB in HEK293 cells confirming the knockdown efficiency of siRNA targeting PARG using anti-PARG antibody.

g. Densitometric analysis comparing TOP1-DPC signals generated from ICE assays including blots shown in Fig. 2f. Density of TOP1-DPCs/density of DNA of each group was normalized to siCtrl-transfected cells treated with CPT for 10 min. n = 3 independent experiments. Data are presented as mean values +/- standard deviation (SD).

h. **Subcellular fractionation assay showing that PARGi did not affect chromatin localization of TOP1.** HEK293 cells pre-treated with DMSO or PARGi (10 μM, 1h) were exposed to DMSO or CPT (20 μM) for 30 min, followed by subcellular protein fractionation and WB using indicated antibodies.

i. **The modified RADAR assay showing that inhibiting PARG but not the proteasome led to detectable TOP1-DPC PARylation.** DU145 cells were pre-treated with proteasome inhibitor (BTZ, 1 μM, 1h), PARGi (10 μM, 1h) or PARPi (10 μM, 1h), followed by CPT treatment (20 μM, 1h). Cells were then subjected to the TOP1-DPC PARylation detection assay for detection of TOP1-DPCs and their PARylation using α-TOP1 and α-PAR antibodies.

j. PARP1-FLAG protein expression in transfected HEK293 cells. EV: empty vector (pcDNA3).

Supplementary Figure 3

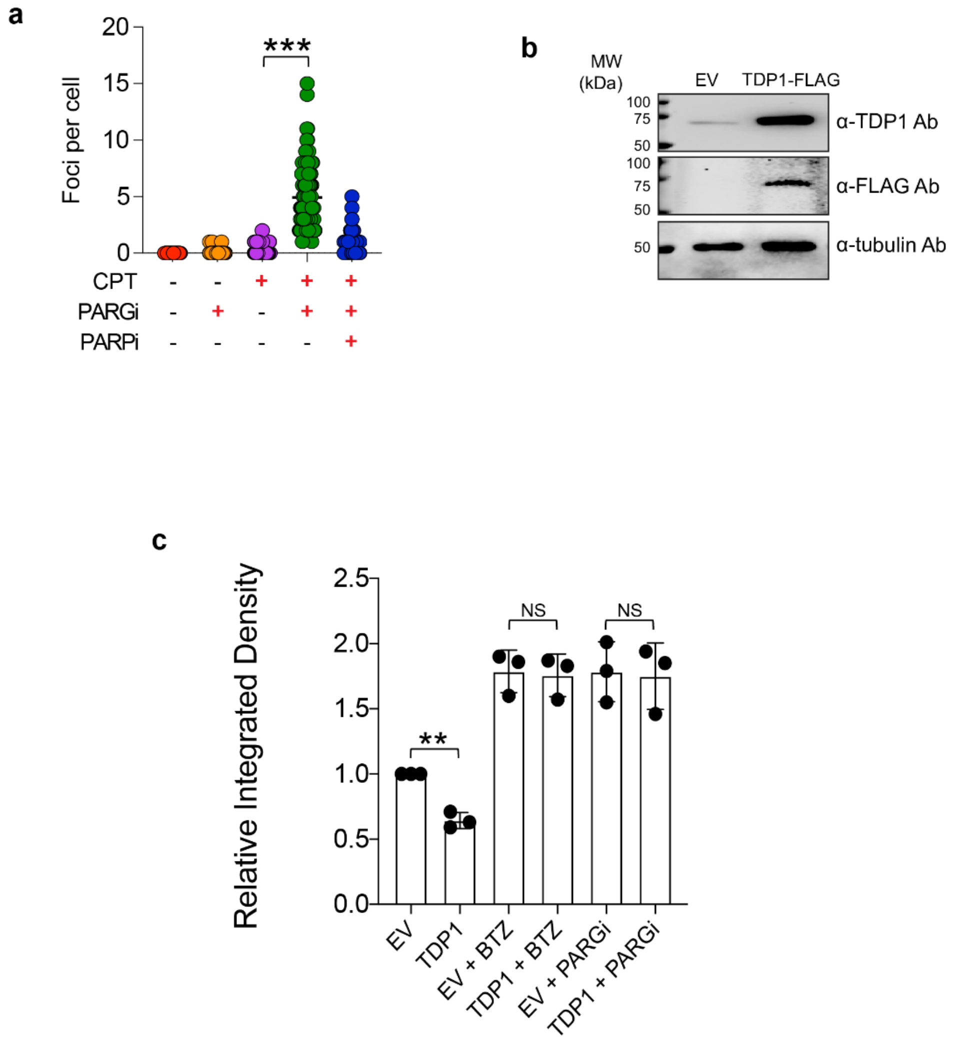

Supplementary Figure 3. PARylation is required for TOP1-TDP1 interaction

a. Quantitation of PLA samples shown in Fig. 3b. n = 107 biologically independent cells. P value was calculated by paired Student's t test (two-tailed distribution). ***: $p < 0.001$.

b. WB confirming the transfection efficiency of TDP1-FLAG expression plasmid using indicated antibodies.

c. Densitometric analysis comparing TOP1-DPC signals generated from ICE assays including blots shown in Fig. 3c. Density of TOP1-DPCs/density of DNA of each group was normalized to siCtrl-transfected cells treated with CPT for 10 min. NT: no transfection. EV: empty vector. n = 3 independent experiments. Data are presented as mean values +/- standard deviation (SD). P value was calculated by paired Student's t test (two-tailed distribution). **: $p = 0.009$. NS: not significant.

Supplementary Figure 4

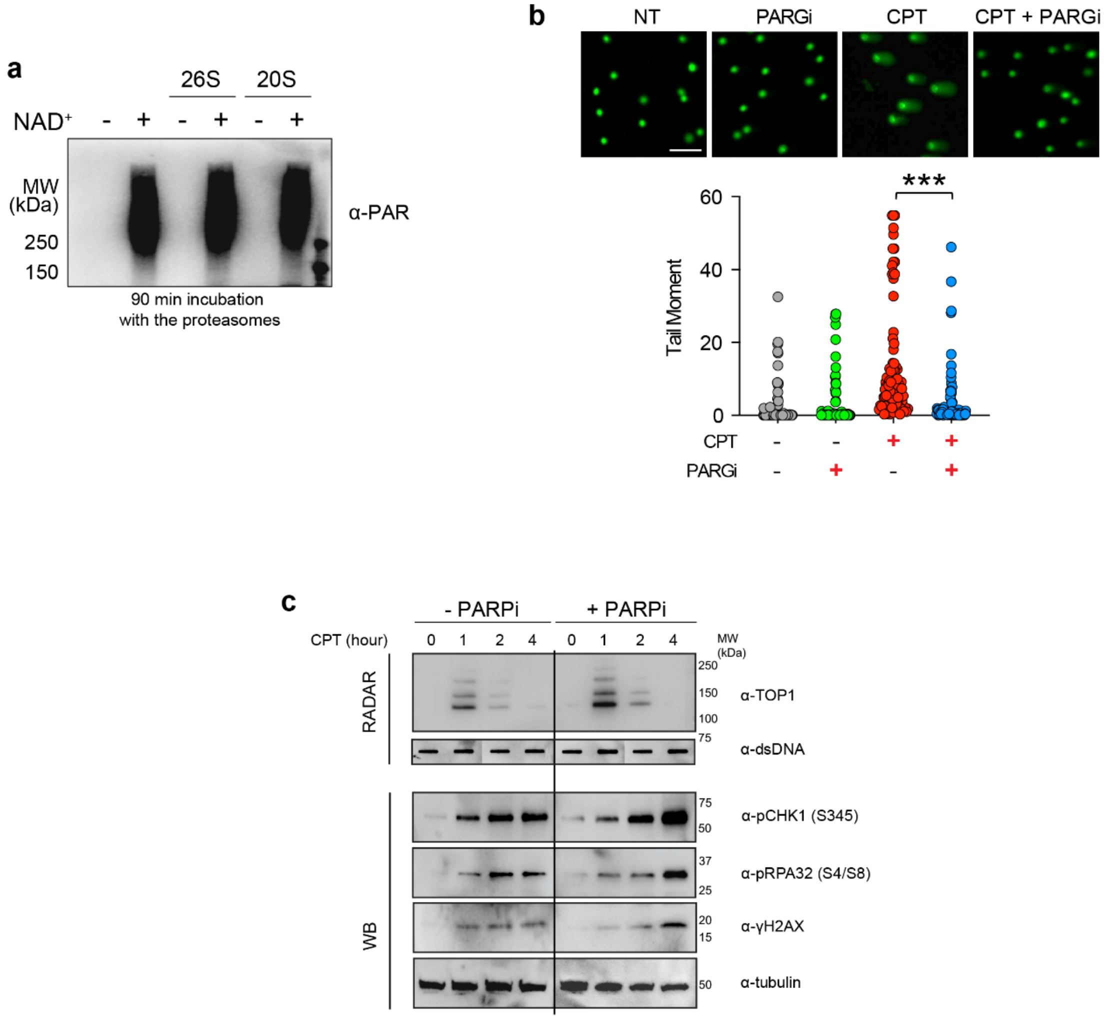

Supplementary Figure 4. PARylation blocks the proteasomal degradation of TOP1-DPCs

a. Probing the samples of Fig. 4b with α-PAR antibody confirms that PARylated TOP1-DPCs are not degraded by the 26s and 20S proteasomes.

b. **Neutral comet assay confirming that PARGi blocked the exposure of TOP1-concealed DSBs.** Upper panels: representative images of HEK293 cells treated with DMSO, CPT (10 μM, 2 h), PARGi (10 μM, 3 h) and CPT + PARGi (pre-treatment with PARGi for 1 h then co-treatment with CPT and PARGi for 2 h). Cells were subjected to neutral comet assay for detection of DNA DSBs. Lower panel: quantitation of tail moments using OpenComet. n = 94 biologically independent cells. P value was calculated by paired Student's t test (two-tailed distribution). ***: $p < 0.001$.

c. **The modified RADAR assay and WB showing that inhibiting PARP delayed but did not block the removal of TOP1-DPCs and the activation of DDR.** HEK293 cells were pre-treated with or without PARPi (10 μM, 1 h), followed by exposure to CPT (20 μM). Cells were collected at indicated time points and subjected either to the modified RADAR assay using indicated antibodies (upper panels) or to WB using indicated antibodies. (Lower panels).

Supplementary Figure 5

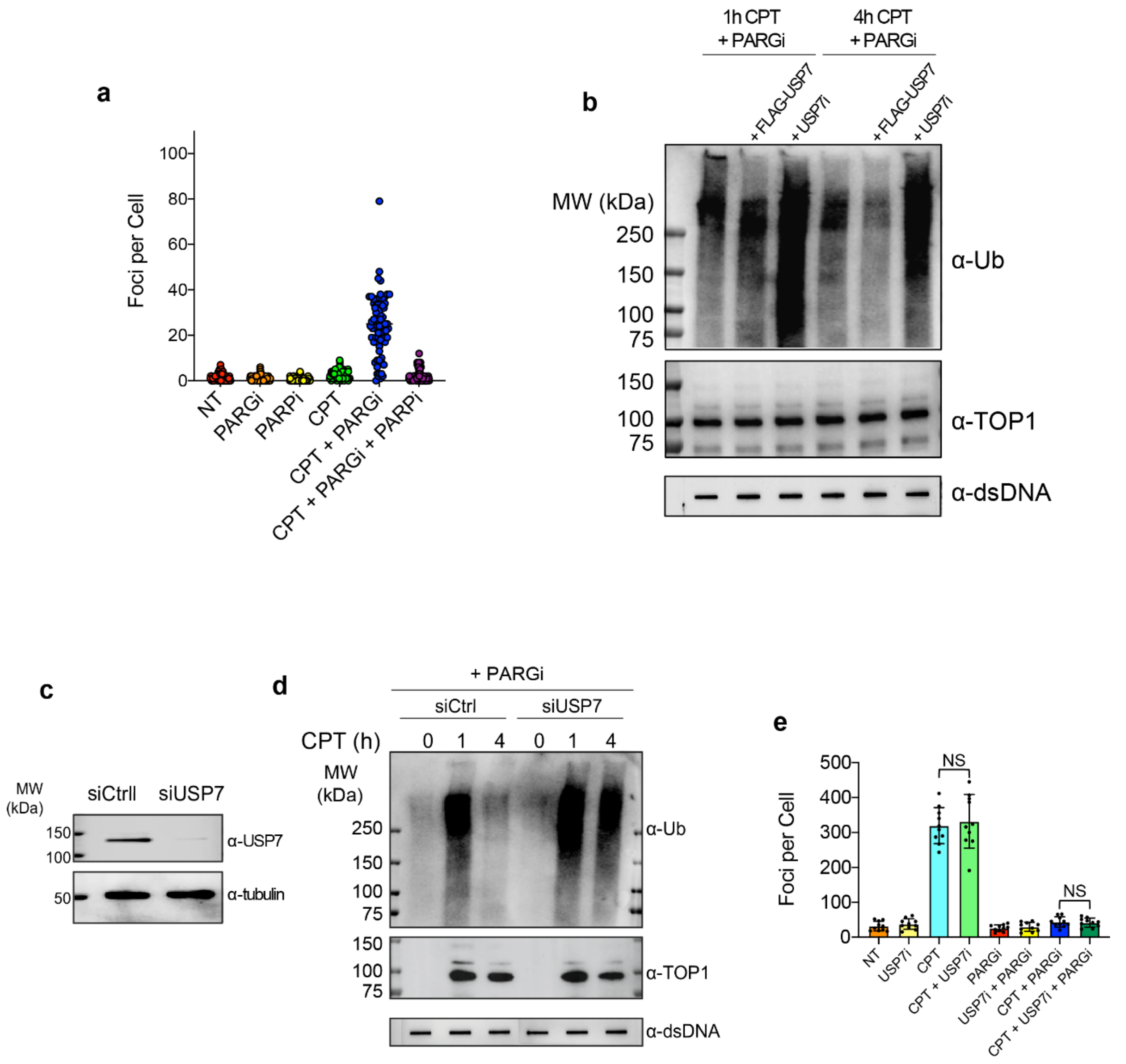

Supplementary Figure 5. PARylation induces USP7-mediated deubiquitylation of TOP1-DPCs

- **a.** Quantitation of PLA foci indicating TOP1-USP7 interactions. Data were obtained from experiments as shown in Fig. 5d. n = 73 biologically independent cells.
- **b.** **The modified RADAR assay showing that inhibiting USP7 in PARGi-treated cells prevented TOP1-DPC ubiquitylation.** Prior to CPT (20 μM) treatment, HEK293 cells were pre-treated with PARGi (10 μM, 1h) and divided into the following groups: CPT (1 h), CPT (1h) + FLAG-USP7 transfection, CPT (1h) + USP7i (10 μM, 1h pre-treatment), CPT (4 h), CPT (4h) + FLAG-USP7 transfection, CPT (4h) + USP7i. Following the treatments, cells were subjected to the modified RADAR assay for detection of TOP1-DPCs and their ubiquitylation using α-TOP1 and α-Ub antibodies.
- **c.** WB confirming the knockdown efficiency of USP7 siRNA using indicated antibodies.

d. **The modified RADAR assay showing that downregulating USP7 by siRNA prevented TOP1-DPC deubiquitylation.** Prior to CPT (20 μM) treatment, HEK293 cells transfected with control or USP7 siRNA were pre-treated with PARGi (10 μM, 1h). Following the treatments, cells were collected at indicated time points and subjected to the modified RADAR assay for detection of TOP1-DPCs and their ubiquitylation using α-TOP1 and α-Ub antibodies.

e. Quantitation of γH2AX foci. Data were obtained from experiments as shown in Fig. 5f. n = 10 independent cells. Data are presented as mean values +/- standard deviation (SD). P value was calculated by paired Student's t test (two-tailed distribution). NS: not significant.

Supplementary Table 1. Primers used for construction and sequencing of pUC19-CMV>Neo His6-TOP1-HaloTag

**Construction**	
200313-3420tkf-PF1	CAACTTTGTACAAAAAAGCAGGCTGCCACCATGGCCCATCATCACCATCACC
200313-3420tkf-PR1	GCTCTGAAAGTACAGATCCTCAAACTCATAGTCTTCATCAGCCATGTC
200313-3420tkf-PF2	GCTGATGAAGACTATGAGTTTGAGGATCTGTACTTTCAGAGCGAT
200313-3420tkf-PR2	CAACTTTGTACAAGAAAGCTGGGTTTAACCGGAAATCTCCAGAGTAGAC
**Sanger Sequencing**	
pUp-pDown-flank-F	TGGGCGTGGATAGCGGTTTGAC
Seq-200313-3420tkf-PF1	TGAGGATGATGCTGATTATAAACC
Seq-200313-3420tkf-PF2	GAAAAATTACTGAAAGAATATGGAT
Seq-200313-3420tkf-PF3	ATATGTGGTAGAGTTTGACTTCCTC
Seq-200313-3420tkf-PF4	CTGGGAACCTCCAAACTCAATTATC
pUp-pDown-flank-R	CTAGTTGTGGTTTGTCCAAACTC
