# Supplementary material for: PARylation prevents the proteasomal degradation of topoisomerase I DNA-protein crosslinks and induces their deubiquitylation
Source: Nat Commun. 2021 Aug 18;12:5010. doi: 10.1038/s41467-021-25252-9 (PMC8373905; doi:10.1038/s41467-021-25252-9)
Supplement: Supplementary file 3 — Description of Additional Supplementary Files [file 41467_2021_25252_MOESM3_ESM.pdf]

Description of Additional Supplementary Files

File Name: Supplementary Data 1
Description: Topoisomerase 1 interactome by His pulldown-LC-MS.

File Name: Supplementary Movie 1
Description: TOP1 single molecule tracking under unperturbed condition.

File Name: Supplementary Movie 2
Description: TOP1 single molecule tracking before treatment.

File Name: Supplementary Movie 3
Description: TOP1 single molecule tracking after DMSO.

File Name: Supplementary Movie 4
Description: TOP1 single molecule tracking after BTZ.

File Name: Supplementary Movie 5
Description: TOP1 single molecule tracking after PARGi.

File Name: Supplementary Movie 6
Description: TOP1 single molecule tracking after CPT.

File Name: Supplementary Movie 7
Description: TOP1 single molecule tracking after CPT + BTZ.

File Name: Supplementary Movie 8
Description: TOP1 single molecule tracking after CPT + PARGi.

File Name: Supplementary Movie 9
Description: TOP1 single molecule tracking DMSO time course.

File Name: Supplementary Movie 10
Description: TOP1 single molecule tracking CPT time course.

File Name: Supplementary Movie 12
Description: TOP1 single molecule tracking CPT + PARGi time course.

File Name: Supplementary Movie 12
Description: TOP1 single molecule tracking CPT +BTZ time course.
