# Supplementary material for: PARylation prevents the proteasomal degradation of topoisomerase I DNA-protein crosslinks and induces their deubiquitylation
Source: Nat Commun. 2021 Aug 18;12:5010. doi: 10.1038/s41467-021-25252-9 (PMC8373905; doi:10.1038/s41467-021-25252-9)
Supplement: Supplementary file 17 — Reporting Summary [file 41467_2021_25252_MOESM17_ESM.pdf]

nature portfolio

Corresponding author(s): Yilun Sun and Yves Pommier

Last updated by author(s): Jun 15, 2021

Reporting Summary

Nature Portfolio wishes to improve the reproducibility of the work that we publish. This form provides structure for consistency and transparency in reporting. For further information on Nature Portfolio policies, see our Editorial Policies and the Editorial Policy Checklist.

Statistics

For all statistical analyses, confirm that the following items are present in the figure legend, table legend, main text, or Methods section.

n/a	Confirmed	
☐	☒	The exact sample size (*n*) for each experimental group/condition, given as a discrete number and unit of measurement
☐	☒	A statement on whether measurements were taken from distinct samples or whether the same sample was measured repeatedly
☐	☒	The statistical test(s) used AND whether they are one- or two-sided<br>*Only common tests should be described solely by name; describe more complex techniques in the Methods section.*
☒	☐	A description of all covariates tested
☒	☐	A description of any assumptions or corrections, such as tests of normality and adjustment for multiple comparisons
☐	☒	A full description of the statistical parameters including central tendency (e.g. means) or other basic estimates (e.g. regression coefficient) AND variation (e.g. standard deviation) or associated estimates of uncertainty (e.g. confidence intervals)
☐	☒	For null hypothesis testing, the test statistic (e.g. *F*, *t*, *r*) with confidence intervals, effect sizes, degrees of freedom and *P* value noted<br>*Give P values as exact values whenever suitable.*
☒	☐	For Bayesian analysis, information on the choice of priors and Markov chain Monte Carlo settings
☒	☐	For hierarchical and complex designs, identification of the appropriate level for tests and full reporting of outcomes
☒	☐	Estimates of effect sizes (e.g. Cohen's *d*, Pearson's *r*), indicating how they were calculated

Our web collection on statistics for biologists contains articles on many of the points above.

Software and code

Policy information about availability of computer code

Data collection	Image Lab Software for PC Version 6.1; NIS-Elements Viewer
Data analysis	ImageJ; Matlab R2020a; Prism 8.0; Origin Lab 2019b

For manuscripts utilizing custom algorithms or software that are central to the research but not yet described in published literature, software must be made available to editors and reviewers. We strongly encourage code deposition in a community repository (e.g. GitHub). See the Nature Portfolio guidelines for submitting code & software for further information.

Data

Policy information about availability of data

All manuscripts must include a data availability statement. This statement should provide the following information, where applicable:

- Accession codes, unique identifiers, or web links for publicly available datasets
- A description of any restrictions on data availability
- For clinical datasets or third party data, please ensure that the statement adheres to our policy

All the raw images generated in this study have been deposited in the Mendeley database under accession code http://dx.doi.org/10.17632/cm6vk7y8vj.1
It is now publicly accessible.

Field-specific reporting

Please select the one below that is the best fit for your research. If you are not sure, read the appropriate sections before making your selection.

☒ Life sciences ☐ Behavioural & social sciences ☐ Ecological, evolutionary & environmental sciences

For a reference copy of the document with all sections, see nature.com/documents/nr-reporting-summary-flat.pdf

Life sciences study design

All studies must disclose on these points even when the disclosure is negative.

Sample size	Sample size was determined to ensure statistical analyses. No statistical methods were used to predetermine sample size. For most of the experiments, 4-6 samples were used to compare no treatment, PARG inhibitor only, camptothecin only, camptothecin + PARG inhibitor, camptothecin + PARP inhibitor and camptothecin + proteasome inhibitor to investigate the role of PARG for repair of topoisomerase I DNA-protein crosslinks
Data exclusions	No data exclusions
Replication	ICE assays, His tag pulldown and modified RADAR assays were successfully repeated for 3 times. Single molecule imaging, confocal, PLA, comet assays and iSIM microscopic analyses were successfully repeated two times.
Randomization	The experiments were not randomized because this work is not a randomized study on drug efficacy and safety in animal models or human. We used certain cell lines and treat them with certain drugs in controlled and deliberate manners to study the mechanisms repairing topoisomerase I DNA-protein crosslinks at molecular levels.
Blinding	The investigators were not blinded to allocation during experiments and outcome assessment. The investigate needed to design and control the experiments for treating certain cells with certain drugs for the study. The investigators had to know what cell lines and what drugs they are dealing with.

Reporting for specific materials, systems and methods

We require information from authors about some types of materials, experimental systems and methods used in many studies. Here, indicate whether each material, system or method listed is relevant to your study. If you are not sure if a list item applies to your research, read the appropriate section before selecting a response.

Materials & experimental systems

n/a	Involved in the study	
☐	☒	Antibodies
☐	☒	Eukaryotic cell lines
☒	☐	Palaeontology and archaeology
☒	☐	Animals and other organisms
☒	☐	Human research participants
☒	☐	Clinical data
☒	☐	Dual use research of concern

Methods

n/a	Involved in the study	
☒	☐	ChIP-seq
☒	☐	Flow cytometry
☒	☐	MRI-based neuroimaging

Antibodies

Antibodies used	anti-PAR, mouse monoclonal, Trevigen, 4335-MC-100; anti-PAR (clone 10H), mouse monoclonal, Enzo Life Sciences, ALX-804-220; anti-ubiquitin, mouse monoclonal (clone P4D1), Santa Cruz, sc-8017; anti-His tag (clone His. H8), mouse monoclonal, Abcam, ab18184; anti-His tag (clone D3I10), rabbit monoclonal, Cell signaling, 12698; anti-TOP1 (clone C21), mouse monoclonal, BD Biosciences, 556597; anti-dsDNA (clone 3519), mouse monoclonal, Abcam, ab27156; anti-FLAG (clone M2), mouse monoclonal, Sigma Aldrich, F1804; anti-FLAG, rabbit polyclonal, Sigma Aldrich, F7425; anti-TDP1, rabbit polyclonal, Bethyl Laboratories, A301-618A; anti-PARP1 (clone F-2), mouse monoclonal, Santa Cruz, sc-8007; anti-PARG, rabbit monoclonal, Cell Signaling, 66564; anti-γH2AX, rabbit polyclonal, Cell Signaling, 66564; anti-BrdU (clone IIb5), mouse monoclonal, Abcam, ab8152; anti-USP7, rabbit polyclonal, Bethyl Lab, A300-033A. Goat anti-Rabbit IgG (H+L) Cross-Adsorbed Secondary Antibody, Alexa Fluor 568, Thermofisher,Catalog # A-11011. Goat anti-Mouse IgG (H+L), Superclonal Recombinant Secondary Antibody, Alexa Fluor 488, Thermofisher,Catalog # A-28175. All the primary antibodies were used in 1:1000 dilution except anti-PAR, mouse monoclonal, Trevigen, 4335-MC-100; anti-PAR (clone 10H), mouse monoclonal, Enzo Life Sciences, ALX-804-220; anti-ubiquitin, mouse monoclonal (clone P4D1), Santa Cruz, sc-8017, which were used in 1: 250 dilution. All the secondary antibodies were used in 1: 5000 dilution.
Validation	anti-PAR, mouse monoclonal, Trevigen, 4335-MC-100 was validated for western blotting (WB) and immunoprecipitation (IP); anti-PAR (clone 10H), mouse monoclonal, Enzo Life Sciences, ALX-804-220 was validated for WB; anti-ubiquitin, mouse monoclonal (clone P4D1), Santa Cruz, sc-8017 was validated for WB; anti-His tag (clone His. H8), mouse monoclonal, Abcam, ab18184 was validated for

WB, pulldown and immunofluorescence (IF); anti-His tag (clone D3I10), rabbit monoclonal, Cell signaling, 12698 was validated for WB , pulldown and IF; anti-TOP1 (clone C21), mouse monoclonal, BD Biosciences, 556597 was validated for WB; anti-dsDNA (clone 3519), mouse monoclonal, Abcam, ab27156 was validated for slot-blot; anti-FLAG (clone M2), mouse monoclonal, Sigma Aldrich, F1804 was validated for WB, IF and IP; anti-FLAG, rabbit polyclonal, Sigma Aldrich, F7425 was validated for WB, IF and IP; anti-TDP1, rabbit polyclonal, Bethyl Laboratories, A301-618A was validated for WB; anti-PARP1 (clone F-2), mouse monoclonal, Santa Cruz, sc-8007 was validated for WB; anti-PARG, rabbit monoclonal, Cell Signaling, 66564 was validated for WB; anti-γH2AX, rabbit polyclonal, Cell Signaling, 66564 was validated for WB and IF; anti-BrdU (clone IIb5), mouse monoclonal, Abcam, ab8152 as validated for IF; anti-USP7, rabbit polyclonal, Bethyl Lab, A300-033Aas validated for WB.

Eukaryotic cell lines

Policy information about cell lines

Cell line source(s)	HEK293 cells and U2OS cells were obtained from the NCI Development Therapeutics Program.
Authentication	Cell Line Authentication was carried out using Short Tandem Repeat Analysis at Frederick National Laboratory.
Mycoplasma contamination	Cells were routinely tested for mycoplasma by MycoAlert (Lonza) and found negative.
Commonly misidentified lines (See ICLAC register)	No commonly misidentified cell line was used.
