# Supplementary material for: PARylation prevents the proteasomal degradation of topoisomerase I DNA-protein crosslinks and induces their deubiquitylation
Source: Nat Commun. 2021 Aug 18;12:5010. doi: 10.1038/s41467-021-25252-9 (PMC8373905; doi:10.1038/s41467-021-25252-9)

Figure 2a

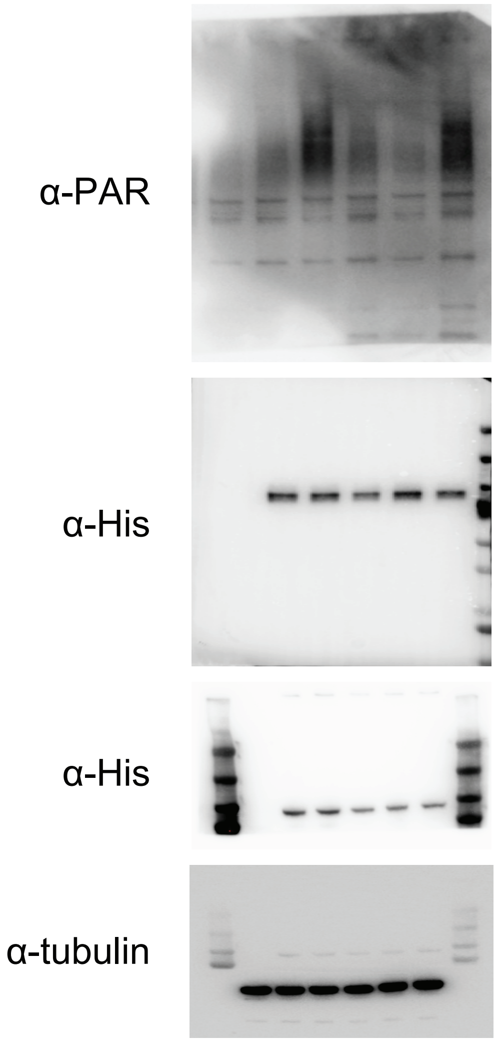

Figure 3a

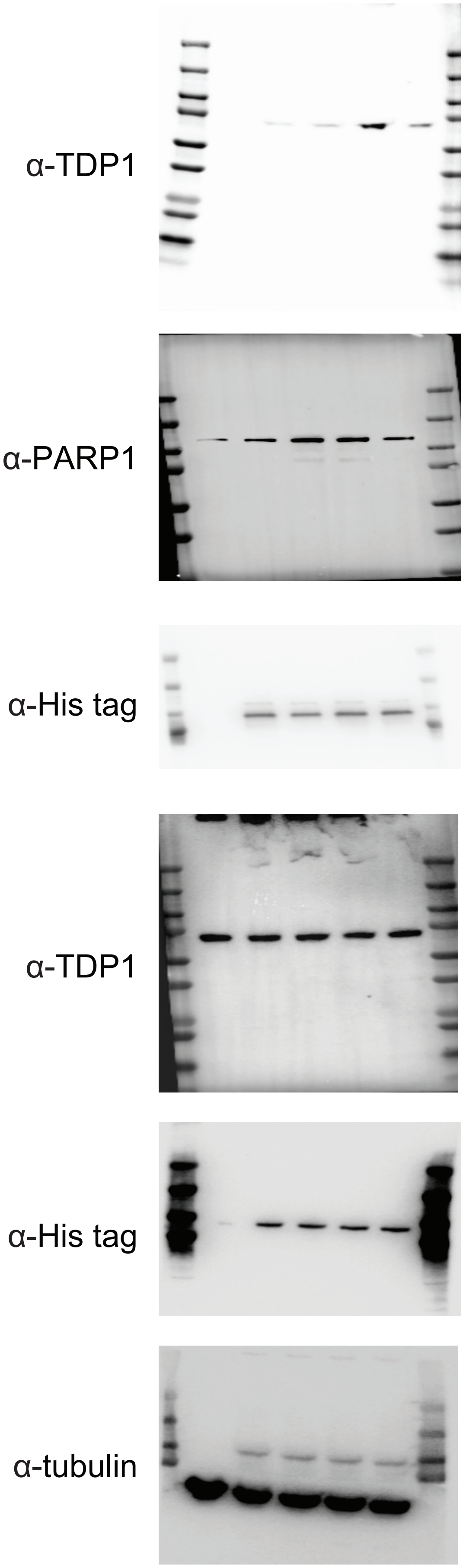

Figure 4c

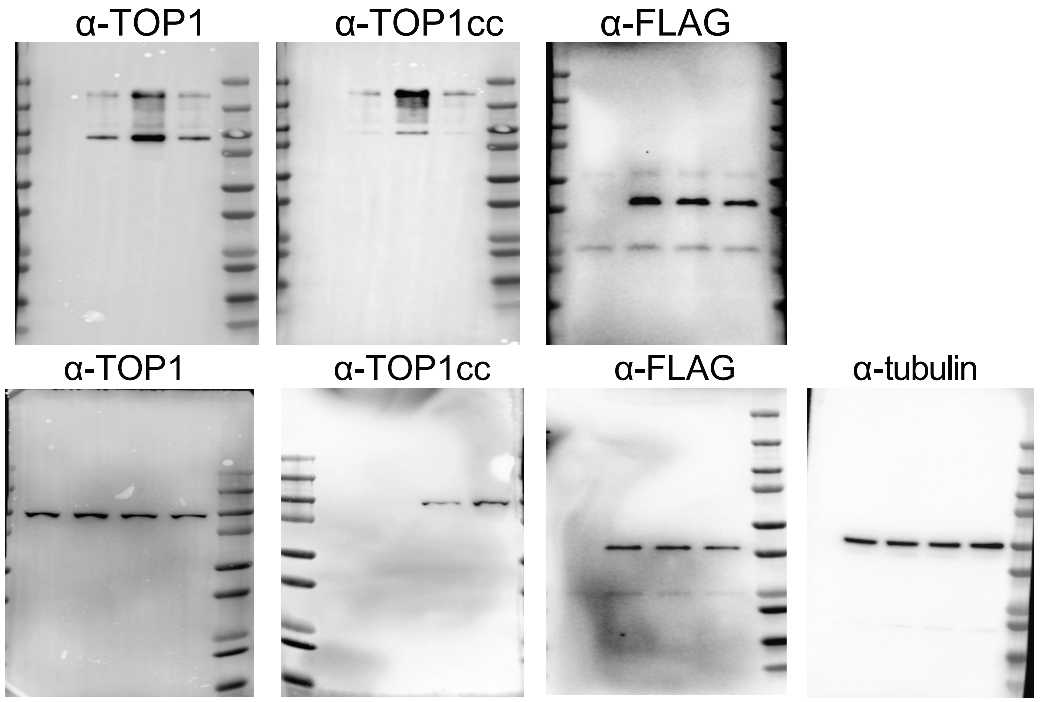

Figure 4d

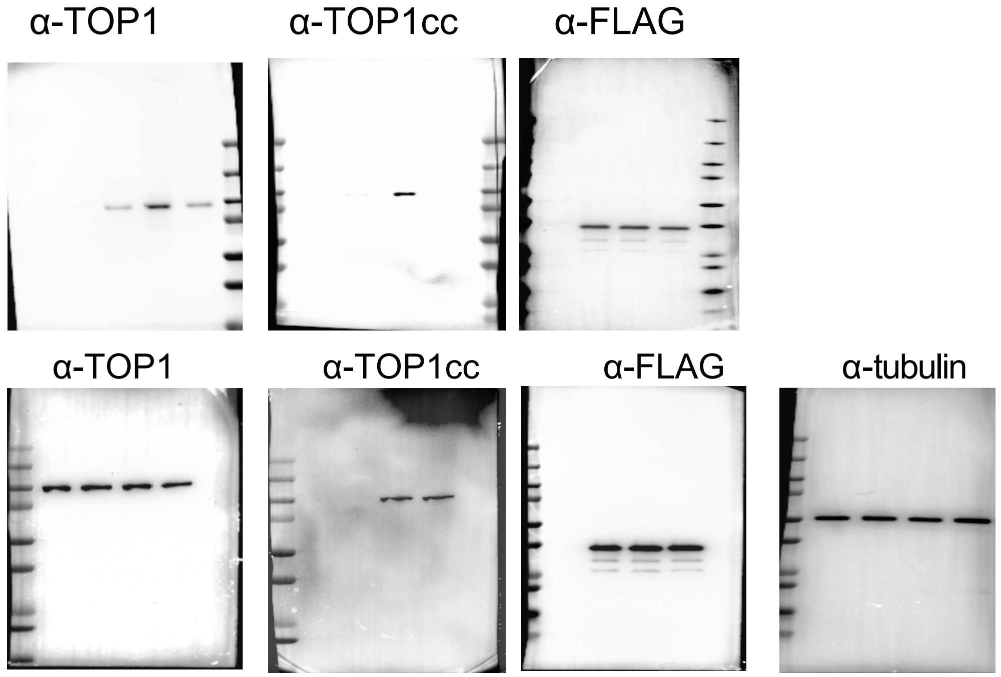

Figure 4g

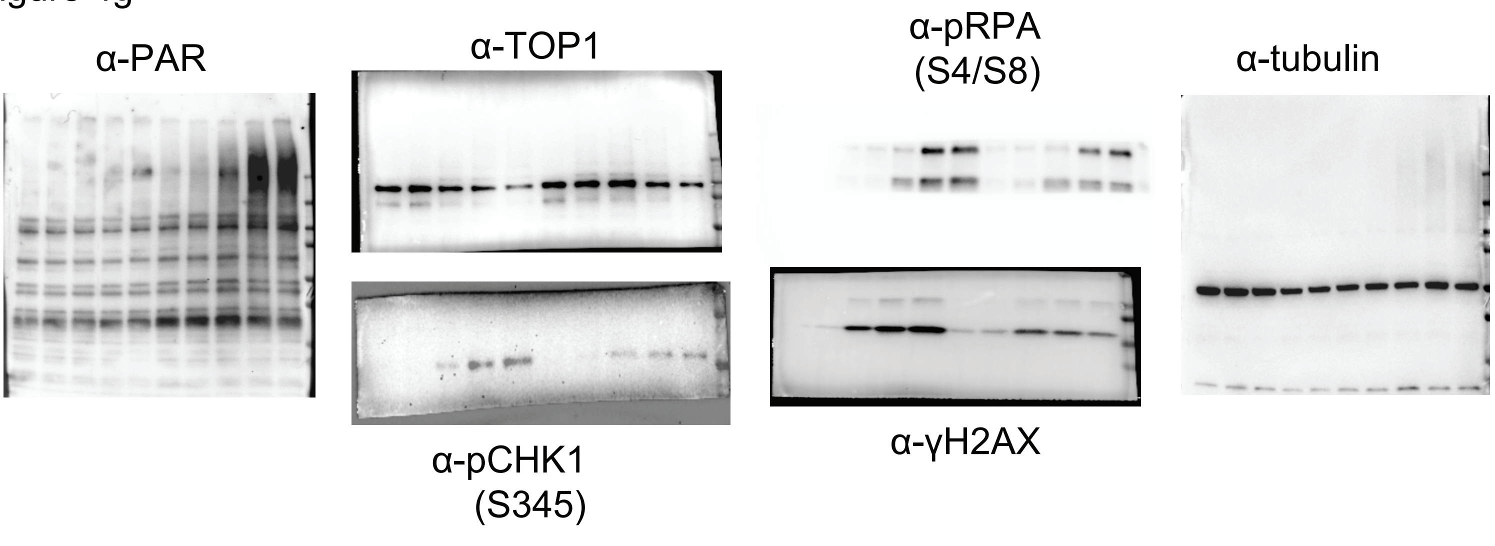

Figure 5c

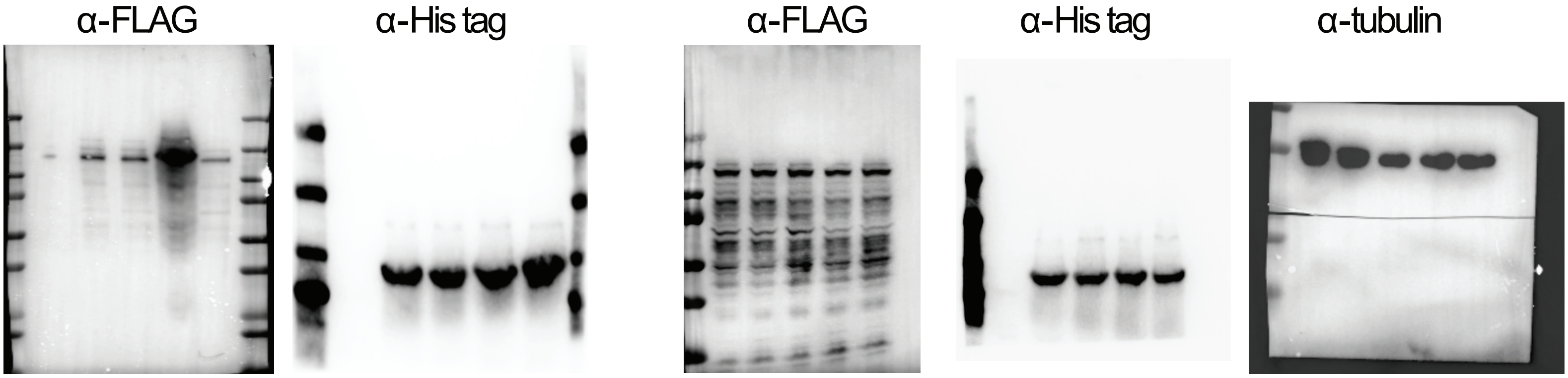

Supplementary Fig. 1b

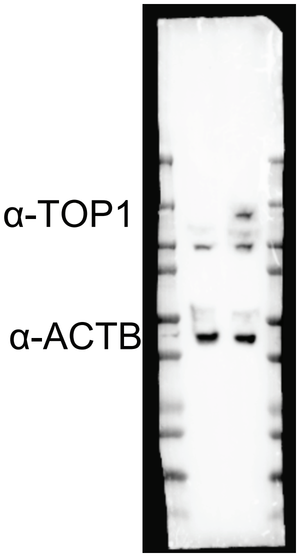

Supplementary Fig. 2a

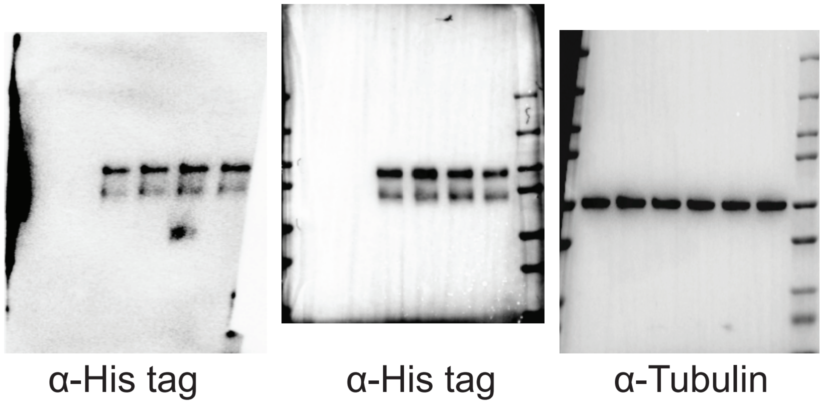

Supplementary Fig. 2f

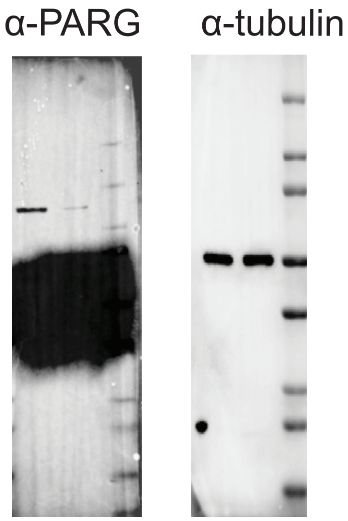

Supplementary Fig. 2h

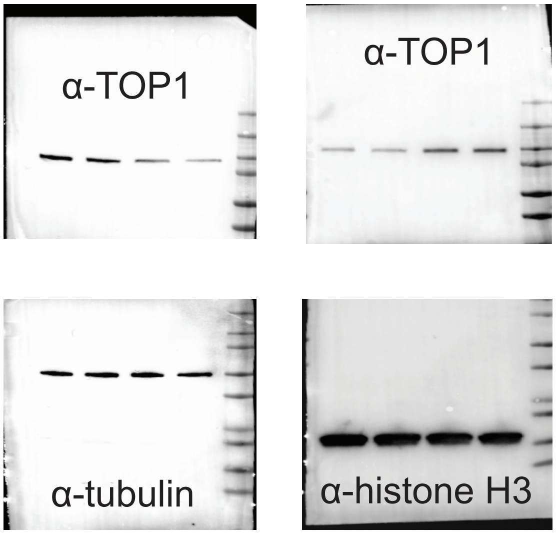

Supplementary Fig. 2i

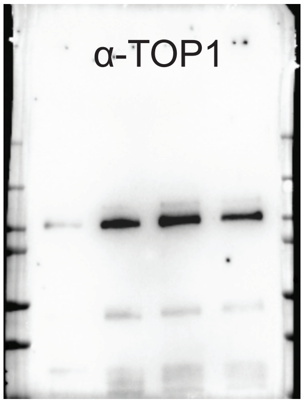

Supplementary Fig. 2j

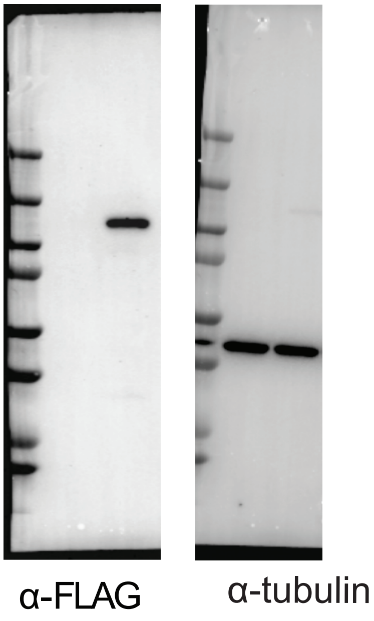

Supplementary Fig. 3b

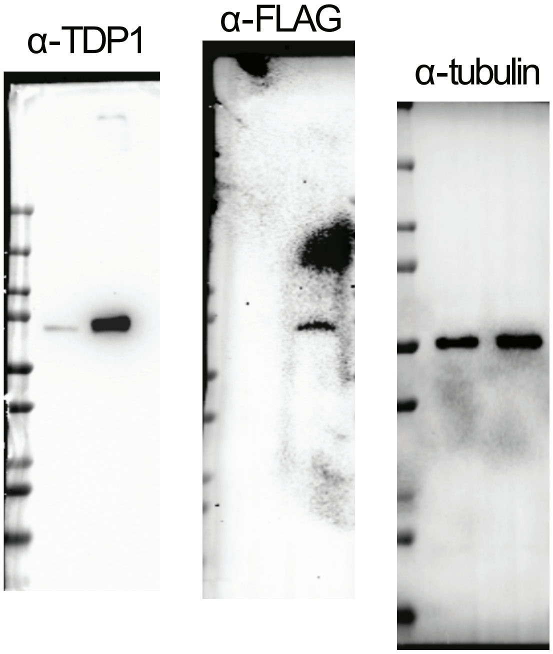

Supplementary Fig. 4a

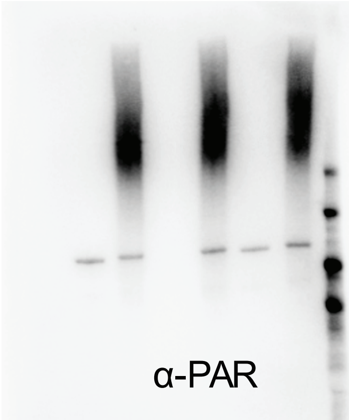

Supplementary Fig. 4a

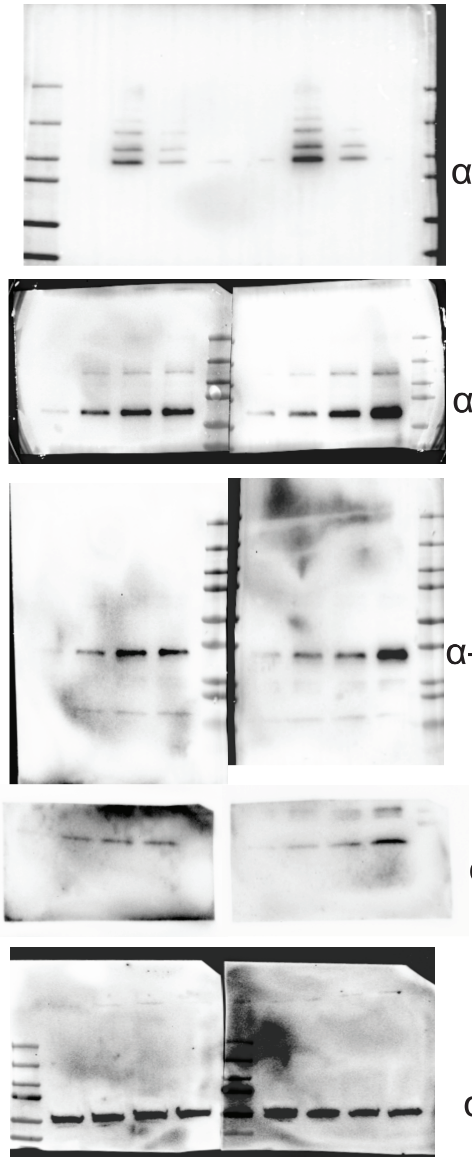

Supplementary Fig. 5c

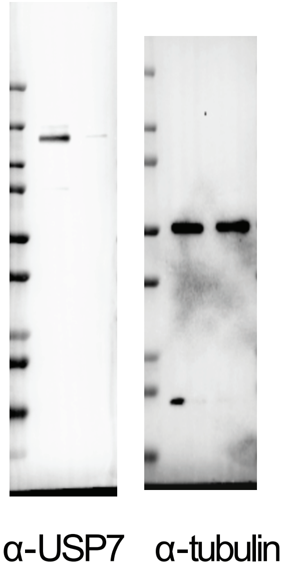

Supplementary Fig. 5d

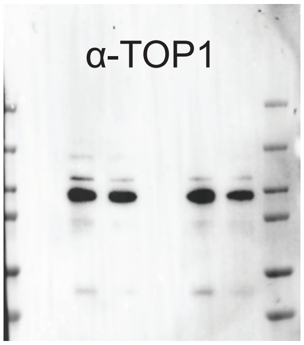

Supplement: Supplementary file 18 — Source Data [file 41467_2021_25252_MOESM18_ESM.zip › Source data/Uncropped blots.pdf]
