# Supplementary material for: PARylation prevents the proteasomal degradation of topoisomerase I DNA-protein crosslinks and induces their deubiquitylation
Source: Nat Commun. 2021 Aug 18;12:5010. doi: 10.1038/s41467-021-25252-9 (PMC8373905; doi:10.1038/s41467-021-25252-9)
Supplement: Supplementary file 2 — Peer Review File [file 41467_2021_25252_MOESM2_ESM.pdf]

REVIEWER COMMENTS

Reviewer #1 (Remarks to the Author):

DNA topoisomerase I (TOP1) is essential for transcription and DNA replication. Camptothecin (CPT), a TOP1 poison, is widely used for anti-cancer therapy. CPT generates vast numbers of TOP1 DNA-protein crosslinks (TOP1-DPCs), and the resulting single-strand breakage (SSB) is converted to one-end-breaks during DNA replication. Cells deficient in Poly[ADP ribose]polymerase 1 (PARP1) are hypersensitive to CPT (Pommier Y. et al., Nucleic Acids Res. 2014, PMID: 24493735), and the molecular mechanism underlying the hypersensitivity has not yet been fully understood. PARP1 is involved in a number of repair pathways including SSB repair and protects DNA replication forks from one-end-breaks. Sun Y. and Pommier Y. previously reported that the removal of TOP1 adducts from TOP1-DPCs by the TDP1 phosphodiesterase needs preceding partial digestion of TOP1 adducts, "debulking" by the RNF4 ubiquitin ligase and proteasome so that TDP1 can access the crosslink junction (Sci Adv. 2020, PMID: 33188014). The current work revealed that the inhibition of PARG and resulting excess TOP1 PARylation block the debulking of TOP1-DPCs. All the data are impressively robust, and biochemical data agree with cellular experiments including western blot of immune-precipitated samples. This report has made substantial advances in understanding the molecular mechanisms for the two key anti-cancer agents, the TOP1 poison and PARP inhibitor (PARPi). The reviewer, therefore, recommend the publication of this work in Nature Communications. However, general readers could not understand several figures, and the reviewer requests the rewording and additional explanation of the following figures (particularly Fig.2d and Fig.4c and 4d).

Comments:
1. General readers as well as the reviewer are not familiar with the cutting-edge analysis using the single-molecule fluorescence microscopy (Fig.1a), and the authors need to explain the analysis further. The TOP1-Halo-Tag signals of the top panels do not seem to agree with the histogram of the bottom (Fig.1a); although signals of "Before treatment", "CPT＋PARGi", and "CPT+BTZ" are similar in the top panels, the histogram shows that the jump of signals "Before treatment" are much higher than that of "CPT＋PARGi" and "CPT+BTZ". The authors need to clearly explain the difference in signals between the top and middle panels, "the most mobile TOP1 molecules" shown in the pink area of the histogram, and "jump", the x-axis of the histogram.
2. CPT causes the trap of TOP1 on genomic DNA. Why did the CPT treatment increase the percentage of "the most mobile TOP1 molecules"? Did the treatment with 100 uM CPT for 2 h trap virtually all TOP1 molecules on the genomic DNA? To show the biological significance of "the most mobile TOP1 molecules", the authors need to show the effect of limited concentrations of CPT in a supplementary figure using the method shown in Fig.1a.
3. The authors need to show that the analysis of Fig.2a was done in a denaturing pull-down condition, but not in a native pull-down, in Figure legend.
4. The following conclusion, “PARG inhibition reduced the levels of Top1-DPC ubiquitination after 1 hour CPT treatment, suggesting that persistent PARylation of TOP1-DPC leads to their deubiquitination” (line 6th from the bottom of page 4) might not be justified, as the Top1-DPC ubiquitination signal on lane 4 of Fig.2d (without PARG inhibition) is similar to that on lane 11 (with PARG inhibition). Fig.4d seems to show that PARG inhibitor (PARGi) promoted the Top1-DPC formation (at 10 min on lane 9 in the bottom panel), increased and then decreased the ubiquitination earlier than the PARGi(-) samples, and completely prevented the degradation of TOP1. The authors need to reword the conclusion written on line 6th from the bottom of page 4.
5. Fig.2d indicates the relationship between TOP1 ubiquitination, TOP1 PARylation, and the stability of Top1-DPC and clearly shows that the stability of Top1-DPC depends on the PARylation rather than ubiquitination during the 8 h CPT treatment. The authors need to state a potential negative effect of TOP1 PARylation on the proteosome-mediated degradation of Top1-DPC rather than ““PARG inhibition reduced the levels of Top1-DPC ubiquitination”. This statement is helpful for general readers to understand a logical connection between Fig.2d and Fig.4a to 4d, which show that TOP1 PARylation indeed inhibits the digestion of TOP1 by the proteosome in both in vitro (Fig.4b) and in vivo (Fig.4c and d).
6. The authors need to show that the analysis of Fig.3a was done in a native pull-down condition, but not in a denaturing pull-down, in Figure legend.
7. Fig.3a and 3b show the interaction between intact TOP1 (keeping antigenicity for the western

blot analysis) and TDP1. While TDP1 cannot repair PARylated Top1-DPC as the authors demonstrated, general readers can mis-interpret this interaction as the efficient repair of PARylated Top1-DPC by TDP1. The authors might need to consider removing the paragraph with the subtitle, “PARylation recruits TDP1 to Top1-DPC sites” and Fig.3a and 3b.
8. The authors have to show the PARylation and ubiquitination of Top1-DPC in Fig.4c and 4d (as shown in Fig.2d) in order to demonstrate that the PARylation, but not the loss of ubiquitination, inhibits the physical interaction between Top1-DPC and the proteosome in HEK293 cells. Fig.2d shows the time-course of PARylation and ubiquitination in Top1-DPC during the exposure of HEK293 cells to CPT. Please describe how long did the authors incubate cells with CPT to examine the physical interaction (Fig.4c and 4d).
9. The authors must show the data of alkaline comets in CPT-treated cells in addition to the data of neutral comets (Fig.4f), because alkaline comet tails, but not neutral comet tails, reflect Top1-DPCs.
10. Why did PARGi reduce the extent of neutral comet tails in CPT-treated cells (Fig.4f) although PARGi appeared to completely inhibit the removal of CPT-induced Top1-DPC (Fig.2d)? The authors seem to have mislabeled CPT(+)/PARGi(+) and CPT(+)/PARGi (-) in Fig.4f. Note that the PARGi-dependent suppression of CPT-induced pH2AX (Fig.4g) despite the PARGi-dependent inhibition of Top1-DPC repair is not surprising. This is because intact TOP1 adducts at one-end breaks can interfere with the activation of damage-checkpoint kinases such as ATM and CHK1 at the breakage.
11. This manuscript can be improved if the authors show the effect of USP7i on the prominent de-ubiquitination of Top1-DPC (lane 12 to 14 of Fig.2d) and the generation of one-end breaks in the presence of PARGi by showing CPT-induced pH2AX foci during the S phase. De-ubiquitination of Top1-DPC (2 to 8 h post-addition of CPT in Fig.2d) is an interesting finding but it might affect neither the repair of Top1-DPC nor one-end break formation during the S phase due to a dominant role of Top1 PARylation over Top1 polyubiquitination in the repair of Top1-DPC (Fig.2d).
12. This manuscript will be improved if the authors show the effect of PARGi on CPT-induced pH2AX foci (one-end breakage) during the S phase, considering that PARGi may be a potent anti-cancer agent (Genes Dev. 2020. PMID: 32029455). The number of pH2AX foci is more informative than the western blot analysis of pH2AX (Fig.4g) since pH2AX foci in S phase reflect individual breaks caused by unrepaired Top1-DPCs.
13. Supplementary Table 1 is not included.

Reviewer #2 (Remarks to the Author):

The authors investigate how PARylation of TOP1 regulates the proteolytic processing and repair of TOP1-DPCs. TOP1-DPCs repair by the UPS has intensively been studied by many research groups. PARP1 and PARylation were also investigated in the context of TOP1-DPCs repair and TOP1-DPCs degradation (Lin C et al JBC, 2008). Subhenda et al NAR 2016 showed that TOP1 upon CPT treatment is PARylated. And TOP1 degradation along with its interactions with PARP1/TDP1 are well documented, with PARP inhibition causing increased sensitivity to TOP1-DPCs. Furthermore, the authors published in 2014 (Benu et al NAR 2014), in good mechanistic detail the role of PARP1 and TDP1 in the repair of TOP1. In this manuscript, they claimed that PARP1 is involved in the stabilization of TDP1 via PARylation of TDP1. This is in a similar vein to this submitted manuscript where they claim that PARylation is stabilizing TOP1-DPCs from degradation.
As PARG is the main nuclear enzyme that counteracts PARP1’s PARylation activity and TOP1-DPCs have previously been shown to be PARyated, it is then not surprising that upon PARG inhibition, TOP1 is hyperparylated as both PARP1/PARG are fairly promiscuous in their substrate modifications.
The novelty of this manuscript lies in the mechanistic details of how TOP1-DPCs hyperPARylation affects its repair. When viewed through this framing, the evidence for their proposed mechanism is lacking.

I understand the authors wants to better understand how PARylation regulates TOP1-DPCs repair. However, the presented data are rather premature, not focused and create confusion in the field than helping us to better understand the TOP1-DPCs repair process. The authors used various methods and different approaches to address their questions, but many of the data are not very conclusive or thoroughly investigated.

There is also a conceptual problem which has to be more rigorously addressed. The authors claim that PARylation is important for TDP1 recruitment, the process that occurs downstream of proteasomal degradation, but at the same time the authors claim that PARylation prevents TOP1-DPCs recruitment to the proteasome and their proteolytic degradation. How is it possible to recruit the downstream effector if the upstream factor is blocked? They even mentioned that Top1-DPCs proteasomal-mediated destruction of the bulky protein component of the DPCs is a pivotal step allowing TDP1 to access and hydrolyse otherwise buried Top1-DPC phosphotyrosyl linkage. Finally, the role of USP7 in TOP1-DPCs repair has not been addressed.

Comments:

1) It seems that BTZ strongly affects dynamic of TOP1 and not only reduction of TOP1. It could be that the 26S proteasome is essential for physiological TOP1 turnover and not only TOP1-DPC repair. They also did not analyse how BZT or PARG1 affects untreated cells, what is essential for any conclusion regarding their experiments.

2) Fig2, according to their model that PARylation suppress TOP1-DPCs degradation, inhibition of PARP1 should accelerate TOP1-DPCs repair. This should be tested.
Could PARP1 depletion or inhibition rescue DNA damage after CPT treatment as Fig2d suggests that PARylation prevents Top1-DPCs repair?

3) Fig. 2f does not tell us much, the authors need to show us some kinetics. Suppl. Fig 2d has not been well controlled.

4) Why is the increased in stabilized TOP1-DPCs caused by CPT and PARGI (Figure 2D) causing fewer DSBs (Figure 4f), surely a failure to remove TOP1-DPCs would cause increased DSB. In the results section, the author states that there is a failure in DDR, however would the simpler explanation be that, there is a lower DDR because there is less DNA damage as the data in Figure 2f shows. The authors should address this anomaly, why does PARGi which causes increased TOP1-DPCs, cause less DSBs.

5) The authors observed in Figure 2D, ubiquitylation, deubiquitylation and PARylation of TOP1-DPCs under PARGi. However, the validity of these results are in question as the assay they perform isolated total DPCs from the cell, so to fully validate that they are indeed observing TOP1-DPCs modifications, the same conditions would need to be repeated in TOP1 KO/depleted cell lines. From these data, they explore the role of USP7, in which they show that it indeed is interacting with TOP1-DPCs, increasingly so under PARGi conditions. However the PLA of the interaction in Figure 2D shows that much of the interaction is happening in the cytoplasm, this brings into doubt the relevance of this interaction and the results in Figure 5C.
6) One final point – a review authored by Benu Brata Das (NAR cancer 2021) states “However, the underlying molecular mechanism by which TOP1-PARylation regulates the nuclear mobility of Top1 was mostly obscure until recently, using live-cell microscopy demonstrated that disruption of PARP1 activity by PARP inhibitors delocalized TOP1 from the nucleolus to the nucleoplasm which is independent of the interactions between the two proteins. These studies also suggest that the PARylation of Top1 serves to engage Top1 to the active sites of rDNA and rRNA synthesis.”
This raises the possibility that PARGi is causing the increased relocalization of TOP1-DPCs upon camptothecin, independently of PARP1/TOP1 interaction. And that is why you are causing more TOP1-DPCs, as more TOP1 is being recruited to damage sites. Thus, this phenomenon needs to be ruled out by the authors.

Reviewer #3 (Remarks to the Author):

Sun and colleagues demonstrate the impact of PARylation on ubiquitination-dependent proteasomal degradation of TOP1-DPC and have identified PARG as a repair factor for TOP1-DPCs. Additionally, they have shown that PARylation recruits USP7 to reverse the ubiquitylation of PARylated TOP1-DPCs. This observation is quite novel and will be of broad interest to workers in the DNA repair field. There are some issues that will need to be resolved before this manuscript can be published.

1) In Figure 1, the authors have performed the filming of TOP1-HaloTag in U2OS cells and have shown a drastically reduced single-molecule detection of TOP1 in the presence of CPT, which could be rescued by pretreatment with PARGi. However, it is not clear whether the PARGi considerably increased the mobile fraction of Top1 in the presence of CPT as Poly (ADP-ribose) polymerase1 could facilitate the religation of CPT-induced trapped Top1cc (Park and Cheng, 2005 Can Res). Also, the concentration of CPT used for the experiment seems too high (100 uM for 2 hours). It would be useful to demonstrate that PARG knockdown cells increase CPT-induced trapped Top1cc's.

2). I am confused by the results of the experiments in Figure 2a. The authors show that Top1 is hyper PARylated without DNA damage upon treatment with PARGi. The authors could clarify whether the mobile cellular fraction of Top1 is a substrate for PARylation. I should note that the authors need to measure trapped Top1cc in the presence of PARGi using alternative experiments.

3). The authors show in figure 5, that USP7 reverses Top1 ubiquitylation and increased interaction between the two proteins in the presence of PARGi+CPT. The authors need to test whether USP7 knockdown cells are further sensitized to camptothecin in survival assays.

Reviewer #1 (Remarks to the Author):

DNA topoisomerase I (TOP1) is essential for transcription and DNA replication. Camptothecin (CPT), a TOP1 poison, is widely used for anti-cancer therapy. CPT generates vast numbers of TOP1 DNA-protein crosslinks (TOP1-DPCs), and the resulting single-strand breakage (SSB) is converted to one-end-breaks during DNA replication. Cells deficient in Poly[ADP ribose]polymerase 1 (PARP1) are hypersensitive to CPT (Pommier Y. et al., Nucleic Acids Res. 2014, PMID: 24493735), and the molecular mechanism underlying the hypersensitivity has not yet been fully understood. PARP1 is involved in a number of repair pathways including SSB repair and protects DNA replication forks from one-end-breaks. Sun Y. and Pommier Y. previously reported that the removal of TOP1 adducts from TOP1-DPCs by the TDP1 phosphodiesterase needs preceding partial digestion of TOP1 adducts, "debulking" by the RNF4 ubiquitin ligase and proteasome so that TDP1 can access the crosslink junction (Sci Adv. 2020, PMID: 33188014). The current work revealed that the inhibition of PARG and resulting excess TOP1 PARylation block the debulking of TOP1-DPCs. All the data are impressively robust, and biochemical data agree with cellular experiments including western blot of immune-precipitated samples. This report has made substantial advances in understanding the molecular mechanisms for the two key anti-cancer agents, the TOP1 poison and PARP inhibitor (PARPi). The reviewer, therefore, recommend the publication of this work in Nature Communications. However, general readers could not understand several figures, and the reviewer requests the rewording and additional explanation of the following figures (particularly Fig.2d and Fig.4c and 4d).

Answer: Thank you for finding that “All the data are impressively robust” and “This report has made substantial advances”. Thank you also for suggesting rewording, editing and additional experiments.

Comments:
1. General readers as well as the reviewer are not familiar with the cutting-edge analysis using the single-molecule fluorescence microscopy (Fig.1a), and the authors need to explain the analysis further. The TOP1-Halo-Tag signals of the top panels do not seem to agree with the histogram of the bottom (Fig.1a); although signals of "Before treatment", "CPT + PARGi", and "CPT+BTZ" are similar in the top panels, the histogram shows that the jump of signals "Before treatment" are much higher than that of "CPT + PARGi" and "CPT+BTZ". The authors need to clearly explain the difference in signals between the top and middle panels, "the most mobile TOP1 molecules" shown in the pink area of the histogram, and "jump", the x-axis of the histogram.

Answer: We are sorry for not making the description clearer. As suggested, Figure 1a and the Results have been corrected to clarify the interpretation of the top and middle panels. The top panels show representative snapshot images of TOP1 single molecule 10 second films under the indicated conditions. The middle panels are plots of TOP1 single molecule tracks (2D simulation) over 10 seconds. Tracks of individual TOP1 molecules are shown as lines of different colors as they jump over different distances and different directions. The tracks were reconstructed in two-dimension using a designed MATLAB analysis pipeline. The neighboring tracks are of different colors to distinguish one from the others.

Upon exposure to CPT, which traps TOP1 on chromatin, distances of TOP1 jumps are in general much shorter than for the controls (“Before treatment” and “DMSO”), which is reflected by the shorter tracks for the CPT sample in the middle panel. Also, because CPT induces TOP1 downregulation, the track plot for the CPT group has fewer tracks and these tracks are shorter

than the controls. The track plots for CPT + BTZ and CPT + PARGi have overall the same number of tracks as the controls but these tracks are shorter than the controls, suggesting that although BTZ and PARGi prevented TOP1 downregulation, TOP1 molecules remained trapped on chromatin with limited jumping.

In the bottom panels of Figure 1a, the X axis of the histograms is the jump distance of TOP1 single-molecules and the Y axis the cumulative number of jumps within each given jump distance. The bin size is 0.1 micron. For example, during the 10 second film in the top panel, the “Before treatment” group had ~4,000 jumps whose distances were between 0-0.1 microns and 1,000 jumps whose distances were between 0.7-0.8 microns. The sum of total jumps of TOP1 molecules of the “Before treatment” group is ~19,000. The jumps whose distances are between 0-0.1 microns account for 21% of the total jumps. By contrast, the CPT group showed ~5,000 jumps whose distances were between 0-0.1 micron, and the sum of jumps was 9,000. In this case, jumps of 0-0.1 micron account for 55.6% of the total jumps, indicating that CPT trapped TOP1 and reduced their dynamics. Because TOP1 is downregulated over 50% by CPT, the number of jumps of TOP1 molecules in the CPT group is correspondingly lower than in the controls. The sums of jumps of the CPT + BTZ (~19,000 jumps) and CPT + PARGi (~18,000 jumps) are nearly the same as in the “Before treatment” group. However, CPT + BTZ produced almost 14,000 jumps whose distances were between 0-0.1 microns and CPT + PARG produced 13,500 jumps whose distances were between 0-0.1 microns, which accounts for 73% and 72% of total jumps, respectively. These results demonstrate that although BTZ and PARGi prevent the degradation of TOP1 molecules, the TOP1 molecules are trapped on DNA and cannot move as dynamically as they do in the absence of CPT. The figure legend has been revised accordingly.

In the prior figure, “the mobile TOP1 molecules” and the pink areas were misleading as the plots did not represent TOP1 molecule counts but their jump counts. We have removed them from the figure in our revised manuscript. Thank you.

2. CPT causes the trapping of TOP1 on genomic DNA. Why did the CPT treatment increase the percentage of "the most mobile TOP1 molecules"? Did the treatment with 100 uM CPT for 2 h trap virtually all TOP1 molecules on the genomic DNA? To show the biological significance of "the most mobile TOP1 molecules", the authors need to show the effect of limited concentrations of CPT in a supplementary figure using the method shown in Fig.1a.

Answer: As explained in addressing your prior question, the description of the experiments was unclear and potentially misleading. We have removed this statement.

We did perform single molecule analysis at lower doses of CPT (1 uM and 10 uM) when setting up this technique. However, 10 uM CPT for 2 h did not change t TOP1 single molecules significantly (see figure below), so we increased the dose and observed significant downregulation of TOP1 at 100 uM.

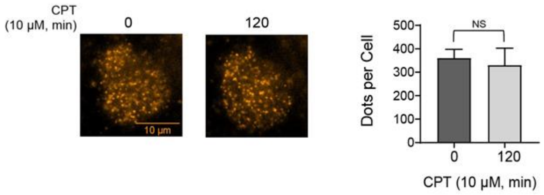

Figure legend: left panel: U2OS cells transfected with TOP1-HaloTag expression plasmid for 24 h were subjected to 10 uM CPT and monitored using single molecule fluorescence microscope at indicated time points. Right panel: TOP1 molecules of the samples in left panel were processed using ImageJ and quantitated using MATLAB analysis pipeline. NS: not significant.

3. The authors need to show that the analysis of Fig.2a was done in a denaturing pull-down condition, but not in a native pull-down, in Figure legend.

Answer: Thank you. Indeed, we performed the experiment in denaturing condition, as described in the Materials & Methods. As suggested, we have added this point to figure legend.

4. The following conclusion, "PARG inhibition reduced the levels of Top1-DPC ubiquitination after 1 hour CPT treatment, suggesting that persistent PARylation of TOP1-DPC leads to their deubiquitination" (line 6th from the bottom of page 4) might not be justified, as the Top1-DPC ubiquitination signal on lane 4 of Fig.2d (without PARG inhibition) is similar to that on lane 11 (with PARG inhibition). Fig.4d seems to show that PARG inhibitor (PARGi) promoted the Top1-DPC formation (at 10 min on lane 9 in the bottom panel), increased and then decreased the ubiquitination earlier than the PARGi(-) samples, and completely prevented the degradation of TOP1. The authors need to reword the conclusion written on line 6th from the bottom of page 4.

Answer: Thank you for the comment. The peak of TOP1-DPC ubiquitylation in the absence of PARGi is 1 h (lane 4 in revised Fig. 2e) whereas the peak of ubiquitylation in the presence of PARG is 30 min (lane 10). The level of ubiquitylation in lane 11 is actually lower than in lane 4. We have reworded the sentence: "However, PARG inhibition facilitated TOP1-DPC ubiquitylation (it peaked at 30 min) and decreased TOP1-DPC ubiquitylation at 1 hour after CPT treatment."

5. Fig.2d indicates the relationship between TOP1 ubiquitination, TOP1 PARylation, and the stability of Top1-DPC and clearly shows that the stability of Top1-DPC depends on the PARylation rather than ubiquitination during the 8 h CPT treatment. The authors need to state a potential negative effect of TOP1 PARylation on the proteosome-mediated degradation of Top1-DPC rather than ""PARG inhibition reduced the levels of Top1-DPC ubiquitination". This statement is helpful for general readers to understand a logical connection between Fig.2d and Fig.4a to 4d, which show that TOP1 PARylation indeed inhibits the digestion of TOP1 by the proteosome in both in vitro (Fig.4b) and in vivo (Fig.4c and d).

Answer: Thank you for your advice. We have added the following sentence to the description of Figure 2e (panel d in previous version) subsection of the Results: "Given the crucial role of the 26S proteasome system for TOP1-DPC repair, we hypothesized that persistent PARylation likely prevents the proteasome-dependent removal of TOP1-DPCs."

6. The authors need to show that the analysis of Fig.3a was done in a native pull-down condition, but not in a denaturing pull-down, in Figure legend.

Answer: Thank you for your suggestion. We performed the experiment in native conditions; otherwise we would not be able to detect TOP1-TDP1 physical interaction. This point, which was described in Methods and Materials, has now been added to the figure legend in our revised manuscript.

7. Fig.3a and 3b show the interaction between intact TOP1 (keeping antigenicity for the western blot analysis) and TDP1. While TDP1 cannot repair PARylated Top1-DPC as the authors demonstrated, general readers can mis-interpret this interaction as the efficient repair of

PARylated Top1-DPC by TDP1. The authors might need to consider removing the paragraph with the subtitle, “PARylation recruits TDP1 to Top1-DPC sites” and Fig.3a and 3b.

Answer: Thank you for your suggestion. We have changed the tile to “TOP1-DPC dePARylation and degradation are required for cellular TDP1 activity” as we performed ICE assay showing that PARGi and bortezomib prevented the TDP1-mediated removal of TOP1-DPC (new Fig. 3c).

8. The authors have to show the PARylation and ubiquitination of Top1-DPC in Fig.4c and 4d (as shown in Fig.2d) in order to demonstrate that the PARylation, but not the loss of ubiquitination, inhibits the physical interaction between Top1-DPC and the proteosome in HEK293 cells. Fig.2d shows the time-course of PARylation and ubiquitination in Top1-DPC during the exposure of HEK293 cells to CPT. Please describe how long did the authors incubate cells with CPT to examine the physical interaction (Fig.4c and 4d).

Answer: We used the same conditions in Fig. 4c and 4d as we did in Fig. 2e (previously Fig. 2d) (20 μM CPT in the same HEK293 cells for 30 min) to examine the interactions of TOP1 and TOP1-DPC with the proteasome. For PARGi treatment, we pre-treated the cells with PARGi (10 μM for 1h, the same conditions were used in Fig. 2e [previously Fig. 2d]), followed by co-treatment with CPT for 30 min. We have included these important points to the Result section. Since at 30 min, the ubiquitylation levels of TOP1-DPCs in the presence of PARGi reached to peak (Fig. 2e, lane 10), we believe that the conclusion we drew from the experiments in Fig, 4c and d indicate that PARylation of TOP1-DPC inhibits their interaction with the proteasome.

9. The authors must show the data of alkaline comets in CPT-treated cells in addition to the data of neutral comets (Fig.4f), because alkaline comet tails, but not neutral comet tails, reflect Top1-DPCs.

Answer: As suggested, we performed alkaline comet assays and observed reduced comet tails reflecting TOP1-induced DNA breaks in the presence of PARGi. This observation is in line with a previous report that inhibiting proteasome abolished the exposure of TOP1-induced DNA breaks (mostly SSBs) (PMID: 18515798, Fig. 4a). Also, another report found by neutral comet assay that inhibiting proteasome also abolished the exposure of TOP1-induced seDSBs (PMID: 19666469, Fig. 1a). These points have been included in our revised manuscript. Thank you.

10. Why did PARGi reduce the extent of neutral comet tails in CPT-treated cells (Fig.4f) although PARGi appeared to completely inhibit the removal of CPT-induced Top1-DPC (Fig.2d)? The authors seem to have mislabeled CPT(+)/PARGi(+) and CPT(+)/PARGi (-) in Fig.4f. Note that the PARGi-dependent suppression of CPT-induced pH2AX (Fig.4g) despite the PARGi-dependent inhibition of Top1-DPC repair is not surprising. This is because intact TOP1 adducts at one-end breaks can interfere with the activation of damage-checkpoint kinases such as ATM and CHK1 at the breakage.

Answer: Thank you for your pointing out our mistake, which we have corrected. The trapped TOP1 holds the broken end and is denatured by lysis buffer that does not contain proteinase K. Therefore, when running electrophoresis, the trapped and unrepaired TOP1 does not impact DNA migration since the nicked DNA is linked to TOP1 and does not come off from genomic DNA. This “intact” DNA becomes DNA breaks when TOP1 is removed, which required dePARylation and degradation. It has been reported that DNA-protein crosslinks cannot be distinguished by normal comet assay unless they are degraded as they do little to DNA migration (PMID: 10217071，PMID: 11080662PMID: 24077345).

This is also why people observe more comet tails (and more gH2AX foci as well) in samples treated CPT for 2h than in samples treated with CPT for 20 min as most of the TOP1-DPCs are removed by proteasomal degradation at 2h whereas TOP1-DPCs peak at 20-30 min after CPT exposure.

To sum up, PARGi results in stabilization and accumulation of full-length TOP1-DPCs by blocking proteasomal degradation thereby prevents the liberation/exposure of TOP1-concealed DNA breaks, which cannot activate DDR and cannot be detected by comet assay.

11. This manuscript can be improved if the authors show the effect of USP7i on the prominent de-ubiquitination of Top1-DPC (lane 12 to 14 of Fig.2d) and the generation of one-end breaks in the presence of PARGi by showing CPT-induced pH2AX foci during the S phase. De-ubiquitination of Top1-DPC (2 to 8 h post-addition of CPT in Fig.2d) is an interesting finding but it might affect neither the repair of Top1-DPC nor one-end break formation during the S phase due to a dominant role of Top1 PARylation over Top1 polyubiquitination in the repair of Top1-DPC (Fig.2d).

Answer: We have done time course (1, 4 h) modified RADAR assays to study the effect of USP7 inhibition and USP knockdown (supplementary fig. 5b and d) and found the inhibition and knockdown led to higher levels of TOP1-DPC ubiquitylation in the presence of PARGi. However, USP7 deficiency and inhibition did not alter the levels of total TOP1-DPCs both in the absence and presence of PARGi. Also, IF using structural illumination microscope showed that USP7 inhibitor had no impact on the formation of CPT-induced gamma H2AX (now Fig. 5f). These findings suggest that USP7 is not involved in the repair of TOP1-DPCs but rather a mechanism to recycle ubiquitin molecules attached onto TOP1-DPCs when the proteasomal deubiquitylating subunit PSMD14 is unable to deubiquitylate TOP1-DPCs, which is dependent on prompt dePARylation.

12. This manuscript will be improved if the authors show the effect of PARGi on CPT-induced pH2AX foci (one-end breakage) during the S phase, considering that PARGi may be a potent anti-cancer agent (Genes Dev. 2020. PMID: 32029455). The number of pH2AX foci is more informative than the western blot analysis of pH2AX (Fig.4g) since pH2AX foci in S phase reflect individual breaks caused by unrepaired Top1-DPCs.

Answer: As suggested, we synchronized U2OS cells by double thymidine block and released them at S phase then performed IF of pH2AX foci using instant structural illumination. (now Fig. 4h). We observed that PARGi completely prevented gammaH2AX formation upon exposure to CPT ($p < 0.001$). This finding is in consistence with the previous reports that blocking proteasome abolished gammaH2AX formation (PMID: 18515798, PMID: 19666469) and suggests the epistasis of PARG and the proteasome for TOP1-DPC repair.

13. Supplementary Table 1 is not included.

Answer: Thank you for noting this editorial error. We included Supplementary Table 1 as Supplementary Dataset when we first submitted the manuscript. We are not sure whether it is not available to the reviewers. We now re-submitted it as Table. Hopefully, this will solve the problem. If you still cannot gain access to the spread sheet, we will ask the editor to send you the original file. Again, we are sorry for bringing any inconvenience.

Reviewer #2 (Remarks to the Author):

The authors investigate how PARylation of TOP1 regulates the proteolytic processing and repair of TOP1-DPCs. TOP1-DPCs repair by the UPS has intensively been studied by many research groups. PARP1 and PARylation were also investigated in the context of TOP1-DPCs repair and TOP1-DPCs degradation (Lin C et al JBC, 2008). Subhenda et al NAR 2016 showed that TOP1 upon CPT treatment is PARylated. And TOP1 degradation along with its interactions with PARP1/TDP1 are well documented, with PARP inhibition causing increased sensitivity to TOP1-DPCs. Furthermore, the authors published in 2014 (Benu et al NAR 2014), in good mechanistic detail the role of PARP1 and TDP1 in the repair of TOP1. In this manuscript, they claimed that PARP1 is involved in the stabilization of TDP1 via PARylation of TDP1. This is in a similar vein to this submitted manuscript where they claim that PARylation is stabilizing TOP1-DPCs from degradation.
As PARG is the main nuclear enzyme that counteracts PARP1's PARylation activity and TOP1-DPCs have previously been shown to be PARyated, it is then not surprising that upon PARG inhibition, TOP1 is hyperparylated as both PARP1/PARG are fairly promiscuous in their substrate modifications.
The novelty of this manuscript lies in the mechanistic details of how TOP1-DPCs hyperPARylation affects its repair. When viewed through this framing, the evidence for their proposed mechanism is lacking.
I understand the authors wants to better understand how PARylation regulates TOP1-DPCs repair. However, the presented data are rather premature, not focused and create confusion in the field than helping us to better understand the TOP1-DPCs repair process. The authors used various methods and different approaches to address their questions, but many of the data are not very conclusive or thoroughly investigated.

<u>Answer</u>: Thank you for your interest and for pointing out these references, which have been included and further discussed in our manuscript. Subhenda Das et al in their interesting and important NAR 2016 paper demonstrates an important role of PARP1-dependent PARylation in regulating the subnuclear localization of TOP1. We cited and discussed this paper in our manuscript.

Our manuscript focuses on the role of PARylation as a regulator of TOP1-DPC processing by the proteasome, which, to our knowledge has not be studied before. Notably, because TOP1-DPC PARylation is extremely transient, none of the published references demonstrated that TOP1-DPCs were PARylated *in vivo*. Also, it is known that the PARylation of chromatin-bound proteins are usually not detectable unless PARG is inhibited (PMID: 29983321). We were able to demonstrate its occurrence and relevance by the addition of PARG inhibitor and by modifying the RADAR assay. We therefore believe that our study shows for the first time the modification of trapped TOP1 (TOP1-DPC) by bulky PAR polymers using the modified RADAR assay and PARG inhibitor. It also demonstrates a novel role of TOP1-DPC PARylation as a regulator of the processing of TOP1-DPC by the proteasome.

We agree with the reviewer's comment that one of the novelties of this manuscript lies in the mechanistic details of how TOP1-DPCs hyperPARylation affects their repair. However, we are not sure why the reviewer states that the evidence for our proposed mechanism is lacking while acknowledging that we used various methods and different approaches to address our questions. Indeed, we have used several new reagents, methods and techniques developed by us and our collaborators to demonstrate that TOP1-DPCs are reversibly and transiently PARylated and that the PARylated DPCs cannot be degraded by the 26S proteasome *in vitro* and *in vivo*. For example, we used single-molecule tracking microscopy, modified RADAR

assays as well as biochemical assays developed by us using purified enzymes including 26S/20S proteasomes, PARP1, TDP1 and biotin/p32-labeled TOP1cc devised by us. All of these reagents and techniques enable us to study the mechanisms in detail and in depth and propose our model that PARylation is a novel regulator of proteasomal degradation of DPCs.

There is also a conceptual problem which has to be more rigorously addressed. The authors claim that PARylation is important for TDP1 recruitment, the process that occurs downstream of proteasomal degradation, but at the same time the authors claim that PARylation prevents TOP1-DPCs recruitment to the proteasome and their proteolytic degradation. How is it possible to recruit the downstream effector if the upstream factor is blocked? They even mentioned that Top1-DPCs proteasomal-mediated destruction of the bulky protein component of the DPCs is a pivotal step allowing TDP1 to access and hydrolyse otherwise buried Top1-DPC phosphotyrosyl linkage. Finally, the role of USP7 in TOP1-DPCs repair has not been addressed.

Answer: Thank you for raising this important point, and for prompting us to clarify the new role of TOP1-DPC PARylation in the context of the established role of PARylation as a recruitment factor for DNA repair enzymes such as TDP1.

TDP1 and the 26S proteasome are not in a signaling axis so we cannot say that one is downstream of the other. In fact, the PARylation-TDP1 and the ubiquitylation-proteasome pathways take place simultaneously, as evidenced by our observation in Fig. 2d. Our data suggest that PARylation and ubiquitylation occur in parallel; so are the recruitments of TDP1 and the proteasome. However, in order for TDP1 to act on TOP1-DPC, the DPC must be proteolyzed (or denatured/unfolded). The PARylation of DPC does not prevent the ubiquitin signaling of proteasome but it shields the DPC as there are likely multiple PARylatable sites on TOP1 (glutamic, aspartic acid and lysine) and the polymers form branches; hence physically blocks the targeting and processing of TOP1-DPC by the proteasome. We don’t think that PARylation prevents the recruitment of the proteasome but it does prevent the binding. This is in part because PAR polymers are sugar rather than peptides, which are not a target of the proteasome, and the 2 megadalton proteasome holoenzyme does not have PAR-binding motifs. This finding is novel and, as proposed in the Discussion, may have implications beyond the processing of TOP1-DPCs.

The purpose of PAR blocking proteasomal targeting and degradation of the DPC may be to ensure successful recruitment of TDP1 to the DPC sites. If the recruitment of TDP1 uses protein modification instead of PAR modification, one can imagine that the protein modification could be prematurely degraded by the proteasome, leading to failure to recruit TDP1. Once TDP1 is recruited, PARylation must be reversed effectively by PARG to ensure timely proteasomal targeting and degradation; and TDP1 hydrolysis following debulking of the TOP1-DPC.

Our data show that the implication of USP7 is to deubiquitylate PARylated TOP1-DPCs in the presence of PARG inhibitor. Inhibiting or knocking down USP7 prevented the decrease in TOP1-DPC ubiquitylation (deubiquitylation) but had no significant impact on the clearance of TOP1-DPCs since inhibiting PARG had already completely blocked the repair of TOP1-DPCs (Fig. 5e; supplementary fig. b and d). Also, USP7 inhibition did not show significant effect on CPT-induced gammaH2AX both in the absence and presence of PARGi (Fig. 5f). We therefore conclude that USP7 does not participate in the resolution of TOP1-DPCs but rather acts as a mechanism to recycle ubiquitin molecules attached to TOP1-DPCs when the proteasomal deubiquitylating subunit PSMD14 of the 2 mDa proteasome holoenzyme is unable to bind and deubiquitylate the PAR polymer-coated TOP1-DPCs.

Comments:

1) It seems that BTZ strongly affects dynamic of TOP1 and not only reduction of TOP1. It could be that the 26S proteasome is essential for physiological TOP1 turnover and not only TOP1-DPC repair. They also did not analyse how BZT or PARG1 affects untreated cells, what is essential for any conclusion regarding their experiments.

Answer: This is a valid point. As suggested, we have examined the effect of BTZ and PARGi on TOP1 dynamics and found that these single agents did not change TOP1 dynamics using single molecule tracking. The data have been included in our revised Figure 1a. Thank you.

2) Fig2, according to their model that PARylation suppress TOP1-DPCs degradation, inhibition of PARP1 should accelerate TOP1-DPCs repair. This should be tested.
Could PARP1 depletion or inhibition rescue DNA damage after CPT treatment as Fig2d suggests that PARylation prevents Top1-DPCs repair?

Answer: This is an interesting point. We examined the effect of PARP inhibition on TOP1-DPC repair and found that TOP1-DPC levels in PARPi-treated cells are higher than those in control cells 1 h after exposure to CPT, consistent with the fact that inhibiting PARP delays the repair of TOP1-DPC likely due to the blockade of recruitment of TDP1, as proposed by Das et al. (2014; 2016). However, TOP1-DPCs in PARPi-treated cells were removed to the same levels as those in control cells after 2 h treatment of CPT, suggesting that alternative nucleases can participate in the repair of TOP1-DPC in the absence of PARP1 and TDP1 after debulking by the proteasome. This point has been included in our revised manuscript (Supplementary 4c).

Importantly, pH2AX, pRPA32 and pCHK1 levels at 1 h CPT treatment in PARPi-treated cells are lower than in control cells, consistent with the proposed mechanism that removal of TOP1-DPCs is required for revelation of the otherwise occluded DNA breaks to activate DNA damage responses. After 4 h CPT treatment, pH2AX, pRPA32 and pCHK1 levels in PARPi-treated cells were higher than those in control cells, suggesting that although TOP1-DPCs are resolved in PARPi-treated cells at this time point, the DNA breaks cannot be efficiently repaired because inhibiting PARP impairs the recruitment of SSB and DSB repair factors.

3) Fig. 2f does not tell us much, the authors need to show us some kinetics. Suppl. Fig 2d has not been well controlled.

Answer: Thank you for your suggestion. We have performed time-course studies and confirmed that replication and transcription are not required for TOP1-DPC PARylation (now Figure 2h). As suggested, we performed time-course study using PARPi (Supplementary 4c) to elaborate the original Suppl. Fig. 2d (now Suppl. Fig. 2i).

4) Why is the increased in stabilized TOP1-DPCs caused by CPT and PARGI (Figure 2D) causing fewer DSBs (Figure 4f), surely a failure to remove TOP1-DPCs would cause increased DSB. In the results section, the author states that there is a failure in DDR, however would the simpler explanation be that, there is a lower DDR because there is less DNA damage as the data in Figure 2f shows. The authors should address this anomaly, why does PARGi which causes increased TOP1-DPCs, cause less DSBs.

Answer: Thank you for the comments. In short, full-length TOP-DPCs conceal the topoisomerase-linked DSBs/SSBs. These topoisomerase-tethered/concealed DNA breaks cannot be sensed by the cell hence do not activate the DDR until the topoisomerase adducts

are proteolyzed. This has been demonstrated by multiple groups including our lab in the context of the effects of proteasome inhibitors on both TOP1- and TOP2-DPC DDR signaling (PMID: 18515798. PMID: 19666469. PMID: 32057297. PMID: 33188014).

Our findings imply that prompt removal of PAR polymer from TOP1-DPCs is a prerequisite for proteasomal degradation of the TOP1-DPCs; hence the exposure of TOP1-occluded DNA breaks, which cannot be detected by comet assay unless the full-length TOP1-DPCs are debulked by proteasome (PMID: 18515798, PMID: 19666469).

In comet assays, the trapped TOP1 holds the broken end and is denatured by lysis buffer that does not contain proteinase K. Therefore, when running electrophoresis, the trapped and unremoved full-length TOP1 adduct does not impact DNA migration since the nicked DNA is linked to TOP1 and does not come off from genomic DNA. This "intact" DNA becomes DNA breaks when TOP1 is removed, which required dePARylation and proteasomal degradation. It has been reported that DNA-protein crosslinks cannot be distinguished by normal comet assay unless they are degraded as they do little to DNA migration (PMID: 10217071， PMID: 11080662PMID: 24077345).

This is also why people observe more comet tails (and more gH2AX foci as well) in samples treated CPT for 2h than in samples treated with CPT for 20 min as most of the TOP1-DPCs are removed by proteasomal degradation at 2h whereas TOP1-DPCs peak at 20-30 min after CPT exposure.

To sum up, PARGi results in stabilization and accumulation of TOP1-DPCs by blocking proteasomal degradation thereby prevents the liberation/exposure of TOP1-concealed DNA breaks, which cannot activate DDR and cannot be detected by comet assay.

5) The authors observed in Figure 2D, ubiquitylation, deubiquitylation and PARylation of TOP1-DPCs under PARGi. However, the validity of these results are in question as the assay they perform isolated total DPCs from the cell, so to fully validate that they are indeed observing TOP1-DPCs modifications, the same conditions would need to be repeated in TOP1 KO/depleted cell lines. From these data, they explore the role of USP7, in which they show that it indeed is interacting with TOP1-DPCs, increasingly so under PARGi conditions. However the PLA of the interaction in Figure 2D shows that much of the interaction is happening in the cytoplasm, this brings into doubt the relevance of this interaction and the results in Figure 5C.

Answer: Thank you for bringing up this valid point. As suggested, we have performed the assays in TOP1 knockdown cells. Under these conditions, we did not observe TOP1-DPC formation and PARylation, suggesting that the PARylation is specific to TOP1-DPC. The PLA assay in Fig. 2 (now Fig. 2b) indeed shows cytoplasmic foci. However, we do believe that these foci were noise as the majority of foci was localized within nucleus. When performing PLA, people inevitably detect background/noise due to the limitation of the technique (1st and 2nd antibody specificity, buffer wash stringency and plasmid overexpression). We are sorry for bringing the confusion. We also repeated the PLA assay for TOP1-USP7 interaction (Now Fig. 5d) and observed that the majority of the interactions was in nucleus.

6) One final point – a review authored by Benu Brata Das (NAR cancer 2021) states "However, the underlying molecular mechanism by which TOP1-PARylation regulates the nuclear mobility of Top1 was mostly obscure until recently, using live-cell microscopy demonstrated that disruption of PARP1 activity by PARP inhibitors delocalized TOP1 from the nucleolus to the nucleoplasm which is independent of the interactions between the two proteins. These studies

also suggest that the PARylation of Top1 serves to engage Top1 to the active sites of rDNA and rRNA synthesis."
This raises the possibility that PARGi is causing the increased relocalization of TOP1-DPCs upon camptothecin, independently of PARP1/TOP1 interaction. And that is why you are causing more TOP1-DPCs, as more TOP1 is being recruited to damage sites. Thus, this phenomenon needs to be ruled out by the authors.

Answer: We are aware and carefully read this insightful and comprehensive review and cited it in our manuscript. We performed subcellular fractionation assay and found that PARGi did not change the pattern of TOP1 chromatin localization both in the absence and presence of CPT. The new data have been added in our revised Supplementary Figure 2h. Thank you.

Reviewer #3 (Remarks to the Author):

Sun and colleagues demonstrate the impact of PARylation on ubiquitination-dependent proteasomal degradation of TOP1-DPC and have identified PARG as a repair factor for TOP1-DPCs. Additionally, they have shown that PARylation recruits USP7 to reverse the ubiquitylation of PARylated TOP1-DPCs. This observation is quite novel and will be of broad interest to workers in the DNA repair field.

Answer: Thank you for finding the observation novel and of broad interest.

There are some issues that will need to be resolved before this manuscript can be published.
1) In Figure 1, the authors have performed the filming of TOP1-HaloTag in U2OS cells and have shown a drastically reduced single-molecule detection of TOP1 in the presence of CPT, which could be rescued by pretreatment with PARGi. However, it is not clear whether the PARGi considerably increased the mobile fraction of Top1 in the presence of CPT as Poly (ADP-ribose) polymerase1 could facilitate the religation of CPT-induced trapped Top1cc (Park and Cheng, 2005 Can Res). Also, the concentration of CPT used for the experiment seems too high (100 uM for 2 hours). It would be useful to demonstrate that PARG knockdown cells increase CPT-induced trapped Top1cc's.

Answer: Thank you for this interesting point. As suggested, we have examined the potential effects of BTZ and PARGi on TOP1 dynamics and found that these single agents did not change TOP1 dynamics using single molecule tracking. These new data have been included in our revised Figure 1. We indeed tested lower concentrations of CPT. We used 10 uM, the concentration we normally use, and found that CPT treatment had not significantly impact on the levels of TOP1 single molecules (see figure below). This is why we increased the concentration to 100 uM.

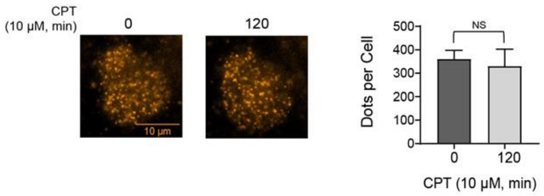

Figure legend: left panel: U2OS cells transfected with TOP1-HaloTag expression plasmid for 24 h were subjected to 10 uM CPT and monitored using single molecule fluorescence microscope at indicated time points. Right panel: TOP1 molecules of the samples in left panel were processed using ImageJ and quantitated using MATLAB analysis pipeline. NS: not significant.

2). I am confused by the results of the experiments in Figure 2a. The authors show that Top1 is hyper PARylated without DNA damage upon treatment with PARGi. The authors could clarify whether the mobile cellular fraction of Top1 is a substrate for PARylation. I should note that the authors need to measure trapped Top1cc in the presence of PARGi using alternative experiments.

Answer: This is a valid point. As suggested, we performed TOP1 His-pulldown assays in the absence of CPT using His-TOP1 WT and His-TOP1 catalytic dead (Y723F) and found that

TOP1 WT but not Y723F was PARylated in the presence of PARGi (now Supplementary Fig. 2a). This result raises the possibility that TOP1 overexpression may result in low levels of spontaneous TOP1-DPCs that undergo PARylation.

As suggested, we also measured TOP1cc using in vivo complex of enzyme assay by cesium chloride ultracentrifugation (ICE) in PARG siRNA-transfected cells and observed stabilization of TOP1-DPC (now Fig. 2F). This point has been added in our revised manuscript. Thank you.

3). The authors show in figure 5, that USP7 reverses Top1 ubiquitylation and increased interaction between the two proteins in the presence of PARGi+CPT. The authors need to test whether USP7 knockdown cells are further sensitized to camptothecin in survival assays.

Answer: As suggested, we have performed survival assays in HEK293 and U2OS cells treated with CPT +/- USP7 inhibitor and found that USP7i did not sensitize cells to CPT. These results indicate that USP7 is not a rate limiting factor for the repair of TOP1-DPCs. It is likely a mechanism to recycle ubiquitin when the ubiquitylated PAR-DPC substrate cannot be processed and recycled by the proteasome and/or as they may still be capable to self-reverse eventually. We do believe hyperPARylation is a signal that evokes USP7 as PARP inhibitor blocks the interaction between TOP1 and USP7. These points have been added to the Discussion of our revised manuscript.

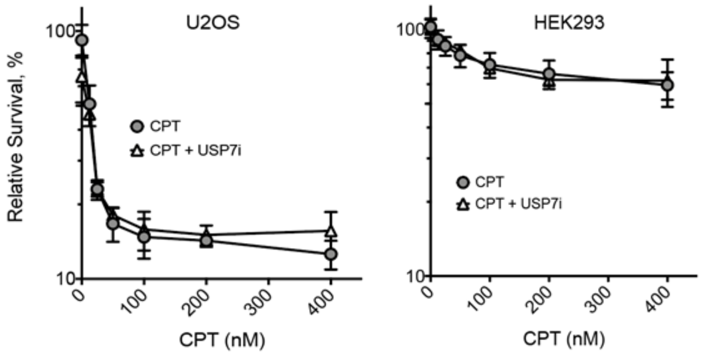

Figure legend: 10,000 U2OS and HEK293 cells were seeded in 96-well white plates in triplicate in 100 ul of medium per well overnight. The next day, cells were exposed to CPT at indicated concentrations +/- USPi (100 nM) and incubated for 72 hours Cellular viability was determined using the ATPlite 1-step kits.

We have also assessed TOP1-DPC ubiquitylation in USP7 KD cells in the presence of PARG inhibitor and found that USP7 KD cells displayed higher levels of TOP1-DPC ubiquitylation, in consistence with our findings using USP7 inhibitor. These data have been added to our revised manuscript (now Supplementary Fig. 5d).

REVIEWERS' COMMENTS

Reviewer #1 (Remarks to the Author):

The major claim of the manuscript (NCOMMS-21-02611A) is the inhibition of proteasome-mediated digestion of TOP1 trapped at DNA cleavage. The authors have properly addressed all questions raised by the previous review. The reviewer, therefore, recommends the publication of this manuscript in the current form.

Reviewer #2 (Remarks to the Author):

The authors have addressed the majority of my concerns. The manuscript has been significantly improved, and therefore I support its acceptance for publication now.

Reviewer #3 (Remarks to the Author):

The authors have satisfactorily addressed my initial quires and I recommend for publication.
